# Synthesis, trehalase hydrolytic resistance and inhibition properties of 4- and 6-substituted trehalose derivatives

**DOI:** 10.1080/14756366.2020.1837125

**Published:** 2020-11-08

**Authors:** Shari Dhaene, Johan Van der Eycken, Koen Beerens, Jorick Franceus, Tom Desmet, Jurgen Caroen

**Affiliations:** a Department of Biotechnology, Centre for Synthetic Biology, Ghent University, Ghent, Belgium; b Department of Organic and Macromolecular Chemistry, Laboratory for Organic and Bio-Organic Synthesis (LOBOS), Ghent University, Ghent, Belgium

**Keywords:** Trehalose derivatives, trehalase, inhibition, hydrolytic resistance, molecular dockings

## Abstract

Although trehalose has recently gained interest because of its pharmaceutical potential, its clinical use is hampered due to its low bioavailability. Hence, hydrolysis-resistant trehalose analogues retaining biological activity could be of interest. In this study, 34 4- and 6-*O*-substituted trehalose derivatives were synthesised using an ether- or carbamate-type linkage. Their hydrolysis susceptibility and inhibitory properties were determined against two trehalases, i.e. porcine kidney and *Mycobacterium smegmatis*. With the exception of three weakly hydrolysable 6-*O*-alkyl derivatives, the compounds generally showed to be completely resistant. Moreover, a number of derivatives was shown to be an inhibitor of one or both of these trehalases. For the strongest inhibitors of porcine kidney trehalase IC_50_ values of around 10 mM could be determined, whereas several compounds displayed sub-mM IC_50_ against *M. smegmatis* trehalase. Dockings studies were performed to explain the observed influence of the substitution pattern on the inhibitory activity towards porcine kidney trehalase.

**ARTICLE HISTORY**
Received 7 September 2020
Revised 5 October 2020
Accepted 9 October 2020

## Introduction

Carbohydrates are the most diverse and abundant biomolecules on earth, displaying a wide variety of functions[1]. During the last decades, interest arose in trehalose **1** (Figure 1), a disaccharide consisting of two D-glucose units linked via an α-1,1-α-bond, resulting in a molecule with unique stabilising properties[2,3]. The stability of this non-reducing glucobiose is reflected by several interesting physicochemical properties like broad pH stability[4], high glass transition and melting temperatures[5,6], low hydrolysis rate[5]. During heating and processing of food products acrylamide formation can be suppressed by trehalose as it interacts with glucose and thus reduces the glucose-Asn reaction leading to the toxic compound[4,6]. In nature, trehalose fulfils a biological role in various organisms including bacteria, yeast, fungi, insects, invertebrates and plants, although it is not found in mammals[6,7]. It can serve as energy and carbon source[6,7], signalling molecule[6,8,9] and cell wall building block[6,10]. Furthermore, it can protect organisms and compounds under stress conditions like desiccation[7,11,12], dehydration[7,13–15], heat[7,13], cold[7] and oxidation[7,14].

Since the establishment of the Hayashibara process, the availability of trehalose has drastically increased, leading to its widespread use in the food, cosmetic and pharmaceutical industry[6]. Moreover, due to its safeguarding capabilities, trehalose has received attention as a chemical chaperone[16,17] and autophagy inducer[16] in the treatment of neurodegenerative diseases like Alzheimer's[18,19], Huntington's[20] and Parkinson's[16,21,22]. Unfortunately, the therapeutic use of trehalose is hampered due to its low bioavailability. Indeed, this disaccharide is rapidly degraded into glucose by the trehalase (EC 3.2.1.28) present in our small intestine[23]. Trehalose analogues and derivatives that show resistance against this human intestinal enzyme could, therefore, be of great interest for pharmaceutical formulations and for drug discovery due to their increased residence time in the human body[3,23]. Recently, a trehalose analogue substituted on the 4-hydroxy group, lentztrehalose A **2** (Figure 1), was isolated from the actinomycete *Lentzea sp.* and was shown to be only weakly hydrolysed by porcine kidney trehalase[24]. Furthermore, it also exhibited antitumor activity[24]. Subsequently, Wada and co-workers isolated two additional natural analogues, lentztrehalose B **3** and C **4** (Figure 1), which were shown to be possible inducers of autophagy[25]. Importantly, these lentztrehaloses were found not to be digested in a range of microbes and cancer cell lines, and their *in vivo* bioavailability and stability was confirmed after oral administration to mice[26].

In this study, an exploration of 34 trehalose derivatives (including 33 new compounds next to lentztrehalose A 2) was established. Inspired by the lentztrehaloses A-C **2**–**4**, a series of 4-*O*-substituted trehalose derivatives was synthesised using an ether- or carbamate-type linkage. In addition, the analogous 6-*O*-substituted series was explored while some double substituted derivatives were also investigated. Their biological relevance was assessed by measuring their hydrolysis (as substrate) and binding (as inhibitor) with two relevant trehalases, i.e. porcine kidney trehalase (91% similarity with human trehalase) and *Mycobacterium smegmatis* trehalase (93% similarity with *M. tuberculosis* trehalase). These enzymes are classified in family GH37 and GH15, respectively, of the CAZy-classification. Although they adopt a similar

CONTACT Tom Desmet tom.desmet@ugent.be Department of Biotechnology, Centre for Synthetic Biology (CSB), Ghent University, Ghent, Belgium
Supplemental data for this article is available online at here.

**1**
trehalose

**2**
lentztrehalose A

**3**
lentztrehalose B

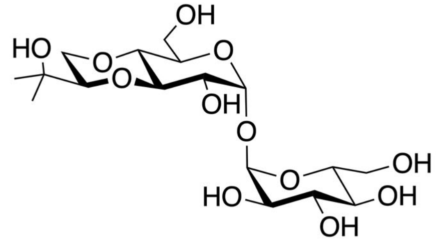

**4**
lentztrehalose C

**Figure 1.** Structure of trehalose **1** and lentztrehalose A-C **2–4**.

overall fold (i.e. an $(\alpha/\alpha)_6$-barrel) and follow the same general reaction mechanism (i.e. inversion of the anomeric configuration through single displacement), they are otherwise unrelated (about 20% similarity). Computational studies were performed to clarify the structural determinants behind the activities and selectivity profiles of the studied trehalose derivatives towards porcine kidney trehalase.

## Material and methods

### Chemistry

#### General remarks

All reactions, unless otherwise stated, were carried out under argon atmosphere in dry solvents. Dichloromethane and triethylamine were freshly distilled from $CaH_2$. Toluene was freshly distilled from Na. Tetrahydrofuran was freshly distilled from Na/benzophenone. Other solvents and reagents were obtained from commercial sources and were used as received without further purification. Flash chromatography was carried out with Rocc silicagel 60 Å, 40–63 µm. Precoated silica gel plates (Macherey-Nagel SIL G-25 $UV_{254}$) were used for TLC employing UV-absorption at 254 nm and $Mo_7O_{24}$/$Ce(SO_4)_2$/aq.$H_2SO_4$ staining for visualisation. Electrospray mass spectra were recorded on an Agilent 1100 series single quadrupole MS detector type VL with an APCI source and an API-ES source, provided with a Phenomenex Luna C18 (2), 5 µm 250 mm × 4.60 mm column. High resolution mass spectrometry (HRMS) was performed on an Agilent 1100 series connected to a 6220 A TOF-MS detector equipped with an APCI-ESI multimode source. $^1$H-NMR and $^{13}$C-NMR spectra (see Supplementary information) were recorded on a Bruker Avance 300, Bruker Avance 400 or a Bruker AM 500 spectrometer as indicated with chemical shifts reported in parts per million, referenced to the residual solvent signals ($CDCl_3$: 7.26 and 77.00 ppm, $D_2O$: 4.75 ppm, $CD_3OD$: 3.31 and 49.15 ppm, benzene-$d_6$: 7.16 and 128.0 ppm, DMSO-$d_6$: 2.50 and 39.43 ppm, acetone-$d_6$: 2.05 and 29.84 ppm). $^{13}$C-NMR Spectra recorded in $D_2O$ were referenced to the signal (30.89 ppm) of acetone (1 drop added). Coupling constants, *J*, are reported in hertz (Hz). Infra-red spectra were recorded on a Perkin-Elmer 1000 FT-IR infra-red spectrometer (horizontal attenuated total reflection (HATR)). Optical rotation was measured on a Perkin Elmer 241 Polarimeter.

#### General procedure A: O-alkylation

To a solution of an OH-containing starting material in anhydrous DMF (0.1 M concentration) was added NaH (60% in mineral oil, 2.5 eq for each hydroxyl group). After stirring for 15 min, the alkyl halogenide (4 eq for each hydroxyl group) was added and the reaction mixture was stirred overnight. Methanol (5 ml per mmol starting material) was added dropwise (caution: gas formation) and the mixture was transferred to a separation funnel using EtOAc (50 ml per mmol starting material). The organic layer is washed with brine (5×, 50 ml per mmol starting material) and concentrated under reduced pressure. The residue was purified by column chromatography.

#### General procedure B: carbamate formation

To a solution of an OH-containing starting material in $CH_2Cl_2$ (0.1 M concentration) was added DMAP (0.2 eq for each hydroxyl group) and the isocyanate (3 eq for each hydroxyl group). The reaction mixture was stirred at room temperature until the consumption of starting material (TLC monitoring, 24–120 h). The mixture was concentrated under reduced pressure and the residue was purified by column chromatography.

#### General procedure C: hydrogenolysis

To a solution of a benzyl/benzylidene-protected starting material in EtOAc/MeOH (1/1, 0.05 M concentration), Pd/C (10% w/w Pd,

0.05 eq) was added. A $H_2$ balloon was placed and the reaction mixture was stirred overnight after which it was filtered over celite. The filtrate was concentrated under reduced pressure and the residue was purified by column chromatography.

### *2,3,4,2′,3′-Penta-O-benzyl-4′,6′-O-benzylidene-α,α-D-trehalose (7)*

To a cooled (−18 °C) solution of **6**[29] (5.576 g, 6.35 mmol, 1 eq) in toluene (56 ml), DIBAL-H (supplied as 1.0 M solution in toluene, 31.9 ml, 31.9 mmol, 5 eq) was added dropwise. The cooling bath was removed and the reaction mixture was stirred at room temperature for 90 min. The solution was cooled to 0 °C and the reaction was quenched by dropwise addition of MeOH (11.5 ml) and aqueous KOH (10% w/v, 3.75 ml). The resulting suspension was transferred to a separation funnel using $CH_2Cl_2$ (400 ml) and $H_2O$ (400 ml). The organic layer was separated and the aqueous phase was extracted using $CH_2Cl_2$ (3 × 400 ml). The combined organic layers were dried on $MgSO_4$, the drying agent was filtered and the filtrate was concentred under reduced pressure. The residue was purified by flash column chromatography (gradient elution: hexane/EtOAc 8/2 to 1/1) to obtain the title compound **7** as a white foam with a yield of 80% (4.443 g; 5.05 mmol). Spectral data were in agreement with literature[27].

### *2,3,6,2′,3′-Penta-O-benzyl-4′,6′-O-benzylidene-α,α-D-trehalose (8) and 2,3,6,2′,3′,6′-hexa-O-benzyl-α,α-D-trehalose (9)*

To a cooled (0 °C) solution of **6**[27] (2.000 g, 2.28 mmol, 1 eq) in $CH_2Cl_2$ (2.0 ml), DIBAL-H (1.0 M solution in $CH_2Cl_2$, 11.4 ml, 11.4 mmol, 5 eq) was added dropwise. After stirring for 90 min at 0 °C, the clear colourless reaction mixture was diluted with $CH_2Cl_2$ (20 ml) and the reaction was quenched by dropwise addition of MeOH (6 ml). Subsequently, aqueous KOH (10% w/v, 6 ml) was added and the resulting suspension was transferred to a separation funnel using $CH_2Cl_2$ (250 ml) and $H_2O$ (250 ml). The organic layer was separated and the aqueous phase was extracted with $CH_2Cl_2$ (2 × 250 ml). The combined organic layers were dried on $MgSO_4$, the drying agent was filtered and the filtrate was concentrated under reduced pressure. The residue was purified using column chromatography (gradient elution: hexane/EtOAc 8/2 to 6/4). Following products were isolated (in order of elution): recovered starting material **6** (200 mg, 0.228 mmol, 10%), compound **8** (4-OH, 1.107 g, 1.256 mmol, 55%), compound **9** (4/4′-bis-OH, 179 mg, 0.203 mmol, 9%) and compound **7** (6-OH, 380 mg, 0.431 mmol, 19%). Rf values in hexane/EtOAc 7/3: Rf 0.36 (**6**), Rf 0.31 (**8**), Rf 0.25 (**9**), Rf 0.11 (**7**). Spectral data of compounds **8**[28] and **9**[29] were in agreement with the literature.

### *2,3,4,2′,3′,4′-Hexa-O-benzyl-α,α-D-trehalose (10)*

To a cooled solution (0 °C) of **7** (620 mg, 0.704 mmol, 1 eq) in toluene (6.32 ml), DIBAL-H (1.0 M in toluene, 3.52 ml, 3.52 mmol, 5 eq) was added dropwise. After 15 min, the ice bath was removed and the reaction mixture was stirred for 24 h. After cooling to 0 °C, MeOH (1.3 ml) and 10% aqueous KOH (0.4 ml) were added, and the resulting mixture was transferred to a separation funnel using $CH_2Cl_2$ (40 ml) and $H_2O$ (40 ml). The organic layer was separated and the aqueous layer was extracted with $CH_2Cl_2$ (2 × 40 ml). The combined organic layers were washed with brine (50 ml) and concentrated under reduced pressure. The residue was purified via column chromatography (gradient elution: hexane/EtOAc 7/3 to 4/6), affording the title compound **10** as a white foam (491 mg, 0.556 mmol, 79%). Rf 0.14 in hexane/EtOAc 1/1. Spectral data were in agreement with the literature[28].

### *2,3,4,6,2′,3′-Hexa-O-benzyl-4′,6′-O-benzylidene-α,α-D-trehalose (11)*

*Method 1: via benzylation of **7***. To a solution of **7** (414 mg, 0.470 mmol) in anhydrous DMF (4.7 ml), NaH (60% in mineral oil, 78 mg, 1.17 mmol, 2.5 eq) was added slowly resulting in gas formation. The grey suspension was cooled to 0 °C and BnBr (113 µL, 0.94 mmol, 2 eq) was added dropwise. Next, TBAI (12 mg, 0.032 mmol, 0.07 eq) was added and the ice bath was removed. After stirring overnight, the reaction mixture was cooled to 0 °C and carefully quenched (gas formation) with MeOH (2 ml). After 15 min, the mixture was diluted with ethyl acetate (35 ml) and transferred to a separation funnel. The organic phase was washed with brine (3 × 30 ml), and dried on $MgSO_4$. The drying agent was filtered and the filtrate was concentrated under reduced pressure. The crude product was purified by flash column chromatography (gradient elution: hexane/EtOAc 9/1 to 8/2) giving the title compound **11** as a white solid (yield: 73%, 328 mg). Spectral data were in agreement with literature[29].

*Method 2: alternative route via **14***. To a suspension of anhydrous trehalose (15 g, 43.82 mmol) and camphorsulfonic acid (0.70 g, 2.19 mmol, 0.05 eq) in DMF (90 ml) benzaldehyde dimethyl acetal (6.57 ml, 43.82 mmol, 1 eq) was added and the mixture was heated to 90 °C for 15 min. To the resulting colourless solution, triethylamine (6.90 ml, 49.5 mmol, 1.1 eq) was added and the mixture was concentrated under reduced pressure. The crude 4,6-*O*-benzylidene-α,α-D-trehalose (**13**) (21.17 g) was dissolved in pyridine/acetic anhydride (1/1, 80 ml) and the reaction mixture was stirred for 24 h after which the volatiles are removed under reduced pressure. The crude product was purified by column chromatography (gradient eluent ion hexane/EtOAc 8/2 to 6/4) giving 2,3,4,6,2′,3′-hexa-*O*-acetyl-4′,6′-*O*-benzylidene-α,α-D-trehalose (**14**) as a white solid with a yield of 34% over two steps (10.17 g, 14.90 mmol).

**14**: Rf 0.28 in hexane/EtOAc 1/1. $^1H$ NMR (400 MHz, acetone-$d_6$): $\delta$ 7.48–7.42 (m, 2H), 7.38–7.33 (m, 3H), 5.67 (s, 1H), 5.60 (t, $J = 9.9$ Hz, 1H), 5.53 (t, $J = 9.5$ Hz, 1H), 5.40 (d, $J = 3.9$ Hz, 1H), 5.34 (d, $J = 3.8$ Hz, 1H), 5.10–5.00 (m, 3H), 4.28–4.15 (m, 3H), 4.11–4-03 (m, 2H), 3.94 (t, $J = 9.6$ Hz, 1H), 3.86 (t, $J = 10.3$ Hz, 1H), 2.12 (s, 3H), 2.11 (s, 3H), 2.02 (s, 6H), 2.01 (s, 3H), 1.98 (s, 3H) ppm. $^{13}C$ NMR (100 MHz, acetone-$d_6$): $\delta$ 170.7 (C), 170.5 (C), 170.4 (C), 170.3 (C), 170.1 (C), 170.1 (C), 138.5 (C), 129.8 (CH), 128.9 (CH), 127.2 (CH), 102.4 (CH), 94.0 (CH), 92.9 (CH), 79.4 (CH), 71.4 (CH), 70.9 (CH), 70.6 (CH), 69.6 (CH), 69.4 (CH), 69.4 (CH), 69.0 ($CH_2$) 64.5 (CH), 62.9 ($CH_2$), 20.7 ($CH_3$), 20.6 ($CH_3$), 20.6 ($CH_3$), 20.6 ($CH_3$) ppm. IR (HATR): 2964 (w), 1741 (s), 1374 (m), 1229 (s), 1214 (s), 1160 (w), 1134 (m), 1062 (m), 1035 (m), 997 (m), 982 (m), 956 (m), 920 (w), 907 (m), 803 (w), 767 (w), 729 (w), 702 (w), 654 (w) $cm^{-1}$. ESMS [m/z (fragment, intensity), positive mode]: 700.2 ($M + NH_4^+$, 100). HRMS (ESI-TOF): calcd. for $C_{31}H_{42}NO_{17}^+$ $[M + NH_4]^+$ 700.2447; found 700.2444.

To a suspension of **14** (1 g, 1.465 mmol) in MeOH (15 ml), NaOMe (0.950 g, 17.58 mmol, 12 eq) was added. The reaction mixture was stirred for 1 h after which silica (1 g) was added. The suspension was filtered and the filtrate was concentrated under reduced pressure. Purification by flash column chromatography (eluent $CH_2Cl_2$/MeOH 8/2 to 6/4) gave 4,6-*O*-benzylidene-α,α-D-trehalose (**13**) as a white solid (831 mg), but still containing an impurity according to TLC.

**13** (purified sample): Rf 0.60 in acetonitrile/water 8/2. $^1H$ NMR (400 MHz, $CD_3OD$): $\delta$ 7.51–7.43 (m, 2H), 7.37–7.26 (m, 3H), 5.56 (s, 1H), 5.16 (d, $J = 4.0$ Hz, 1H), 5.08 (d, $J = 3.8$ Hz, 1H), 4.21 (dd, $J = 9.8/4.9$ Hz, 1H), 4.13–4.03 (m, 1H), 4.00 (t, $J = 9.4$ Hz, 1H), 3.90–3.64 (m, 5H), 3.60 (dd, $J = 9.4/4.0$ Hz, 1H), 3.49 (dd, $J = 9.8/3.6$ Hz, 1H), 3.47 (t, $J = 9.6$ Hz, 1H), 3.37–3.30 (m, 2H) ppm.

$^{13}C$ NMR (100 MHz, $CD_3OD$): $\delta$ 139.2 (C), 129.9 (CH), 129.0 (CH), 127.5 (CH), 103.0 (CH), 95.9 (CH), 95.5 (CH), 83.1 (CH), 74.5 (CH), 73.9 (CH), 73.8 (CH), 73.2 (CH), 71.8 (CH), 71.5 (CH), 70.0 ($CH_2$), 64.1 (CH), 62.6 ($CH_2$) ppm. IR (HATR): 3318 (m, br), 2982 (w), 2930 (w), 2868 (w), 1592 (m), 1455 (w), 1375 (m), 1345 (m), 1148 (m), 1122 (m), 1111 (m), 1071 (m), 1047 (m), 1024 (m), 1004 (m), 981 (s), 927 (w), 838 (w), 800 (w), 760 (w), 702 (w) $cm^{-1}$. ESMS [m/z (fragment, intensity), API-ES negative mode]: 429.1 (M-$H^+$, 100). HRMS (ESI-TOF): calcd. for $C_{19}H_{26}NaO_{11}^+$ $[M+Na]^+$ 453.1367; found 453.1374. Literature data are available[29].

To a solution of crude **13** in anhydrous DMF (12 ml), NaH (60% in mineral oil, 700 mg, 17.4 mmol) was added slowly (gas formation). After stirring for 15 min, the resulting suspension was cooled to 0 °C and BnBr (1.67 ml, 13.9 mmol) was added dropwise, followed by TBAI (129 mg, 0.348 mmol). The ice bath was removed and the reaction mixture was stirred overnight. After cooling to 0 °C, MeOH (5 ml) was added (gas formation is observed) and the mixture is stirred for 10 min after which it is diluted with ethyl acetate (40 ml). The organic layer was washed with brine (5 × 40 ml), and dried on $MgSO_4$. The drying agent was filtered and the filtrate was concentrated under reduced pressure. The crude product was purified by flash column chromatography (gradient elution: hexane/EtOAc 95/5 to 8/2) giving **11** as a white solid with a yield of 51% over two steps (725 mg, 0.747 mmol).

### *2,3,4,6,2′,3′,6′-Hepta-O-benzyl-α,α-D-trehalose (12)*

To a solution of **11** (5.055 g; 5.205 mmol) in anhydrous THF (52 ml), molecular sieves (4 Å, 5.00 g) were added. After stirring for 30 min, $Me_3N$-$BH_3$ (4.556 g; 62.46 mmol, 12 eq) was added. The resulting mixture was cooled to 0 °C and $AlCl_3$ (8.328 g, 65.46 mmol, 12 eq) was added. The cooling bath was removed and the reaction mixture was stirred overnight. The mixture was diluted with EtOAc (1.6 L) and transferred to a flask containing a solution of cold 1% aq $H_2SO_4$ (800 ml, containing ice). After stirring for 30 min, the mixture was transferred to a separation funnel, the organic layer was separated and the aqueous phase was extracted with EtOAc (3 × 750 ml). The combined organic layers were washed with saturated $NaHCO_3$ solution (3 × 2.4 L) and brine (3 × 2.4 L). The organic phase was dried on $MgSO_4$, the drying agent was filtered, and the filtrate was concentrated under reduced pressure. The residue was purified by flash column chromatography (100% toluene, followed by gradient elution: hexane/EtOAc 9/1 to 75/25), giving the title compound **12** as a colourless oil (49%, 2.45 g, 2.52 mmol). Spectral data were in agreement with literature[30]. In addition, the corresponding 6-OH regioisomer (2,3,4,6,2′,3′,4′-hepta-*O*-benzyl-α,α-D-trehalose) was isolated (1.90 g, 1.95 mmol, 38%). Spectral data were in agreement with literature[31].

### *2,3,4,6,2′,3′-Hexa-O-benzyl-α,α-D-trehalose (15)*

To a solution of **11** (50 mg, 0.051 mmol) in $CH_2Cl_2$/MeOH (1/3, 1.2 ml) is added trifluoroacetic acid (0.1 ml) and the resulting reaction mixture is stirred for 5 days. The mixture is diluted with $CH_3CN$ and concentrated under reduced pressure. The residue is purified via column chromatography (gradient elution: hexane/EtOAc 1/1 to 4/6) to give the title compound **15** as a colourless glass (34 mg, 0.039 mmol, 76%).

Rf 0.34 in hexane/EtOAc 4/6. $^1H$ NMR (400 MHz, $CDCl_3$): $\delta$ 7.43–7.20 (m, 28H), 7.18–7.11 (m, 2H), 5.24 (d, $J = 3.5$ Hz, 1H), 5.21 (d, $J = 3.5$ Hz, 1H), 5.03 (d, $J = 11.5$ Hz, 1H), 5.01 (d, $J = 10.9$ Hz, 1H), 4.89 (d, $J = 10.9$ Hz, 1H), 4.84 (d, $J = 10.7$ Hz, 1H), 4.78 (d, $J = 11.5$ Hz, 1H), 4.76–4.63 (m, 4H), 4.55 (d, $J = 12.1$ Hz, 1H), 4.48 (d, $J = 10.7$ Hz, 1H), 4.39 (d, $J = 12.1$ Hz, 1H), 4.20–4.13 (m, 1H), 4.10–3.98 (m, 2H), 3.89 (app t, $J = 9.3$ Hz, 1H), 3.75–3.47 (m, 7H), 3.40 (dd, $J = 10.7/1.9$ Hz, 1H) ppm. $^{13}C$ NMR (100 MHz, $CDCl_3$): $\delta$ 138.8 (C), 138.7 (C), 138.3 (C), 138.0 (C), 137.9 (C), 137.8 (C), 128.5 (CH), 128.4 (CH), 128.4 (CH), 128.4 (CH), 128.3 (CH), 128.0 (CH), 127.9 (CH), 127.9 (CH), 127.8 (CH), 127.8 (CH), 127.7 (CH), 127.7 (CH), 127.6 (CH), 127.6 (CH), 127.3 (CH), 94.2 (CH), 94.0 (CH), 81.8 (CH), 80.9 (CH), 79.3 (CH), 79.2 (CH), 77.7 (CH), 75.6 ($CH_2$), 75.2 ($CH_2$), 75.0 ($CH_2$), 73.5 ($CH_2$), 72.9 ($CH_2$), 72.5 ($CH_2$), 71.2 (CH), 70.7 (CH), 70.5 (CH), 68.2 ($CH_2$), 62.2 ($CH_2$) ppm. IR (HATR): 3451 (br, m), 3059 (w), 3030 (w), 2926 (w), 2868 (w), 1496 (w), 1453 (m), 1360 (m), 1326 (w), 1272 (w), 1208 (w), 1153 (m), 1091 (m), 1054 (s), 1026 (m), 991 (s), 918 (w), 849 (w), 799 (w), 733 (s), 695 (s), 638 (w) $cm^{-1}$. ESMS [m/z (fragment, intensity), API-ES positive mode]: 900.3 ($M+NH_4^+$, 100). HRMS (ESI-TOF): calcd. for $C_{54}H_{62}NO_{11}^+$ $[M+NH_4]^+$ 900.4317; found 900.4321.

### *2,3,4,2′,3′-Penta-O-benzyl-4′,6′-O-benzylidene-6-O-methyl-α,α-D-trehalose (16-a)*

General procedure A was applied (use of iodomethane). Purification via column chromatography (gradient elution: hexane/EtOAc 9/1 to 8/2) afforded the title compound **16-a** as a white solid (203 mg, 0.227 mmol, 80%).

Rf 0.27 in hexane/EtOAc 7/3. $^1H$ NMR (400 MHz, benzene-$d_6$): $\delta$ 7.64–7.56 (m, 2H), 7.43–7.26 (m, 10H), 7.24–7.02 (m, 18H), 5.41 (d, $J = 3.8$ Hz, 1H), 5.39 (s, 1H), 5.33 (d, $J = 3.7$ Hz, 1H), 5.00–4.90 (m, 3H), 4.84–4.63 (m, 5H), 4.58–4.46 (m, 4H), 4.41 (app t, $J = 9.4$ Hz, 1H), 4.36 (app t, $J = 9.7$ Hz, 1H), 4.19 (dd, $J = 10.1/4.9$ Hz, 1H), 3.80 (t, $J = 10.1/9.2$ Hz, 1H), 3.67–3.48 (m, 6H), 3.17 (s, 3H) ppm. $^{13}C$ NMR (100 MHz, benzene-$d_6$): $\delta$ 140.2 (C), 140.1 (C), 139.8 (C), 139.3 (C), 139.0 (C), 138.8 (C), 129.3 (CH), 129.1 (CH), 129.0 (CH), 128.9 (CH), 128.8 (CH), 128.7 (CH), 128.3 (CH), 128.2 (CH), 128.2 (CH), 128.0 (CH), 127.8 (CH), 127.1 (CH), 102.0 (CH), 94.8 (CH), 94.0 (CH), 83.4 (CH), 82.7 (CH), 80.7 (CH), 79.7 (CH), 79.7 (CH), 78.9 (CH), 75.8 ($CH_2$), 75.6 ($CH_2$), 75.3 ($CH_2$), 74.2 ($CH_2$), 74.0 ($CH_2$), 72.2 ($CH_2$), 72.1 (CH), 69.7 (CH), 63.8 (CH), 59.4 (CH) ppm. IR (HATR): 3064 (w), 3029 (w), 2923 (w), 2868 (w), 1496 (w), 1453 (m), 1367 (m), 1330 (w), 1208 (w), 1149 (m), 1136 (m), 1085 (s), 1071 (s), 1049 (m), 1027 (m), 985 (s), 915 (w), 854 (w), 797 (w), 734 (m), 695 (s), 658 (w), 633 (w) $cm^{-1}$. ESMS [m/z (fragment, intensity), API-ES positive mode]: 912.3 ($M+NH_4^+$, 100). HRMS (ESI-TOF): calcd. for $C_{55}H_{62}NO_{11}^+$ $[M+NH_4]^+$ 912.4317; found 912.4343.

### *2,3,4,2′,3′-Penta-O-benzyl-4′,6′-O-benzylidene-6-O-ethyl-α,α-D-trehalose (16-b)*

General procedure A was applied (use of iodoethane). Purification via column chromatography (gradient elution: hexane/EtOAc 9/1 to 8/2) afforded the title compound as a white solid (476 mg, 0.524 mmol, 92%).

Rf 0.32 in hexane/EtOAc 7/3. $^1H$ NMR (400 MHz, benzene-$d_6$): $\delta$ 7.64–7.56 (m, 2H), 7.42–7.27 (m, 10H), 7.23–7.02 (m, 18H), 5.42 (d, $J = 3.7$ Hz, 1H), 5.38 (s, 1H), 5.33 (d, $J = 3.5$ Hz, 1H), 5.02–4.91 (m, 3H), 4.84–4.66 (m, 5H), 4.58–4.46 (m, 4H), 4.40 (t, $J = 9.2$ Hz, 1H), 4.36 (t, $J = 9.2$ Hz, 1H), 4.18 (dd, $J = 10.2/4.9$ Hz, 1H), 3.86 (t, $J = 9.5$ Hz, 1H), 3.68–3.49 (m, 6H), 3.45–3.15 (m, 1H), 3.35–3.24 (m, 1H), 1.11 (t, $J = 7.0$ Hz, 3H) ppm. $^{13}C$ NMR (100 MHz, benzene-$d_6$): $\delta$ 139.8 (C), 139.7 (C), 139.5 (C), 138.9 (C), 138.6 (C), 138.5 (C), 128.9 (CH), 128.7 (CH), 128.6 (CH), 128.4 (CH), 128.4 (CH), 128.3 (CH), 128.0 (CH), 127.9 (CH), 127.8 (CH), 127.6 (CH), 127.4 (CH), 126.8 (CH), 101.7 (CH), 94.4 (CH), 93.7 (CH), 83.0 (CH), 82.4 (CH), 80.3 (CH), 79.3 (CH), 78.5 (CH), 75.5 ($CH_2$), 75.2 ($CH_2$), 74.9 ($CH_2$),

73.8 ($CH_2$), 73.6 ($CH_2$), 71.7 (CH), 69.7 ($CH_2$), 69.3 ($CH_2$), 66.9 ($CH_2$), 63.4 (CH), 15.5 ($CH_3$) ppm. IR (HATR): 3064 (w), 3029 (w), 2973 (w), 2914 (w), 2862 (w), 1496 (w), 1454 (m), 1368 (m), 1328 (w), 1209 (w), 1135 (m), 1086 (s), 1071 (s), 984 (s), 915 (m), 797 (w), 734 (m), 695 (s), 659 (w) $cm^{-1}$. ESMS [m/z (fragment, intensity), API-ES positive mode]: 926.4 ($M+NH_4^+$, 100). HRMS (ESI-TOF): calcd. for $C_{56}H_{64}NO_{11}^+$ $[M+NH_4]^+$ 926.4474; found 926.4485.

### *6-O-Allyl-2,3,4,2′,3′-penta-O-benzyl-4′,6′-O-benzylidene-α,α-D-trehalose (16-c)*

General procedure A was applied (use of allyl bromide). Purification via column chromatography (gradient elution: hexane/EtOAc 9/1 to 8/2) afforded the title compound **16-c** as a white solid (460 mg, 0.499 mmol, 88%).

Rf 0.34 in hexane/EtOAc 7/3. $^1H$ NMR (400 MHz, benzene-$d_6$): $\delta$ 7.62–7.57 (m, 2H), 7.41–7.26 (m, 10H), 7.23–7.02 (m, 18H), 5.84 (app ddt, $J=17.2/10.4/5.3$ Hz, 1H), 5.41 (d, $J=3.8$ Hz, 1H), 5.38 (s, 1H), 5.33 (d, $J=3.5$ Hz, 1H), 5.25 (app dq, $J=17.3/1.8$ Hz, 1H), 5.04 (app dq, $J=10.4/1.5$ Hz, 1H), 5.00–4.91 (m, 3H), 4.83–4.65 (m, 5H), 4.58–4.47 (m, 4H), 4.40 (app t, $J=9.2$ Hz, 1H), 4.36 (app t, $J=9.3$ Hz, 1H), 4.19 (dd, $J=10.1/4.9$ Hz, 1H), 3.91 (app ddt, $J=13.1/5.3/1.5$ Hz, 1H), 3.88–3.80 (m, 2H), 3.68–3.49 (m, 6H) ppm. $^{13}C$ NMR (100 MHz, benzene-$d_6$): $\delta$ 139.8 (C), 139.7 (C), 139.4 (C), 138.9 (C), 138.6 (C), 138.5 (C), 135.5 (CH), 128.9 (CH), 128.7 (CH), 128.6 (CH), 128.4 (CH), 128.4 (CH), 128.3 (CH), 127.9 (CH), 127.8 (CH), 127.6 (CH), 127.4 (CH), 126.8 (CH), 116.3 ($CH_2$), 101.7 (CH), 94.4 (CH), 93.6 (CH), 83.0 (CH), 82.4 (CH), 80.3 (CH), 79.3 (CH), 79.3 (CH), 78.5 (CH), 75.5 ($CH_2$), 75.2 ($CH_2$), 75.0 ($CH_2$), 73.9 ($CH_2$), 73.6 ($CH_2$), 72.5 ($CH_2$), 71.7 (CH), 69.4 ($CH_2$), 63.4 (CH) ppm. IR (HATR): 3064 (w), 3029 (w), 2923 (w), 2864 (w), 1496 (w), 1453 (m), 1366 (m), 1331 (w), 1209 (w), 1151 (m), 1135 (m), 1086 (s), 1071 (s), 985 (s), 928 (m), 734 (m), 695 (s), 656 (m) $cm^{-1}$. ESMS [m/z (fragment, intensity), API-ES positive mode]: 938.4 ($M+NH_4^+$, 100). HRMS (ESI-TOF): calcd. for $C_{57}H_{64}NO_{11}^+$ $[M+NH_4]^+$ 938.4474; found 938.4509.

### *2,3,4,2′,3′-Penta-O-benzyl-4′,6′-O-benzylidene-6-O-n-butyl-α,α-D-trehalose (16-d)*

General procedure A was applied (use of 1-iodobutane). Purification via column chromatography (gradient elution: hexane/EtOAc 9/1 to 7/3) afforded the title compound **16-d** as a white solid (445 mg, 0.475 mmol, 84%).

Rf 0.54 in hexane/EtOAc 6/4. $^1H$ NMR (400 MHz, benzene-$d_6$): $\delta$ 7.64–7.56 (m, 2H), 7.43–7.27 (m, 10H), 7.23–7.02 (m, 18H), 5.43 (d, $J=3.8$ Hz, 1H), 5.38 (s, 1H), 5.34 (d, $J=3.5$ Hz, 1H), 5.02 (d, $J=11.4$ Hz, 1H), 4.95 (d, $J=11.6$ Hz, 1H), 4.94 (d, $J=11.4$ Hz, 1H), 4.84–4.66 (m, 5H), 4.58–4.46 (m, 4H), 4.45–4.33 (m, 2H), 4.20 (dd, $J=10.1/4.9$ Hz, 1H), 3.95–3.85 (m, 1H), 3.72–3.48 (m, 6H), 3.46–3.38 (m, 1H), 3.35–3.26 (m, 1H), 1.63–1.27 (m, 4H), 0.85 (t, $J=7.3$ Hz, 3H) ppm. $^{13}C$ NMR (100 MHz, benzene-$d_6$): $\delta$ 139.8 (C), 139.7 (C), 139.5 (C), 138.9 (C), 138.6 (C), 138.5 (C), 128.9 (CH), 128.7 (CH), 128.6 (CH), 128.5 (CH), 128.4 (CH), 128.3 (CH), 127.9 (CH), 127.9 (CH), 127.9 (CH), 127.6 (CH), 127.4 (CH), 126.8 (CH), 101.7 (CH), 94.4 (CH), 93.7 (CH), 83.0 (CH), 82.4 (CH), 80.4 (CH), 79.4 (CH), 79.3 (CH), 78.6 (CH), 75.5 ($CH_2$), 75.2 ($CH_2$), 75.0 ($CH_2$), 73.9 ($CH_2$), 73.6 ($CH_2$), 71.7 (CH), 71.5 ($CH_2$), 70.0 ($CH_2$), 69.3 ($CH_2$), 63.4 (CH), 32.3 ($CH_2$), 19.8 ($CH_2$), 14.1 ($CH_3$) ppm. IR (HATR): 3064 (w), 3027 (w), 2932 (w), 2862 (w), 1496 (w), 1454 (m), 1367 (m), 1328 (w), 1210 (w), 1154 (m), 1086 (s), 1072 (s), 985 (s), 915 (m), 733 (m), 695 (s), 657 (w) $cm^{-1}$. ESMS [m/z (fragment, intensity), API-ES positive mode]: 954.4 ($M+NH_4^+$, 100). HRMS (ESI-TOF): calcd. for $C_{58}H_{68}NO_{11}^+$ $[M+NH_4]^+$ 954.4787; found 954.4778.

### *2,3,4,2′,3′-Penta-O-benzyl-4′,6′-O-benzylidene-6-O-(2-methylallyl)-α,α-D-trehalose (16-e)*

General procedure A was applied (use of 3-bromo-2-methylpropene, "methallyl bromide"). Purification via column chromatography (gradient elution: hexane/EtOAc 9/1 to 8/2) afforded the title compound **16-e** as a white solid (247 mg, 0.264 mmol, 93%).

Rf 0.64 in hexane/EtOAc 1/1. $^1H$ NMR (400 MHz, benzene-$d_6$): $\delta$ 7.63–7.57 (m, 2H), 7.45–7.26 (m, 10H), 7.24–7.02 (m, 18H), 5.41 (d, $J=3.7$ Hz, 1H), 5.38 (s, 1H), 5.33 (d, $J=3.5$ Hz, 1H), 5.11–5.05 (m, 1H), 5.03–4.90 (m, 3H), 4.89–4.84 (m, 1H), 4.57–4.46 (m, 4H), 4.40 (app t, $J=9.3$ Hz, 1H), 4.36 (app t, $J=9.3$ Hz, 1H), 4.19 (dd, $J=10.1/4.9$ Hz, 1H), 3.91–3.74 (m, 3H), 3.70–3.47 (m, 6H), 1.67 (s, 3H) ppm. $^{13}C$ NMR (100 MHz, benzene-$d_6$): $\delta$ 142.7 (C), 139.7 (C), 139.7 (C), 139.4 (C), 138.9 (C), 138.6 (C), 138.5 (C), 128.9 (CH), 128.7 (CH), 128.6 (CH), 128.4 (CH), 128.4 (CH), 128.3 (CH), 128.0 (CH), 127.9 (CH), 127.9 (CH), 127.8 (CH), 127.6 (CH), 127.4 (CH), 126.7 (CH), 112.0 ($CH_2$), 101.6 (CH), 94.3 (CH), 93.6 (CH), 83.0 (CH), 82.4 (CH), 80.4 (CH), 79.3 (CH), 79.3 (CH), 78.6 (CH), 75.5 ($CH_2$), 75.5 ($CH_2$), 75.2 ($CH_2$), 75.0 ($CH_2$), 73.9 ($CH_2$), 73.6 ($CH_2$), 71.7 (CH), 69.3 ($CH_2$), 63.4 (CH), 19.6 ($CH_3$) ppm. IR (HATR): 3088 (w), 3060 (w), 3030 (w), 2925 (w), 2861 (w), 1496 (w), 1453 (w), 1388 (w), 1367 (w), 1329 (w), 1279 (w), 1237 (w), 1209 (w), 1153 (m), 1136 (m), 1086 (s), 1072 (s), 1025 (s), 986 (s), 908 (w), 877 (w), 846 (w), 796 (w), 745 (m), 734 (m), 695 (s), 656 (w), 634 (w) $cm^{-1}$. ESMS [m/z (fragment, intensity), API-ES positive mode]: 952.4 ($M+NH_4^+$, 100). HRMS (ESI-TOF): calcd. for $C_{58}H_{66}NO_{11}^+$ $[M+H]^+$ 952.4630; found 952.4641.

### *2,3,4,2′,3′-Penta-O-benzyl-4′,6′-O-benzylidene-6-O-(3,3-dimethyallyl)-α,α-D-trehalose (16-f)*

General procedure A was applied (use of 1-bromo-3-methyl-2-butene, "prenyl bromide"). Purification via column chromatography (hexane/EtOAc 8/2) afforded the partially purified title compound **16-f** as a white solid which was used as such in the next step.

Rf 0.58 in hexane/EtOAc 6/4.

### *2,3,4,2′,3′-Penta-O-benzyl-4′,6′-O-benzylidene-6-O-n-decyl-α,α-D-trehalose (16-g)*

General procedure A was applied (use of 1-bromodecane). Purification via column chromatography (gradient elution: hexane/EtOAc 9/1 to 8/2) afforded the partially purified title compound **16-g** as a white solid (320 mg), which was used as such in the next step.

Rf 0.67 in hexane/EtOAc 1/1. ESMS [m/z (fragment, intensity), API-ES positive mode]: 1038.5 ($M+NH_4^+$, 100). HRMS (ESI-TOF): calcd. for $C_{64}H_{80}NO_{11}^+$ $[M+NH_4]^+$ 1038.5726; found 1038.5742.

### *6-O-Methyl-α,α-D-trehalose (17-a)*

General procedure C was applied. Purification via column chromatography (gradient elution: $CH_2Cl_2$/MeOH 8/2 to 6/4) afforded the title compound **17-a** as a white solid (77.9 mg, 0.219 mmol, 96%).

Rf 0.35 in $CH_2Cl_2$/MeOH 6/4. $^1H$ NMR (400 MHz, $CD_3OD$): $\delta$ 5.09 (d, $J=3.4$ Hz, 1H), 5.08 (d, $J=3.5$ Hz, 1H), 3.94 (ddd, $J=10.0/4.9/2.6$ Hz, 1H), 3.85–3.73 (m, 3H), 3.70–3.53 (m, 5H), 3.48 (d, $J=3.8$ Hz, 1H), 3.45 (d, $J=3.8$ Hz, 1H), 3.37 (s, 3H), 3.35–3.27 (m, 2H) ppm. $^{13}C$ NMR (100 MHz, $CD_3OD$): $\delta$ 95.2 (CH), 74.6 (CH), 74.5 (CH), 73.8

(CH), 73.2 (CH), 73.1 (CH), 73.1 ($CH_2$), 72.4 (CH), 72.0 (CH), 71.9 (CH), 62.6 ($CH_2$), 59.5 ($CH_3$) ppm. IR (HATR): 3292 (br, m), 2927 (w), 1652 (w), 1637 (w), 1455 (w), 1438 (w), 1419 (w), 1363 (w), 1337 (w), 1265 (w), 1197 (w), 1146 (m), 1100 (m), 1079 (m), 1032 (m), 986 (s), 943 (m), 912 (w), 852 (w), 805 (w), 705 (w), 667 (w), 608 (w) $cm^{-1}$. $[\alpha]_D^{22}$ +198° (c 0.12, MeOH). ESMS [m/z (fragment, intensity), API-ES positive mode]: 374.1 ($M+NH_4^+$, 100). HRMS (ESI-TOF): calcd. for $C_{13}H_{28}NO_{11}^+$ $[M+NH_4]^+$ 374.1657; found 374.1661.

### 6-O-Ethyl-α,α-D-trehalose (17-b)

General procedure C was applied. Purification via column chromatography (gradient elution: $CH_2Cl_2$/MeOH 8/2 to 6/4) afforded the title compound **17-b** as a white solid (71.9 mg, 0.194 mmol, 98%).

Rf 0.29 in $CH_3CN/H_2O$ 8/2, 0.41 in $CH_2Cl_2$/MeOH 6/4. $^1H$ NMR (400 MHz, $CD_3OD$): $\delta$ 5.09 (d, $J=3.4$ Hz, 1H), 5.08 (d, $J=3.5$ Hz, 1H), 3.92 (ddd, $J=9.9/5.2/2.1$ Hz, 1H), 3.85–3.72 (m, 4H), 3.72–3.41 (m, 7H), 3.38–3.28 (m, 2H), 1.18 (t, $J=7.0$ Hz, 3H) ppm. $^{13}C$ NMR (100 MHz, $CD_3OD$): $\delta$ 95.1 (CH), 95.0 (CH), 74.6 (CH), 74.5 (CH), 73.8 (CH), 73.2 (CH), 73.0 (CH), 72.5 (CH), 72.1 (CH), 71.9 (CH), 70.9 ($CH_2$), 67.9 ($CH_2$), 62.6 ($CH_2$), 15.4 ($CH_3$) ppm. IR (HATR): 3305 (br, m), 2976 (w), 2926 (w), 1652 (w), 1455 (w), 1418 (w), 1375 (w), 1360 (w), 1335 (w), 1271 (w), 1206 (w), 1146 (m), 1100 (m), 1075 (m), 1028 (m), 985 (s), 941 (m), 913 (w), 853 (w), 804 (w), 734 (w), 702 (w), 642 (w), 611 (w) $cm^{-1}$. $[\alpha]_D^{22}$ +213° (c 0.14, MeOH). ESMS [m/z (fragment, intensity), API-ES positive mode]: 388.0 ($M+NH_4^+$, 100). HRMS (ESI-TOF): calcd. for $C_{14}H_{30}NO_{11}^+$ $[M+NH_4]^+$ 388.1813; found 388.1827.

### 6-O-n-Propyl-α,α-D-trehalose (17-c)

General procedure C was applied. Purification via column chromatography (gradient elution: $CH_2Cl_2$/MeOH 9/1 to 65/35) afforded the title compound as a white solid (125 mg, 0.338 mmol, 71%).

Rf 0.34 in $CH_3CN/H_2O$ 8/2, 0.27 in $CH_2Cl_2$/MeOH 7/3. $^1H$ NMR (400 MHz, $CD_3OD$): $\delta$ 5.09 (d, $J=3.3$ Hz, 1H), 5.09 (d, $J=3.5$ Hz, 1H), 3.91 (ddd, $J=10.0/5.2/2.1$ Hz, 1H), 3.87–3.73 (m, 4H), 3.71–3.56 (m, 3H), 3.54–3.37 (m, 4H), 3.37–3.27 (m, 2H), 1.58 (app hexaplet, $J=7.1$ Hz, 2H), 0.92 (t, $J=7.4$ Hz, 3H), ppm. $^{13}C$ NMR (100 MHz, $CD_3OD$): $\delta$ 95.1 (CH), 95.0 (CH), 74.6 (CH), 74.5 (CH), 74.3 ($CH_2$), 73.8 (CH), 73.2 (CH), 73.2 (CH), 72.6 (CH), 72.1 (CH), 71.9 (CH), 71.1 ($CH_2$), 62.6 ($CH_2$), 23.8 ($CH_2$), 10.8 ($CH_3$) ppm. IR (HATR): 3312 (m, br), 2928 (w), 2872 (w), 1435 (w), 1372 (w), 1334 (w), 1266 (w), 1204 (w), 1146 (m), 1101 (m), 1075 (m), 1033 (m), 987 (s), 943 (m), 912 (w), 853 (w), 845 (w), 804 (w), 700 (w) $cm^{-1}$. $[\alpha]_D^{22}$ +231° (c 0.10, MeOH). ESMS [m/z (fragment, intensity), API-ES negative mode]: 402.2 ($M+NH_4^+$, 100). HRMS (ESI-TOF): calcd. for $C_{15}H_{28}NaO_{11}$ $[M+Na]^+$ 407.1524; found 407.1513.

### 6-O-n-Butyl-α,α-D-trehalose (17-d)

General procedure C was applied. Purification via column chromatography ($CH_2Cl_2$/MeOH 7/3) afforded the title compound **17-d** as a white solid (145 mg, 0.364 mmol, 81%).

Rf 0.21 in $CH_3CN/H_2O$ 8/2, 0.24 in $CH_2Cl_2$/MeOH 7/3. $^1H$ NMR (400 MHz, $CD_3OD$): $\delta$ 5.13–5.06 (m, 2H), 3.92 (ddd, $J=9.9/4.9/2.1$ Hz, 1H), 3.86–3.72 (m, 4H), 3.71–3.41 (m, 7H), 3.37–3.27 (m, 2H), 1.62–1.48 (m, 2H), 1.38 (app hexaplet, $J=7.5$ Hz, 2H), 0.92 (t, $J=7.4$ Hz, 3H) ppm. $^{13}C$ NMR (100 MHz, $CD_3OD$): $\delta$ 95.1 (CH), 95.0 (CH), 74.6 (CH), 74.5 (CH), 73.8 (CH), 73.2 (CH), 73.2 (CH), 72.6 (CH), 72.4 (CH2), 72.1 (CH), 71.9 (CH), 71.2 ($CH_2$), 62.6 ($CH_2$), 32.8 ($CH_2$), 20.3 ($CH_2$), 14.3 (CH) ppm. IR (HATR): 3301 (m, br), 2929 (w), 2872 (w), 1644 (w), 1455 (w), 1430 (w), 1372 (w), 1334 (w), 1263 (w), 1146 (m), 1101 (m), 1077 (m), 1029 (m), 985 (s), 941 (m), 912 (w), 844 (w), 805 (w), 706 (w) $cm^{-1}$. $[\alpha]_D^{22}$ +271° (c 0.12, MeOH). ESMS [m/z (fragment, intensity), API-ES positive mode]: 416.2 ($M+NH_4^+$, 100). HRMS (ESI-TOF): calcd. for $C_{16}H_{30}NaO_{11}^+$ $[M+Na]^+$ 421.1680; found 421.1676.

### 6-O-Isobutyl-α,α-D-trehalose (17-e)

General procedure C was applied. Purification via column chromatography (gradient elution: $CH_2Cl_2$/MeOH 8/2 to 6/4) afforded the title compound as a white solid (71.0 mg, 0.178 mmol, 79%).

Rf 0.32 in $CH_2Cl_2$/MeOH 6/4. $^1H$ NMR (400 MHz, $CD_3OD$): $\delta$ 5.09 (app d, $J=3.7$ Hz, 2H), 3.92 (ddd, $J=10.0/5.0/2.4$ Hz, 1H), 3.84–3.74 (m, 4H), 3.70–3.58 (m, 3H), 3.48 (app t, $J=3.8$ Hz, 1H), 3.45 (app t, $J=3.8$ Hz, 1H), 3.37–3.27 (m, 3H), 3.21 (dd, $J=9.3/6.8$ Hz, 1H), 1.85 (nonaplet, $J=6.7$ Hz, 1H), 0.90 (d, $J=6.7$ Hz, 3H), 0.90 (d, $J=6.7$ Hz, 3H) ppm. $^{13}C$ NMR (100 MHz, $CD_3OD$): $\delta$ 95.0 (CH), 94.9 (CH), 79.5 ($CH_2$), 74.7 (CH), 74.6 (CH), 73.8 (CH), 73.2 (CH), 73.2 (CH), 72.6 (CH), 72.1 (CH), 71.9 (CH), 71.3 ($CH_2$), 62.6 ($CH_2$), 29.5 (CH), 19.7 ($CH_3$) ppm. IR (HATR): 3306 (br, m), 2953 (w), 2928 (w), 2875 (w), 1652 (w), 1637 (w), 1456 (w), 1436 (w), 1419 (w), 1388 (w), 1365 (w), 1335 (w), 1270 (w), 1209 (w), 1147 (m), 1102 (m), 1077 (m), 1031 (m), 985 (s), 941 (m), 913 (w), 846 (w), 804 (w), 706 (w), 642 (w), 612 (w) $cm^{-1}$. $[\alpha]_D^{22}$ +165° (c 0.10, MeOH). ESMS [m/z (fragment, intensity), API-ES negative mode]: 416.2 ($M+NH_4^+$, 100). HRMS (ESI-TOF): calcd. for $C_{16}H_{34}NO_{11}^+$ $[M+NH_4]^+$ 416.2126; found 416.2138.

### 6-O-Isopentyl-α,α-D-trehalose (17-f)

General procedure C was applied. Purification via column chromatography (gradient elution: $CH_2Cl_2$/MeOH 9/1 to 7/3) afforded the title compound **17-f** as a white solid (40 mg, 0.097 mmol, 17% over 2 steps).

Rf 0.29 in $CH_3CN/H_2O$ 8/2, 0.49 in $CH_2Cl_2$/MeOH 6/4. $^1H$ NMR (400 MHz, $CD_3OD$): $\delta$ 5.10 (d, $J=3.3$ Hz, 1H), 5.09 (d, $J=3.3$ Hz, 1H), 3.91 (ddd, $J=9.9/4.9/2.2$ Hz, 1H), 3.85–3.73 (m, 4H), 3.71–3.41 (m, 7H), 3.38–3.26 (m, 2H), 1.70 (heptaplet, $J=6.7$ Hz, 1H), 1.46 (q, $J=6.8$ Hz, 2H), 0.91 (d, $J=6.6$ Hz, 6H) ppm. $^{13}C$ NMR (100 MHz, $CD_3OD$): $\delta$ 95.1 (CH), 95.0 (CH), 74.6 (CH), 74.5 (CH), 73.8 (CH), 73.2 (CH), 73.1 (CH), 72.6 (CH), 72.1 (CH), 71.9 (CH), 71.2 ($CH_2$), 71.1 ($CH_2$), 62.6 ($CH_2$), 39.6 ($CH_2$), 26.2 (CH), 23.1 ($CH_3$), 23.0 ($CH_3$) ppm. IR (HATR): 3308 (m, br), 2951 (w), 2926 (w), 2867 (w), 1455 (w), 1423 (w), 1366 (w), 1337 (w), 1266 (w), 1204 (w), 1145 (m), 1102 (m), 1076 (m), 1032 (m), 987 (s), 942 (m), 914 (w), 847 (w), 806 (w) $cm^{-1}$. $[\alpha]_D^{22}$ +183° (c 0.06, MeOH). ESMS [m/z (fragment, intensity), API-ES negative mode]: 430.2 ($M+NH_4^+$, 100). HRMS (ESI-TOF): calcd. for $C_{17}H_{32}NaO_{11}$ $[M+Na]^+$ 435.1837; found 435.1833.

### 6-O-n-Decyl-α,α-D-trehalose (17-g)

General procedure C was applied. Purification via column chromatography (gradient elution: $CH_2Cl_2$/MeOH 8/2 to 6/4) afforded the title compound **17-g** as a white solid (88.0 mg, 0.182 mmol, 64% over two steps).

Rf 0.61 in $CH_2Cl_2$/MeOH 6/4. $^1H$ NMR (400 MHz, $CD_3OD$): $\delta$ 5.10 (d, $J=3.2$ Hz, 1H), 5.09 (d, $J=3.3$ Hz, 1H), 3.92 (ddd, $J=10.0/5.1/2.2$ Hz, 1H), 3.85–3.74 (m, 4H), 3.70–3.58 (m, 3H), 3.56–3.41 (m, 4H), 3.37–3.29 (m, 1H), 1.56 (quintet, $J=6.9$ Hz, 2H), 1.42–1.20 (m, 14H), 0.89 (t, $J=6.9$ Hz, 3H) ppm. $^{13}C$ NMR (100 MHz, $CD_3OD$): $\delta$ 95.0 (CH), 94.9 (CH), 79.5 ($CH_2$), 74.6 (CH), 74.5 (CH), 73.8 (CH), 73.2 (CH), 73.1 (CH), 72.8 (CH), 72.6 (CH), 72.1 (CH), 71.9 (CH), 71.1 ($CH_2$), 62.6 ($CH_2$), 33.1 ($CH_2$), 30.7 ($CH_2$), 30.7 ($CH_2$), 30.6 ($CH_2$), 30.5 ($CH_2$), 27.2 ($CH_2$), 23.7 ($CH_2$), 14.4 ($CH_3$) ppm. IR (HATR): 3310 (m, br), 2923 (m), 2854 (m), 1456 (w), 1428 (w), 1377 (w), 1337 (w), 1275 (w), 1202 (w), 1146 (m), 1104 (m), 1078 (m), 1034 (m), 987 (s), 941 (m), 912 (w), 848 (w), 805 (w), 720 (w), 708 (w) $cm^{-1}$. $[\alpha]_D^{22}$ +182° (c 0.10, MeOH). ESMS [m/z (fragment, intensity), API-ES positive mode]: 500.3 ($M+NH_4^+$, 100). HRMS (ESI-TOF): calcd. for $C_{22}H_{42}NaO_{11}^+$ $[M+Na]^+$ 505.2619; found 505.2616.

### *2,3,4,2′,3′-Penta-O-benzyl-4′,6′-O-benzylidene-6-O-(N-n-propylcarbamoyl)-α,α-D-trehalose (18-a)*

General procedure B was applied (use of *n*-propyl isocyanate). Purification via column chromatography (gradient elution: hexane/EtOAc 8/2 to 7/3) afforded the title compound **18-a** as a white foam (187 mg, 0.194 mmol, 85%).

Rf 0.55 in hexane/EtOAc 1/1. $^1$H NMR (400 MHz, benzene-$d_6$): δ 7.63–7.00 (m, 30H), 5.38 (s, 1H), 5.34 (d, *J* = 4.0 Hz, 1H), 5.31 (d, *J* = 3.3 Hz, 1H), 5.03–4.90 (m, 3H), 4.81–4.03 (m, 16H), 3.79–3.44 (m, 5H), 3.12 (s, 1H), 2.88 (q, *J* = 6.6 Hz, 2H), 1.12 (app sextet, *J* = 7.3 Hz, 2H), 0.59 (t, *J* = 7.4 Hz, 3H) ppm. $^{13}$C NMR (100 MHz, benzene-$d_6$): δ 156.2 (C), 139.6 (C), 138.9 (C), 138.7 (C), 138.43 (C), 138.4 (C), 128.9 (CH), 128.8 (CH), 128.7 (CH), 128.5 (CH), 128.5 (CH), 128.4 (CH), 128.4 (CH), 128.3 (CH), 127.9 (CH), 127.9 (CH), 127.8 (CH), 127.5 (CH), 126.7 (CH), 102.8 (CH), 101.7 (CH), 94.6 (CH), 93.6 (CH), 82.9 (CH), 82.3 (CH), 80.1 (CH), 79.4 (CH), 79.2 (CH), 78.2 (CH), 75.5 ($CH_2$), 75.3 ($CH_2$), 75.0 ($CH_2$), 74.1 ($CH_2$), 73.5 ($CH_2$), 70.3 (CH), 69.3 ($CH_2$), 63.5 ($CH_2$), 63.5 ($CH_2$), 51.9 (CH), 42.9 ($CH_2$), 23.3 ($CH_2$), 11.1 ($CH_3$) ppm. IR (HATR): 3060 (w), 3031 (w), 2932 (w), 2869 (w), 1722 (m), 1517 (w), 1496 (w), 1454 (m), 1388 (w), 1368 (s), 1330 (w), 1279 (w), 1259 (w), 1234 (w), 1212 (w), 1153 (m), 1134 (m), 1085 (s), 1072 (s), 1005 (s), 981 (s), 930 (m), 874 (w), 846 (w), 798 (w), 747 (m), 734 (m), 695 (s), 654 (w), 631 (w) $cm^{-1}$. ESMS [m/z (fragment, intensity), API-ES positive mode]: 983.4 ($M+NH_4^+$, 100). HRMS (ESI-TOF): calcd. for $C_{58}H_{67}N_2O_{12}^+$ $[M+NH_4]^+$ 983.4689; found 983.4661.

### *2,3,4,2′,3′-Penta-O-benzyl-4′,6′-O-benzylidene-6-O-(N-isopropylcarbamoyl)-α,α-D-trehalose (18-b)*

General procedure B was applied (use of isopropyl isocyanate). Purification via column chromatography (hexane/EtOAc 8/2) afforded the title compound **18-b** as a white foam (174 mg, 0.180 mmol, 79%).

Rf 0.64 in hexane/EtOAc 1/1. $^1$H NMR (400 MHz, benzene-$d_6$): δ 7.63–7.58 (m, 2H), 7.38–7.27 (m, 10H), 7.25–6.92 (m, 38H), 5.38 (s, 1H), 5.35 (d, *J* = 3.8 Hz, 1H), 5.32 (d, *J* = 3.5 Hz, 1H), 5.00–4.92 (m, 3H), 4.80–4.62 (m, 4H), 4.62–4.31 (m, 9H), 4.19 (dd, *J* = 10.1/4.9 Hz, 1H), 4.11–4.00 (m, 1H), 3.81–3.43 (m, 6H), 0.79 (d, *J* = 6.6 Hz, 3H), 0.76 (d, *J* = 6.6 Hz, 3H) ppm. $^{13}$C NMR (100 MHz, benzene-$d_6$): δ 153.4 (C), 139.6 (C), 138.9 (C), 138.7 (C), 138.4 (C), 128.9 (CH), 128.8 (CH), 128.8 (CH), 128.5 (CH), 128.5 (CH), 128.4 (CH), 128.4 (CH), 128.3 (CH), 128.0 (CH), 127.9 (CH), 127.8 (CH), 127.5 (CH), 127.5 (CH), 126.7 (CH), 101.6 (CH), 94.6 (CH), 93.6 (CH), 82.9 (CH), 82.3 (CH), 80.2 (CH), 79.4 (CH), 79.2 (CH), 78.2 (CH), 75.5 ($CH_2$), 75.2 ($CH_2$), 75.0 ($CH_2$), 74.1 ($CH_2$), 73.5 ($CH_2$), 70.3 (CH), 69.3 ($CH_2$), 63.5 (CH), 63.4 ($CH_2$), 43.0 (CH), 22.7 ($CH_3$), 22.6 ($CH_3$) ppm. IR (HATR): 3060 (w), 3031 (w), 2970 (w), 2932 (w), 2868 (w), 1718 (m), 1505 (w), 1497 (w), 1454 (m), 1385 (w), 1367 (w), 1321 (w), 1234 (w), 1212 (w), 1153 (w), 1134 (m), 1086 (s), 1071 (s), 1025 (m), 984 (s), 913 (w), 874 (m), 846 (w), 798 (w), 748 (m), 734 (m), 695 (s), 656 (w), 631 (w) $cm^{-1}$. ESMS [m/z (fragment, intensity), API-ES positive mode]: 983.4 ($M+NH_4^+$, 100). HRMS (ESI-TOF): calcd. for $C_{58}H_{67}N_2O_{12}^+$ $[M+NH_4]^+$ 983.4689; found 983.4666.

### *2,3,4,2′,3′-Penta-O-benzyl-4′,6′-O-benzylidene-6-O-(N-cyclohexylcarbamoyl)-α,α-D-trehalose (18-c)*

General procedure B was applied (use of cyclohexyl isocyanate). Purification via column chromatography (gradient elution: hexane/EtOAc 8/2 to 7/3) afforded the title compound **18-c** as a white foam (411 mg, 0.408 mmol, 81%).

Rf 0.29 in hexane/EtOAc 6/4. $^1$H NMR (400 MHz, benzene-$d_6$): δ 7.63–7.58 (m, 2H), 7.39–7.28 (m, 10H), 7.23–7.02 (m, 18H), 5.39 (s, 1H), 5.36 (d, *J* = 3.7 Hz,1H), 5.33 (d, *J* = 3.4 Hz, 1H), 5.01–4.92 (m, 3H), 4.79–4.69 (m, 4H), 4.66–4.45 (m, 7H), 4.40 (app t, *J* = 9.3 Hz, 1H), 4.37 (app t, *J* = 9.3 Hz, 1H), 4.26–4.18 (m, 1H), 4.20 (dd, *J* = 10.2/4.8 Hz, 1H), 3.75 (app br t, *J* = 9.2 Hz, 1H), 3.70–3.47 (m, 5H), 1.75 (app br t, *J* = 12.8 Hz, 2H), 1.44–1.24 (m, 3H), 1.10–0.70 (m, 5H) ppm. $^{13}$C NMR (100 MHz, benzene-$d_6$): δ 155.4 (C), 139.6 (C), 138.9 (C), 138.7 (C), 138.4 (C), 138.4 (C), 128.9 (CH), 128.8 (CH), 128.7 (CH), 128.5 (CH), 128.5 (CH), 128.4 (CH), 127.9 (CH), 127.8 (CH), 127.5 (CH), 127.5 (CH), 126.7 (CH), 101.7 (CH), 94.6 (CH), 93.6 (CH), 82.9 (CH), 82.3 (CH), 80.2 (CH), 79.4 (CH), 79.2 (CH), 78.2 (CH), 75.5 ($CH_2$), 75.3 ($CH_2$), 75.1 ($CH_2$), 74.1 ($CH_2$), 73.5 ($CH_2$), 70.4 (CH), 69.3 ($CH_2$), 63.5 (CH), 49.9 (CH), 33.4 ($CH_2$), 33.3 ($CH_2$), 25.7 ($CH_2$), 24.9 ($CH_2$) ppm. IR (HATR): 3032 (w), 2930 (w), 2854 (w), 1719 (m), 1496 (m), 1453 (m), 1365 (w), 1331 (w), 1315 (w), 1272 (w), 1251 (w), 1211 (m), 1151 (m), 1086 (s), 1072 (s), 985 (s), 735 (m), 696 (s), 658 (w) $cm^{-1}$. ESMS [m/z (fragment, intensity), API-ES positive mode]: 1023.4 ($M+NH_4^+$, 100). HRMS (ESI-TOF): calcd. for $C_{61}H_{71}N_2O_{12}^+$ $[M+NH_4]^+$ 1023.5002; found 1023.5013.

### *2,3,4,2′,3′-Penta-O-benzyl-4′,6′-O-benzylidene-6-O-(N-phenylcarbamoyl)-α,α-D-trehalose (18-d)*

General procedure B was applied (use of phenyl isocyanate). Purification via column chromatography (gradient elution: hexane/EtOAc 9/1 to 7/3) afforded the title compound **18-d** as a white foam (184 mg, 0.185 mmol, 81%).

Rf 0.25 in hexane/EtOAc 8/2. $^1$H NMR (400 MHz, benzene-$d_6$): δ 7.64–7.58 (m, 2H), 7.39–7.25 (m, 12H), 7.24–6.98 (m, 20H), 6.82 (tt, *J* = 7.4/1.1 Hz, 1H), 6.14 (s, 1H), 5.39 (s, 1H), 5.33 (d, *J* = 3.8 Hz, 1H), 5.31 (d, *J* = 3.6 Hz, 1H), 5.02–4.88 (m, 3H), 4.82–4.63 (m, 4H), 4.60–4.32 (m, 9H), 4.21 (dd, *J* = 10.1/4.9 Hz, 1H), 3.77–3.45 (m, 5H) ppm. $^{13}$C NMR (100 MHz, benzene-$d_6$): δ 153.1 (C), 139.6 (C), 139.5 (C), 138.8 (C), 138.7 (C), 138.6 (C), 138.4 (C), 138.4 (C), 129.1 (CH), 129.0 (CH), 128.8 (CH), 128.7 (CH), 128.5 (CH), 128.5 (CH), 128.5 (CH), 128.4 (CH), 128.3 (CH), 128.0 (CH), 128.0 (CH), 128.0 (CH), 127.9 (CH), 127.9 (CH), 127.8 (CH), 127.6 (CH), 127.5 (CH), 126.7 (CH), 123.3 (CH), 118.6 (CH), 101.7 (CH), 94.8 (CH), 93.7 (CH), 82.9 (CH), 82.3 (CH), 80.1 (CH), 79.4 (CH), 79.2 (CH), 77.6 (CH), 75.5 ($CH_2$), 75.3 ($CH_2$), 74.9 ($CH_2$), 74.2 ($CH_2$), 73.4 ($CH_2$), 70.0 (CH), 69.3 ($CH_2$), 63.8 ($CH_2$), 63.5 (CH) ppm. IR (HATR): 3391 (w), 3318 (w), 3060 (w), 3031 (w), 2931 (w), 2866 (w), 1732 (m), 1715 (m), 1601 (w), 1525 (m), 1497 (w), 1454 (m), 1444 (m), 1385 (w), 1367 (w), 1313 (w), 1210 (m), 1156 (w), 1137 (w), 1086 (s), 1071 (s), 985 (s), 912 (w), 877 (w), 846 (w), 798 (w), 748 (m), 734 (m), 694 (s), 656 (w) $cm^{-1}$. ESMS [m/z (fragment, intensity), API-ES positive mode]: 1017.3 ($M+NH^+$, 100). HRMS (ESI-TOF): calcd. for $C_{61}H_{65}N_2O_{12}^+$ $[M+NH_4]^+$ 1017.4532; found 1017.4524.

### *2,3,4,2′,3′-Penta-O-benzyl-4′,6′-O-benzylidene-6-O-(N-benzylcarbamoyl)-α,α-D-trehalose (18-e)*

General procedure B was applied (use of benzyl isocyanate). Purification via column chromatography (gradient elution: hexane/EtOAc 8/2 to 6/4) afforded the title compound **18-e** as a white foam (457 mg, 0.451 mmol, 79%).

Rf 0.27 in hexane/EtOAc 6/4. $^{1}H$ NMR (400 MHz, benzene-$d_6$): $\delta$ 7.64–7.57 (m, 2H), 7.39–7.27 (m, 10H), 7.24–6.94 (m, 23H), 5.39 (s, 1H), 5.33 (br d, $J=3.4$ Hz, 1H), 5.31 (br d, $J=2.9$ Hz, 1H), 5.02–4.87 (m, 3H), 4.80–4.30 (m, 14H), 4.21 (dd, $J=10.1/4.9$ Hz, 1H), 4.15–4.01 (m, 2H), 3.76–3.49 (m, 5H) ppm. $^{13}C$ NMR (100 MHz, benzene-$d_6$): $\delta$ 156.3 (C), 139.6 (C), 138.9 (C), 138.6 (C), 138.4 (C), 128.9 (CH), 128.8 (CH), 128.7 (CH), 128.6 (CH), 128.5 (CH), 128.5 (CH), 128.4 (CH), 128.1 (CH), 127.9 (CH), 127.5 (CH), 127.5 (CH), 127.4 (CH), 126.7 (CH), 101.7 (CH), 94.6 (CH), 93.6 (CH), 82.9 (CH), 82.2 (CH), 80.1 (CH), 79.4 (CH), 79.3 (CH), 78.1 (CH), 75.5 ($CH_2$), 75.3 ($CH_2$), 75.0 ($CH_2$), 74.1 ($CH_2$), 73.4 ($CH_2$), 70.2 (CH), 69.3 ($CH_2$), 63.7 ($CH_2$), 63.5 (CH), 45.2 ($CH_2$) ppm. IR (HATR): 3064 (w), 3030 (w), 2923 (w), 2871 (w), 1723 (m), 1513 (w), 1496 (m), 1453 (m), 1366 (m), 1331 (w), 1235 (m), 1210 (m), 1154 (m), 1138 (m), 1086 (s), 1073 (s), 985 (s), 915 (w), 735 (m), 696 (s), 652 (w) $cm^{-1}$. ESMS [m/z (fragment, intensity), API-ES positive mode]: 1031.4 ($M+NH_4^+$, 100). HRMS (ESI-TOF): calcd. for $C_{62}H_{67}N_2O_{12}^+$ $[M+NH_4]^+$ 1031.4689; found 1031.4681.

### *2,3,4,2′,3′-Penta-O-benzyl-4′,6′-O-benzylidene-6-O-[N-(2-phenethyl)-carbamoyl]-α,α-D-trehalose (18-f)*

General procedure B was applied (use of phenethyl isocyanate). Purification via column chromatography (gradient elution: hexane/EtOAc 8/2 to 7/3) afforded the title compound **18-f** as a white foam (220 mg, 0.214 mmol, 94%).

Rf 0.13 in hexane/EtOAc 7/3. $^{1}H$ NMR (400 MHz, benzene-$d_6$): $\delta$ 7.64–7.57 (m, 2H), 7.40–7.27 (m, 10H), 7.26–6.85 (m, 23H), 5.39 (s, 1H), 5.34 (d, $J=3.8$ Hz, 1H), 5.31 (d, $J=3.5$ Hz, 1H), 4.97 (d, $J=11.6$ Hz, 1H), 4.95 (d, $J=11.2$ Hz, 1H), 4.93 (d, $J=11.0$ Hz, 1H), 4.80–4.10 (m, 15H), 3.77–3.47 (m, 5H), 3.26–3.07 (m, 2H), 2.53–2.38 (m, 2H) ppm. $^{13}C$ NMR (100 MHz, benzene-$d_6$): $\delta$ 156.1 (C), 139.6 (C), 139.3 (C), 138.9 (C), 138.6 (C), 138.4 (C), 138.4 (C), 129.0 (CH), 128.9 (CH), 128.8 (CH), 128.7 (CH), 128.7 (CH), 128.5 (CH), 128.5 (CH), 128.4 (CH), 128.4 (CH), 128.3 (CH), 128.0 (CH), 128.0 (CH), 128.0 (CH), 127.8 (CH), 127.5 (CH), 127.5 (CH), 126.7 (CH), 126.6 (CH), 101.7 (CH), 94.6 (CH), 93.6 (CH), 82.9 (CH), 82.3 (CH), 80.1 (CH), 79.4 (CH), 79.3 (CH), 78.1 (CH), 75.5 ($CH_2$), 75.3 ($CH_2$), 75.0 ($CH_2$), 74.1 ($CH_2$), 73.4 ($CH_2$), 70.3 (CH), 69.3 ($CH_2$), 63.5 ($CH_2$), 63.5 (CH), 42.5 ($CH_2$), 36.3 ($CH_2$) ppm. IR (HATR): 3420 (w), 3060 (w), 3030 (w), 2932 (w), 2866 (w), 1722 (m), 1514 (w), 1496 (w), 1454 (m), 1391 (w), 1367 (w), 1329 (w), 1237 (w), 1210 (w), 1150 (w), 1137 (w), 1086 (s), 1072 (s), 984 (s), 913 (w), 874 (w), 849 (w), 824 (w), 798 (m), 734 (w), 695 (s), 656 (w) $cm^{-1}$. ESMS [m/z (fragment, intensity), API-ES positive mode]: 1045.4 ($M+NH_4^+$, 100). HRMS (ESI-TOF): calcd. for $C_{63}H_{69}N_2O_{12}^+$ $[M+NH_4]^+$ 1045.4845; found 1045.4829.

### *2,3,4,2′,3′-Penta-O-benzyl-4′,6′-O-benzylidene-6-O-(N-n-octylcarbamoyl)-α,α-D-trehalose (18-g)*

General procedure B was applied (use of *n*-octyl isocyanate). Purification via column chromatography (gradient elution: hexane/EtOAc 8/2 to 7/3) afforded the partially purified title compound **18-g** as a white foam (494 mg), which was used as such in the next step.

Rf 0.52 in hexane/EtOAc 6/4. HRMS (ESI-TOF): calcd. for $C_{63}H_{77}N_2O_{12}^+$ $[M+NH_4]^+$ 1053.5471; found 1053.5472.

### *6-O-(N-n-Propylcarbamoyl)-α,α-D-trehalose (19-a)*

General procedure C was applied. Purification via column chromatography (gradient elution: $CH_2Cl_2$/MeOH 8/2 to 7/3) afforded the title compound **19-a** as a white solid (64.0 mg, 0.150 mmol, 93%).

Rf 0.56 in $CH_3CN/H_2O$ 8/2, 0.11 in $CH_2Cl_2$/MeOH 9/1. $^{1}H$ NMR (400 MHz, $CD_3OD$): $\delta$ 5.08 (d, $J=2.8$ Hz, 1H), 5.07 (d, $J=3.2$ Hz, 1H), 4.25 (dd, $J=11.6/1.7$ Hz, 1H), 4.19 (dd, $J=11.7/4.9$ Hz, 1H), 3.98 (br ddd, $J=10.0/2.6/2.0$ Hz, 1H), 3.85–3.73 (m, 4H), 3.66 (dd, $J=12.0/5.5$ Hz, 1H), 3.55–3.31 (m, 4H), 3.05 (t, $J=7.0$ Hz, 2H), 1.49 (app sextet, $J=7.3$ Hz, 2H), 0.90 (t, $J=7.4$ Hz, 3H) ppm. $^{13}C$ NMR (100 MHz, $CD_3OD$): $\delta$ 159.2 (C), 95.2 (CH), 95.1 (CH), 74.6 (CH), 74.4 (CH), 73.9 (CH), 73.2 (CH), 73.2 (CH), 71.9 (CH), 71.8 (CH), 64.8 ($CH_2$), 62.6 ($CH_2$), 43.6 ($CH_2$), 24.1 ($CH_2$), 11.6 ($CH_3$) ppm. IR (HATR): 3304 (br, m), 2964 (w), 2933 (w), 1684 (m), 1540 (w), 1456 (w), 1419 (w), 1363 (w), 1337 (w), 1265 (m), 1147 (m), 1103 (m), 1076 (m), 1030 (m), 984 (s), 941 (w), 913 (w), 845 (w), 805 (w), 776 (w), 614 (w) $cm^{-1}$. $[\alpha]_D^{22}$ +150° (c 0.10, MeOH). ESMS [m/z (fragment, intensity), API-ES negative mode]: 486.2 ($M+OAc^-$, 100). HRMS (ESI-TOF): calcd. for $C_{16}H_{30}NO_{12}^+$ $[M+H]^+$ 428.1763; found 428.1771.

### *6-O-(N-Isopropylcarbamoyl)-α,α-D-trehalose (19-b)*

General procedure C was applied. Purification via column chromatography (gradient elution: $CH_2Cl_2$/MeOH 8/2 to 6/4) afforded the title compound **19-b** as a white solid (207 mg, 0.484 mmol, 93%).

Rf 0.55 in $CH_3CN/H_2O$ 8/2, 0.11 in $CH_2Cl_2$/MeOH 9/1. $^{1}H$ NMR (400 MHz, $CD_3OD$): $\delta$ 5.13–5.02 (m, 2H), 4.40–4.13 (m, 2H), 3.98 (br d, $J=8.9$ Hz, 1H), 3.85–3.63 (m, 6H), 3.46 (app dd, $J=9.8/3.7$ Hz, 2H), 3.37–3.26 (m, 2H), 1.12 (d, $J=6.5$ Hz, 6H) ppm. $^{13}C$ NMR (100 MHz, $CD_3OD$): $\delta$ 158.3 (C), 95.2 (CH), 95.1 (CH), 74.6 (CH), 74.4 (CH), 73.9 (CH), 73.2 (CH), 73.2 (CH), 71.9 (CH), 71.8 (CH), 64.7 ($CH_2$), 62.6 ($CH_2$), 44.0 (CH), 22.9 ($CH_3$) ppm. IR (HATR): 3303 (br, m), 2970 (w), 2932 (w), 1684 (m), 1540 (w), 1456 (w), 1436 (w), 1419 (w), 1388 (w), 1368 (w), 1349 (w), 1324 (w), 1254 (m), 1147 (w), 1103 (m), 1076 (m), 1042 (m), 1024 (m), 984 (s), 941 (m), 845 (w), 806 (w), 775 (w), 715 (w), 608 (w) $cm^{-1}$. $[\alpha]_D^{22}$ +222° (c 0.10, MeOH). ESMS [m/z (fragment, intensity), API-ES negative mode]: 486.2 ($M+OAc^-$, 100). HRMS (ESI-TOF): calcd. for $C_{16}H_{30}NO_{12}^+$ $[M+H]^+$ 428.1763; found 428.1772.

### *6-O-(N-Cycloheylcarbamoyl)-α,α-D-trehalose (19-c)*

General procedure C was applied. Purification via column chromatography (gradient elution: $CH_2Cl_2$/MeOH 9/1 to 6/4) afforded the title compound **19-c** as a white solid (170 mg, 0.364 mmol, 88%).

Rf 0.15 in $CH_2Cl_2$/MeOH 8/2. $^{1}H$ NMR (400 MHz, $CD_3OD$): $\delta$ 5.12–5.03 (m, 2H), 4.40–4.12 (m, 2H), 3.98 (br d, $J=9.2$ Hz, 1H), 3.85–3.73 (m, 4H), 3.66 (dd, $J=12.0/5.6$ Hz, 1H), 3.46 (dd, $J=9.8/3.7$ Hz, 2H), 3.40–3.26 (m, 3H), 1.86 (app br d, $J=11.0$ Hz, 2H), 1.80–1.68 (m, 2H), 1.66–1.56 (m, 1H), 1.33 (app qt, $J=12.5/3.1$ Hz, 2H), 1.25–1.09 (m, 3H), ppm. $^{13}C$ NMR (100 MHz, $CD_3OD$): $\delta$ 158.3 (C), 95.2 (CH), 95.1 (CH), 74.6 (CH), 74.4 (CH), 73.9 (CH), 73.2 (CH), 73.2 (CH), 71.9 (CH), 71.8 (CH), 64.8 ($CH_2$), 62.6 ($CH_2$), 51.3 (CH), 34.2 ($CH_2$), 26.6 ($CH_2$), 26.2 ($CH_2$) ppm. IR (HATR): 3293 (br, m), 2928 (m), 2858 (w), 1694 (m), 1538 (m), 1453 (m), 1418 (w), 1368 (w), 1317 (m), 1275 (m), 1253 (m), 1237 (m), 1145 (m), 1101 (m), 1076 (m), 1030 (s), 987 (s), 941 (m), 807 (w), 777 (w), 725 (m) $cm^{-1}$. $[\alpha]_D^{22}$ +166° (c 0.10, MeOH). ESMS [m/z (fragment, intensity), API-ES negative mode]: 933.3 (2M-$H^+$, 44), 526.2 ($M+OAc^-$, 100). HRMS (ESI-TOF): calcd. for $C_{19}H_{33}NNaO_{12}^+$ $[M+Na]^+$ 490.1895; found 490.1889.

### 6-O-(N-Phenylcarbamoyl)-α,α-D-trehalose (19-d)

General procedure C was applied. Purification via column chromatography (gradient elution: $CH_2Cl_2$/MeOH 8/2 to 7/3) afforded the title compound **19-d** as a white solid (41.0 mg, 0.089 mmol, 57%).

Rf 0.59 in $CH_3CN/H_2O$ 8/2, 0.15 in $CH_2Cl_2$/MeOH 9/1. $^1H$ NMR (400 MHz, $CD_3OD$): $\delta$ 7.41 (d, $J = 7.9$ Hz, 2H), 7.25 (t, $J = 8.0$ Hz, 2H), 7.00 (t, $J = 7.4$ Hz, 1H), 5.12 (app d, $J = 3.8$ Hz, 2H), 4.37 (dd, $J = 11.7/1.9$ Hz, 1H), 4.31 (dd, $J = 11.7/4.9$ Hz, 1H), 4.06 (ddd, $J = 10.0/4.8/2.3$ Hz, 1H), 3.87–3.72 (m, 4H), 3.66 (dd, $J = 11.8/5.3$ Hz, 1H), 3.54–3.28 (m, 6H) ppm. $^{13}C$ NMR (100 MHz, $CD_3OD$): $\delta$ 156.1 (C), 140.1 (C), 129.8 (CH), 124.1 (CH), 119.9 (CH), 95.3 (CH), 95.2 (CH), 74.6 (CH), 74.4 (CH), 73.9 (CH), 73.2 (CH), 73.2 (CH), 71.9 (CH), 71.8 (CH), 71.7 (CH), 65.0 ($CH_2$), 62.6 ($CH_2$) ppm. IR (HATR): 3302 (br, m), 2970 (w), 2932 (w), 1694 (m), 1684 (m), 1540 (w), 1455 (w), 1447 (w), 1419 (w), 1388 (w), 1368 (w), 1322 (w), 1251 (w), 1147 (w), 1100 (m), 1078 (m), 1039 (m), 1025 (m), 985 (s), 941 (w), 844 (w), 806 (w), 774 (w), 751 (w), 720 (w), 695 (w) $cm^{-1}$. $[\alpha]_D^{22}$ +152° (c 0.10, MeOH). ESMS [m/z (fragment, intensity), API-ES negative mode]: 460.2 (M-$H^+$, 100). HRMS (ESI-TOF): calcd. for $C_{19}H_{31}N_2O_{12}^+$ $[M + NH_4]^+$ 479.1872; found 479.1867.

### 6-O-(N-Benzylcarbamoyl)-α,α-D-trehalose (19-e)

General procedure C was applied. Purification via column chromatography (gradient elution: $CH_2Cl_2$/MeOH 8/2 to 6/4) afforded the title compound **19-e** as a white solid (200 mg, 0.421 mmol, 74% over 2 steps).

Rf 0.33 in $CH_3CN/H_2O$ 8/2, 0.09 in $CH_2Cl_2$/MeOH 8/2. $^1H$ NMR (400 MHz, $CD_3OD$): $\delta$ 7.36–7.17 (m, 5H), 5.09 (d, $J = 3.4$ Hz, 1H), 5.07 (d, $J = 3.4$ Hz, 1H), 4.30 (br dd, $J = 12.0/1.7$ Hz, 1H), 4.28 (s, 2H), 4.22 (dd, $J = 11.7/5.0$ Hz, 1H), 4.00 (br ddd, $J = 9.9/4.8/1.9$ Hz, 1H), 3.85–3.72 (m, 4H), 3.66 (dd, $J = 12.0/5.6$ Hz, 1H), 3.51–3.41 (m, 2H), 3.38–3.27 (m, 2H) ppm. $^{13}C$ NMR (100 MHz, $CD_3OD$): $\delta$ 159.3 (C), 140.6 (C), 129.5 (CH), 128.3 (CH), 128.1 (CH), 95.2 (CH), 95.1 (CH), 74.6 (CH), 74.4 (CH), 73.9 (CH), 73.2 (CH), 73.2 (CH), 71.9 (CH), 71.8 (CH), 65.1 ($CH_2$), 62.6 ($CH_2$), 45.5 ($CH_2$) ppm. IR (HATR): 3311 (m, br), 2930 (w), 1688 (m), 1540 (m), 1454 (w), 1433 (w), 1364 (w), 1338 (w), 1258 (m), 1147 (m), 1102 (m), 1076 (m), 1026 (m), 985 (s), 941 (m), 848 (w), 805 (w), 777 (w), 736 (w), 696 (w), 610 (w) $cm^{-1}$. $[\alpha]_D^{22}$ +140° (c 0.10, MeOH). ESMS [m/z (fragment, intensity), API-ES negative mode]: 949.2 (2 M-$H^+$, 47), 534.2 (M + $OAc^-$, 100). HRMS (ESI-TOF): calcd. for $C_{20}H_{29}NNaO_{12}^+$ $[M + Na]^+$ 498.1582; found 498.1579.

### 6-O-[N-(2-Phenethyl)carbamoyl]-α,α-D-trehalose (19-f)

General procedure C was applied. Purification via column chromatography (gradient elution: $CH_2Cl_2$/MeOH 8/2 to 7/3) afforded the title compound **19-f** as a white solid (85.8 mg, 0.175 mmol, 90%).

Rf 0.47 in $CH_3CN/H_2O$ 8/2, 0.35 in $CH_2Cl_2$/MeOH 7/3. $^1H$ NMR (400 MHz, $CD_3OD$): $\delta$ 7.31–7.13 (m, 5H), 5.08 (d, $J = 3.9$ Hz, 1H), 5.07 (d, $J = 3.9$ Hz, 1H), 4.27 (dd, $J = 11.7/1.7$ Hz, 1H), 4.20 (dd, $J = 11.8/4.9$ Hz, 1H), 3.98 (ddd, $J = 9.9/4.5/1.8$ Hz, 1H), 3.86–3.73 (m, 4H), 3.67 (dd, $J = 12.1/5.6$ Hz, 1H), 3.51–3.42 (m, 2H), 338–3.24 (m, 4H), 2.77 (t, $J = 7.4$ Hz, 2H) ppm. $^{13}C$ NMR (100 MHz, $CD_3OD$): $\delta$ 159.1 (C), 140.6 (C), 129.8 (CH), 129.5 (CH), 127.3 (CH), 95.2 (CH), 95.1 (CH), 74.6 (CH), 74.4 (CH), 73.9 (CH), 73.2 (CH), 73.2 (CH), 71.9 (CH), 71.8 (CH), 71.8 (CH), 64.8 ($CH_2$), 62.6 ($CH_2$), 43.5 ($CH_2$), 37.1 ($CH_2$) ppm. IR (HATR): 3306 (br, m), 3026 (w), 2931 (w), 1694 (m), 1540 (w), 1497 (w), 1454 (w), 1436 (w), 1419 (w), 1363 (w), 1332 (w), 1254 (m), 1204 (w), 1147 (m), 1103 (m), 1075 (m), 1047 (m), 1026 (m), 985 (s), 941 (m), 847 (w), 806 (w), 773 (w), 748 (w), 698 (w), 667 (w) $cm^{-1}$. $[\alpha]_D^{22}$ +152° (c 0.10, MeOH). ESMS [m/z (fragment, intensity), API-ES negative mode]: 977.3 (2 M-$H^+$, 60), 548.2 (M + $OAc^-$, 100). HRMS (ESI-TOF): calcd. for $C_{21}H_{32}NO_{12}^+$ $[M + H]^+$ 490.1919; found 490.1922.

### 6-O-(N-n-Octylcarbamoyl)-α,α-D-trehalose (19-g)

General procedure C was applied. Purification via column chromatography (gradient elution: $CH_2Cl_2$/MeOH 8/2 to 7/3) afforded the title compound **19-g** as a white solid (191 mg, 0.179 mmol, 84% over 2 steps).

Rf 0.50 in $CH_2Cl_2$/MeOH 6/4. $^1H$ NMR (400 MHz, $CD_3OD$): $\delta$ 5.14–5.03 (m, 2H), 4.30–4.14 (m, 2H), 3.99 (br d, $J = 7.8$ Hz, 1H), 3.87–3.74 (m, 4H), 3.67 (br dd, $J = 11.8/5.2$ Hz, 1H), 3.55–3.43 (m, 2H), 3.39–3.26 (m, 2H), 3.07 (app br t, $J = 6.7$ Hz, 2H), 1.55–1.20 (m, 12H), 0.95–0.83 (m, 3H) ppm. $^{13}C$ NMR (100 MHz, $CD_3OD$): $\delta$ 159.1 (C), 95.2 (CH), 95.1 (CH), 74.5 (CH), 74.3 (CH), 73.8 (CH), 73.2 (CH), 73.1 (CH), 71.9 (CH), 71.7 (CH), 64.8 ($CH_2$), 62.6 ($CH_2$), 41.8 ($CH_2$), 33.0 ($CH_2$), 30.9 ($CH_2$), 30.4 ($CH_2$), 30.4 ($CH_2$), 27.9 ($CH_2$), 23.7 ($CH_2$), 14.4 ($CH_3$) ppm. IR (HATR): 3314 (m, br), 2924 (m), 2857 (w), 1693 (m), 1539 (m), 1456 (w), 1371 (w), 1334 (w), 1260 (m), 1147 (m), 1106 (m), 1076 (m), 1025 (m), 986 (s), 941 (m), 912 (w), 853 (w), 805 (w), 777 (w) $cm^{-1}$. $[\alpha]_D^{22}$ +171° (c 0.10, MeOH). ESMS [m/z (fragment, intensity), API-ES negative mode]: 993.4 (2 M-$H^+$, 45), 556.2 (M + $OAc^-$, 100). HRMS (ESI-TOF): calcd. for $C_{21}H_{39}NNaO_{12}^+$ $[M + Na]^+$ 520.2365; found 520.2355.

### 2,3,6,2′,3′-Penta-O-benzyl-4′,6′-O-benzylidene-4-O-methyl-α,α-D-trehalose (20-a)

General procedure A was applied (use of iodomethane). Purification via column chromatography (gradient elution: hexane/EtOAc 9/1 to 8/2 containing 1% $Et_3N$) afforded the title compound **20-a** as a colourless glass (194 mg, 0.217 mmol, 95%).

Rf 0.39 in hexane/EtOAc 7/3. $^1H$ NMR (400 MHz, benzene-$d_6$): $\delta$ 7.63–7.56 (m, 2H), 7.39–7.28 (m, 10H), 7.24–7.02 (m, 18H), 5.39 (d, $J = 3.8$ Hz, 1H), 5.37 (s, 1H), 5.32 (d, $J = 3.5$ Hz, 1H), 4.94 (d, $J = 11.6$ Hz, 1H), 4.92 (d, $J = 11.4$ Hz, 1H), 4.80 (d, $J = 11.6$ Hz, 1H), 4.76 (d, $J = 11.3$ Hz, 1H), 4.70 (d, $J = 11.6$ Hz, 1H), 4.69 (d, $J = 12.1$ Hz, 1H), 4.59–4.43 (m, 6H), 4.37 (app t, $J = 9.3$ Hz, 1H), 4.28 (app t, $J = 9.3$ Hz, 1H), 4.18 (dd, $J = 10.1/4.9$ Hz, 1H), 3.72–3.48 (m, 7H), 3.45 (s, 3H) ppm. $^{13}C$ NMR (100 MHz, benzene-$d_6$): $\delta$ 140.2 (C), 140.1 (C), 139.5 (C), 139.3 (C), 139.1 (C), 138.9 (C), 129.3 (CH), 129.1 (CH), 129.0 (CH), 128.9 (CH), 128.8 (CH), 128.7 (CH), 127.8 (CH), 127.8 (CH), 127.1 (CH), 102.0 (CH), 94.8 (CH), 94.1 (CH), 83.3 (CH), 82.7 (CH), 80.6 (CH), 80.6 (CH), 79.7 (CH), 79.6 (CH), 75.7 ($CH_2$), 75.6 ($CH_2$), 74.1 ($CH_2$), 74.0 ($CH_2$), 74.0 ($CH_2$), 72.1 (CH), 70.0 ($CH_2$), 69.6 ($CH_2$), 63.8 (CH), 60.7 (CH) ppm. IR (HATR): 3032 (w), 2921 (m), 2859 (w), 1728 (w), 1496 (w), 1453 (m), 1369 (m), 1331 (w), 1262 (w), 1208 (w), 1154 (m), 1138 (m), 1086 (s), 1049 (m), 986 (s), 913 (w), 797 (w), 733 (m), 695 (s), 656 (w) $cm^{-1}$. ESMS [m/z (fragment, intensity), API-ES positive mode]: 912.3 (M + $NH_4^+$, 100). HRMS (ESI-TOF): calcd. for $C_{55}H_{62}NO_{11}^+$ $[M + NH_4]^+$ 912.4317; found 912.4333.

### 2,3,6,2′,3′-Penta-O-benzyl-4′,6′-O-benzylidene-4-O-ethyl-α,α-D-trehalose (20-b)

General procedure A was applied (use of iodoethane). Purification via column chromatography (gradient elution: hexane/EtOAc 9/1 to 8/2 containing 1% $Et_3N$) afforded the title compound **20-b** as a colourless glass (195 mg, 0.215 mmol, 83%).

Rf 0.35 in hexane/EtOAc7/3. $^1H$ NMR (400 MHz, benzene-$d_6$): $\delta$ 7.62–7.56 (m, 2H), 7.39–7.27 (m, 10H), 7.23–7.01 (m, 18H), 5.40 (d, $J = 3.7$ Hz, 1H), 5.37 (s, 1H), 5.32 (d, $J = 3.7$ Hz, 1H), 4.97–4.89 (m,

2H), 4.81 (d, $J = 11.4$ Hz, 1H), 4.76 (d, $J = 12.1$ Hz, 1H), 4.70 (d, $J = 11.6$ Hz, 1H), 4.68 (d, $J = 12.1$ Hz, 1H), 4.61–4.40 (m, 7H), 4.37 (app t, $J = 9.2$ Hz, 1H), 4.28 (app t, $J = 9.3$ Hz, 1H), 4.18 (dd, $J = 10.3/4.9$ Hz, 1H), 3.94–3.82 (m, 1H), 3.73–3.48 (m, 8H), 1.08 (t, $J = 7.0$ Hz, 3H) ppm. $^{13}$C NMR (100 MHz, benzene-d$_6$): $\delta$ 140.3 (C), 140.2 (C), 139.5 (C), 139.4 (C), 139.1 (C), 138.9 (C), 129.3 (CH), 129.1 (CH), 129.0 (CH), 128.9 (CH), 128.8 (CH), 128.7 (CH), 128.3 (CH), 128.3 (CH), 128.1 (CH), 127.8 (CH), 127.1 (CH), 102.0 (CH), 94.8 (CH), 94.1 (CH), 83.3 (CH), 82.7 (CH), 80.6 (CH), 79.7 (CH), 79.6 (CH), 79.0 (CH), 75.8 CH$_2$), 75.6 (CH$_2$), 74.1 (CH$_2$), 74.1 (CH$_2$), 74.0 (CH$_2$), 72.2 (CH), 69.9 (CH$_2$), 68.7 (CH$_2$), 63.8 (CH), 16.5 (CH$_3$) ppm. IR (HATR): 3064 (w), 3030 (w), 2968 (w), 2923 (w), 2863 (w), 1496 (w), 1453 (m), 1370 (m), 1329 (w), 1208 (w), 1151 (m), 1135 (m), 1085 (s), 1076 (s), 1049 (m), 1013 (m), 984 (s), 915 (w), 877 (w), 796 (w), 733 (m), 695 (s), 656 (w), 632 (w) cm$^{-1}$. ESMS [m/z (fragment, intensity), API-ES positive mode]: 926.4 (M + NH$_4^+$, 100). HRMS (ESI-TOF): calcd. for $C_{56}H_{64}NO_{11}^+$ [M + NH$_4$]$^+$ 926.4474; found 926.4491.

### *4-O-Allyl-2,3,6,2′,3′-penta-O-benzyl-4′,6′-O-benzylidene-α,α-D-trehalose (20-c)*

General procedure A was applied (use of allyl bromide). Purification via column chromatography (gradient elution: hexane/EtOAc 9/1 to 75/25 containing 1% Et$_3$N) afforded the title compound **20-c** as a colourless glass (179 mg, 0.194 mmol, 93%).

Rf 0.41 in hexane/EtOAc 7/3. $^1$H NMR (400 MHz, benzene-d$_6$): $\delta$ 7.63–7.57 (m, 2H), 7.40–7.27 (m, 10H), 7.24–7.01 (m, 18H), 5.85 (app ddt, $J = 17.2/10.5/5.3$ Hz, 1H), 5.39 (d, $J = 3.7$ Hz, 1H), 5.37 (s, 1H), 5.32 (d, $J = 3.7$ Hz, 1H), 5.21 (app dq, $J = 17.2/1.7$ Hz, 1H), 5.01 (app dq, $J = 10.5/1.4$ Hz, 1H), 4.94 (d, $J = 11.5$ Hz, 1H), 4.92 (d, $J = 11.1$ Hz, 1H), 4.79 (d, $J = 11.2$ Hz, 1H), 4.77 (d, $J = 12.1$ Hz, 1H), 4.74–4.64 (m, 2H), 4.59–4.34 (m, 8H), 4.30 (app t, $J = 9.3$ Hz, 1H), 4.18 (dd, $J = 10.3/4.9$ Hz, 1H), 4.14 (app ddt, $J = 12.9/5.3/1.5$ Hz, 1H), 3.78–3.47 (m, 7H) ppm. $^{13}$C NMR (100 MHz, benzene-d$_6$): $\delta$ 139.8 (C), 139.7 (C), 139.0 (C), 139.0 (C), 138.7 (C), 138.5 (C), 135.8 (CH), 128.9 (CH), 128.7 (CH), 128.6 (CH), 128.4 (CH), 128.3 (CH), 128.0 (CH), 127.9 (CH), 127.9 (CH), 127.9 (CH), 127.8 (CH), 127.7 (CH), 127.4 (CH), 126.8 (CH), 115.7 (CH$_2$), 101.6 (CH), 94.4 (CH), 93.7 (CH), 83.0 (CH), 82.3 (CH), 80.2 (CH), 79.3 (CH), 78.3 (CH), 75.4 (CH$_2$), 75.2 (CH$_2$), 73.8 (CH$_2$), 73.7 (CH$_2$), 73.7 (CH$_2$), 73.6 (CH$_2$), 71.7 (CH), 69.5 (CH$_2$), 69.3 (CH$_2$), 63.4 (CH) ppm. IR (HATR): 3064 (w), 3032 (w), 2927 (w), 2864 (w), 1496 (w), 1453 (m), 1367 (m), 1328 (w), 1208 (w), 1135 (m), 1083 (s), 1072 (s), 985 (s), 927 (m), 875 (w), 796 (w), 733 (m), 695 (s), 656 (w) cm$^{-1}$. ESMS [m/z (fragment, intensity), API-ES positive mode]: 938.4 (M + NH$_4^+$, 100). HRMS (ESI-TOF): calcd. for $C_{57}H_{64}NO_{11}^+$ [M + H]$^+$ 938.4474; found 938.4488.

### *2,3,6,2′,3′-Penta-O-benzyl-4′,6′-O-benzylidene-4-O-n-butyl-α,α-D-trehalose (20-d)*

General procedure A was applied (use of iodobutane). Purification via column chromatography (gradient elution: hexane/EtOAc 9/1 to 8/2) afforded the title compound **20-d** as a colourless glass (205 mg, 0.219 mmol, 96%).

Rf 0.17 in hexane/EtOAc 8/2. $^1$H NMR (400 MHz, benzene-d$_6$): $\delta$ 7.63–7.56 (m, 2H), 7.40–7.28 (m, 10H), 7.24–7.00 (m, 18H), 5.41 (d, $J = 3.8$ Hz, 1H), 5.38 (s, 1H), 5.34 (d, $J = 3.5$ Hz, 1H), 4.95 (d, $J = 11.6$ Hz, 1H), 4.93 (d, $J = 11.7$ Hz, 1H), 4.81 (d, $J = 11.4$ Hz, 1H), 4.76 (d, $J = 12.0$ Hz, 1H), 4.69 (d, $J = 11.6$ Hz, 1H), 4.68 (d, $J = 12.1$ Hz, 1H), 4.59–4.44 (m, 6H), 4.38 (app t, $J = 9.3$ Hz, 1H), 4.28 (app t, $J = 9.3$ Hz, 1H), 4.19 (dd, $J = 10.2/4.9$ Hz, 1H), 3.92 (app t, $J = 6.2$ Hz, 1H), 3.89 (app t, $J = 6.2$ Hz, 1H), 3.75–3.48 (m, 8H), 1.58–1.20 (m, 2H), 0.84 (t, $J = 7.3$ Hz, 3H) ppm. $^{13}$C NMR (100 MHz, CDCl$_3$): $\delta$ 139.9 (C), 139.8 (C), 139.1 (C), 139.0 (C), 138.7 (C), 138.5 (C), 128.9 (CH), 128.7 (CH), 128.6 (CH), 128.6 (CH), 128.4 (CH), 128.3 (CH), 128.0 (CH), 127.9 (CH), 127.7 (CH), 127.4 (CH), 127.4 (CH), 126.8 (CH), 101.6 (CH), 94.4 (CH), 93.7 (CH), 83.0 (CH), 82.4 (CH), 80.3 (CH), 79.3 (CH), 78.6 (CH), 75.2 (CH$_2$), 73.8 (CH$_2$), 73.7 (CH$_2$), 72.8 (CH$_2$), 71.9 (CH), 69.5 (CH$_2$), 69.3 (CH$_2$), 63.4 (CH), 33.0 (CH$_2$), 19.7 (CH$_2$), 14.2 (CH) ppm. IR (HATR): 3064 (w), 3030 (w), 2930 (w), 2862 (w), 1496 (w), 1453 (m), 1366 (m), 1329 (w), 1208 (w), 1135 (m), 1086 (s), 1049 (m), 985 (s), 913 (m), 796 (w), 733 (m), 695 (s), 657 (m) cm$^{-1}$. ESMS [m/z (fragment, intensity), API-ES positive mode]: 954.4 (M + NH$_4^+$, 100). HRMS (ESI-TOF): calcd. for $C_{58}H_{68}NO_{11}^+$ [M + NH$_4$]$^+$ 954.4787; found 954.4788.

### *2,3,6,2′,3′-Penta-O-benzyl-4′,6′-O-benzylidene-4-O-(2-methylallyl)-α,α-D-trehalose (20-e)*

General procedure A was applied (use of 3-bromo-2-methylpropene, "methallyl bromide"). Purification via column chromatography (gradient elution: hexane/EtOAc 9/1 to 8/2 containing 1% Et$_3$N) afforded the title compound **20-e** as a colourless glass (203 mg, 0.217 mmol, 88%).

Rf 0.07 in hexane/EtOAc 9/1. $^1$H NMR (400 MHz, benzene-d$_6$): $\delta$ 7.64–7.56 (m, 2H), 7.43–7.27 (m, 10H), 7.24–7.02 (m, 18H), 5.41 (d, $J = 3.8$ Hz, 1H), 5.38 (s, 1H), 5.33 (d, $J = 3.7$ Hz, 1H), 5.10–5.03 (m, 1H), 4.94 (d, $J = 11.7$ Hz, 1H), 4.93 (d, $J = 11.2$ Hz, 1H), 4.86–4.81 (m, 1H), 4.78 (d, $J = 11.4$ Hz, 1H), 4.77 (d, $J = 12.0$ Hz, 1H), 4.70 (d, $J = 11.7$ Hz, 1H), 4.66 (d, $J = 12.0$ Hz, 1H), 4.59–4.26 (m, 9H), 4.19 (dd, $J = 10.1/4.9$ Hz, 1H), 4.03 (d, $J = 12.1$ Hz, 1H), 3.78–3.48 (m, 7H), 1.61 (s, 3H) ppm. $^{13}$C NMR (100 MHz, benzene-d$_6$): $\delta$ 143.0 (C), 139.8 (C), 139.7 (C), 139.0 (C), 139.0 (C), 138.6 (C), 138.5 (C), 128.9 (CH), 128.7 (CH), 128.6 (CH), 128.4 (CH), 128.3 (CH), 128.0 (CH), 127.9 (CH), 127.8 (CH), 127.7 (CH), 127.4 (CH), 127.4 (CH), 126.8 (CH), 111.4 (CH$_2$), 101.6 (CH), 94.4 (CH), 93.7 (CH), 83.0 (CH), 82.4 (CH), 80.3 (CH), 79.4 (CH), 79.3 (CH), 78.5 (CH), 76.8 (CH$_2$), 75.4 (CH$_2$), 75.2 (CH$_2$), 73.9 (CH$_2$), 73.6 (CH$_2$), 71.7 (CH), 69.5 (CH$_2$), 69.3 (CH$_2$), 63.4 (CH), 19.7 (CH$_3$) ppm. IR (HATR): 3085 (w), 3065 (w), 3030 (w), 2930 (w), 2862 (w), 1496 (w), 1453 (m), 1366 (m), 1328 (w), 1208 (w), 1155 (m), 1137 (m), 1085 (s), 1075 (s), 1049 (m), 984 (s), 907 (m), 796 (w), 733 (m), 695 (s), 656 (w), 631 (w) cm$^{-1}$. ESMS [m/z (fragment, intensity), API-ES positive mode]: 952.4 (M + NH$_4^+$, 100). HRMS (ESI-TOF): calcd. for $C_{58}H_{66}NO_{11}^+$ [M + NH$_4$]$^+$ 952.4630; found 952.4623.

### *2,3,6,2′,3′-Penta-O-benzyl-4′,6′-O-benzylidene-4-O-(3,3-dimethylallyl)-α,α-D-trehalose (20-f)*

General procedure A was applied (use of 1-bromo-3-methyl-2-butene, "prenyl bromide"). Purification via column chromatography (gradient elution: hexane/EtOAc 9/1 to 85/15 containing 1% Et$_3$N) afforded the title compound **20-f** as a colourless glass (665 mg, 0.701 mmol, 78%).

Rf 0.38 in hexane/EtOAc 7/3. $^1$H NMR (400 MHz, benzene-d$_6$): $\delta$ 7.63–7.54 (m, 2H), 7.42–7.26 (m, 10H), 7.23–7.01 (m, 18H), 5.53–5.46 (m, 1H), 5.40 (d, $J = 3.8$ Hz, 1H), 5.37 (s), 1H), 5.34 (d, $J = 3.7$ Hz, 1H), 5.00–4.83 (m, 3H), 4.80–4.66 (m, 3H), 4.59–4.44 (m, 7H), 4.42–4.25 (m, 3H), 4.18 (dd, $J = 10.2/4.9$ Hz, 1H), 3.80–3.69 (m, 3H), 3.62 (dd, $J = 7.3/3.7$ Hz, 1H), 3.60 (dd, $J = 7.6/3.7$ Hz, 1H), 3.54 (app t, $J = 9.5$ Hz, 1H), 3.52 (app t, $J = 10.4$ Hz, 1H), 1.59 (s, 3H), 1.45 (s, 3H) ppm. $^{13}$C NMR (100 MHz, benzene-d$_6$): $\delta$ 140.3 (C), 140.2 (C), 139.5 (C), 139.4 (C), 139.1 (C), 138.9 (C), 135.5 (C), 129.3 (CH), 129.1 (CH), 129.0 (CH), 128.8 (CH), 128.7 (CH), 128.4 (CH),

128.3 (CH), 128.3 (CH), 128.2 (CH), 128.1 (CH), 127.8 (CH), 127.8 (CH), 127.1 (CH), 123.3 (CH), 102.0 (CH), 94.8 (CH), 94.1 (CH), 83.3 (CH), 82.9 (CH), 80.7 (CH), 79.7 (CH), 79.6 (CH), 78.3 (CH), 75.8 ($CH_2$), 75.6 ($CH_2$), 74.1 ($CH_2$), 72.3 (CH), 70.0 ($CH_2$), 69.7 ($CH_2$), 63.8 (CH), 26.1 ($CH_3$), 18.3 ($CH_3$) ppm. IR (HATR): 3085 (w), 3059 (w), 3030 (w), 2930 (w), 2861 (w), 1496 (w), 1453 (m), 1366 (m), 1328 (w), 1279 (vw), 1208 (w), 1153 (m), 1137 (m), 1070 (s), 984 (s), 913 (m), 874 (vw), 840 (w), 796 (w), 732 (s), 695 (s), 656 (m), 631 (w) $cm^{-1}$. ESMS [m/z (fragment, intensity), API-ES positive mode]: 966.4 ($M+NH_4^+$, 100). HRMS (ESI-TOF): calcd. for $C_{59}H_{68}NO_{11}^+$ $[M+NH_4]^+$ 966.4787; found 966.4791.

### *2,3,6,2′,3′-Penta-O-benzyl-4′,6′-O-benzylidene-4-O-n-decyl-α,α-D-trehalose (20-g)*

General procedure A was applied (use of bromodecane). Purification via column chromatography (gradient elution: hexane/EtOAc 10/0 to 8/2 containing 1% $Et_3N$) afforded the title compound **20-g** as a colourless glass (236 mg, 0.231 mmol, 93%).

Rf 0.07 in hexane/EtOAc 7/3. $^1$H NMR (400 MHz, benzene-$d_6$): $\delta$ 7.63–7.55 (m, 2H), 7.43–7.27 (m, 10H), 7.26–7.02 (m, 18H), 5.41 (d, $J=3.8$ Hz, 1H), 5.37 (s, 1H), 5.34 (d, $J=3.7$ Hz, 1H), 4.97 (d, $J=11.5$ Hz, 1H), 4.93 (d, $J=11.6$ Hz, 1H), 4.85 (d, $J=11.5$ Hz, 1H), 4.76 (d, $J=12.0$ Hz, 1H), 4.68 (app d, $J=11.5$ Hz, 2H), 4.60–4.44 (m, 6H), 4.38 (app t, $J=9.3$ Hz, 1H), 4.30 (app t, $J=9.3$ Hz, 1H), 4.18 (dd, $J=10.1/4.9$ Hz, 1H), 4.00–3.90 (m, 1H), 3.80–3.48 (m, 8H), 1.64–1.47 (m, 2H), 1.43–1.18 (m, 14H), 0.92 (t, $J=6.8$ Hz, 3H) ppm. $^{13}$C NMR (100 MHz, benzene-$d_6$): $\delta$ 139.9 (C), 139.8 (C), 139.1 (C), 139.0 (C), 138.7 (C), 138.5 (C), 128.9 (CH), 128.7 (CH), 128.6 (CH), 128.6 (CH), 128.4 (CH), 128.3 (CH), 127.9 (CH), 127.9 (CH), 127.7 (CH), 127.6 (CH), 127.4 (CH), 127.4 (CH), 101.6 (CH), 94.4 (CH), 93.7 (CH), 83.0 (CH), 82.4 (CH), 80.3 (CH), 79.3 (CH), 79.3 (CH), 78.6 (CH), 75.4 ($CH_2$), 75.2 ($CH_2$), 73.7 ($CH_2$), 73.7 ($CH_2$), 73.7 ($CH_2$), 73.2 ($CH_2$), 71.9 (CH), 69.6 ($CH_2$), 69.3 ($CH_2$), 63.4 (CH), 32.3 ($CH_2$), 31.0 ($CH_2$), 30.1 ($CH_2$), 30.1 ($CH_2$), 30.0 ($CH_2$), 29.8 ($CH_2$), 26.7 ($CH_2$), 23.1 ($CH_2$), 14.3 ($CH_3$) ppm. IR (HATR): 3085 (w), 3059 (w), 3030 (w), 2924 (m), 2854 (m), 1496 (w), 1453 (m), 1366 (m), 1328 (w), 1208 (w), 1153 (m), 1137 (m), 1087 (s), 1049 (m), 986 (s), 913 (w), 874 (w), 796 (w), 732 (m), 695 (s), 656 (w), 634 (w) $cm^{-1}$. ESMS [m/z (fragment, intensity), API-ES positive mode]: 1038.5 ($M+NH_4^+$, 100). HRMS (ESI-TOF): calcd. for $C_{64}H_{80}NO_{11}^+$ $[M+NH_4]^+$ 1038.5726; found 1038.5713.

### *2,3,6,2′,3′-Penta-O-benzyl-4′,6′-O-benzylidene-4-O-[(2S)-2,3-dihydroxy-3-methylbut-1-yl]-α,α-D-trehalose (20-h)*

In a sealed tube AD-mix α (1.21 g), $MeSO_2NH_2$ (106 mg, 1.11 mmol, 2.5 eq) and $(DHQ)_2PHAL$ (95 mg, 0.122 mmol, 0.28 eq) were dissolved in $H_2O$/tert-butanol (1/1, 18.4 ml) and the solution was stirred for 30 min. The obtained yellow solution was cooled to 0 °C and stirred for 10 min at 0 °C. To this, a cooled (0 °C) solution of **20-f** (418 mg, 0.440 mmol, 1 eq) in acetone (8.3 ml) was added and the resulting mixture was stirred for 10 d at 0–5 °C. An aqueous $Na_2SO_3$ solution (10% w/v, 3 ml) was added and the resulting mixture was stirred for 2 h at 0 °C after which it was transferred to a separation funnel using $H_2O$ (10 ml) and EtOAc (30 ml). After separation of the organic layer, the aqueous phase was extracted with EtOAc (2 × 30 ml). The combined organic layers were washed with brine (100 ml) and concentrated under reduced pressure. The residue was purified using column chromatography (gradient elution: hexane/EtOAc 75/25 to 6/4 containing 1% $Et_3N$) to give the title compound **20-h** as a colourless glass (240 mg, 0.244 mmol, 55%, 73% de based on $^1$H NMR analysis).

Rf 0.50 in hexane/EtOAc 1/1. $^1$H NMR (300 MHz, $CDCl_3$): $\delta$ 7.54–7.22 (m, 30H), 5.55 (s, 1H), 5.18–5.11 (m, 2H), 5.05–4.60 (m, 8H), 4.52 (app d, $J=12.1$ Hz, 1H, A-part of AB-system), 4.41 (app d, $J=12.1$ Hz, 1H, B-part of AB-system), 4.31–3.98 (m, 4H), 3.92 (app t, $J=9.3$ Hz, 1H), 3.83 (app dd, $J=9.5/2.2$ Hz, 1H), 3.72–3.28 (m, 9H), 2.94 (br s, OH), 2.29 (br s, OH), 1.10 (app br s, 3H), 1.02 (s, ~0.4H, minor diastereoisomer), 0.98 (s, ~2.6H, major diastereoisomer) ppm. $^{13}$C NMR (75 MHz, $CDCl_3$, only major diastereoisomer visible): $\delta$ 138.8 (C), 138.4 (C), 138.2 (C), 137.8 (C), 137.6 (C), 137.5 (C), 128.9 (CH), 128.5 (CH), 128.4 (CH), 128.3 (CH), 128.2 (CH), 128.0 (CH), 128.0 (CH), 127.9 (CH), 127.8 (CH), 127.8 (CH), 127.7 (CH), 127.6 (CH), 127.5 (CH), 126.1 (CH), 101.2 (CH), 95.0 (CH), 94.2 (CH), 82.4 (CH), 81.2 (CH), 79.5 (CH), 78.9 (CH), 78.7 (CH), 78.0 (CH), 76.2 (CH), 75.6 ($CH_2$), 75.2 ($CH_2$), 73.7 ($CH_2$), 73.6 ($CH_2$), 73.6 ($CH_2$), 73.0 ($CH_2$), 71.2 (C), 70.5 (CH), 69.0 ($CH_2$), 68.4 ($CH_2$), 63.0 (CH), 26.5 ($CH_3$), 24.5 ($CH_3$) ppm. IR (HATR): 3480 (w, br), 3086 (w), 3064 (w), 3027 (w), 2927 (w), 2863 (w), 1496 (w), 1453 (m), 1368 (m), 1331 (w), 1209 (w), 1135 (m), 1086 (s), 1072 (s), 1047 (m), 1011 (m), 985 (s), 915 (m), 877 (w), 797 (w), 734 (m), 695 (s), 656 (w) $cm^{-1}$. ESMS [m/z (fragment, intensity), API-ES positive mode]: 1000.3 ($M+NH_4^+$, 100). ESMS [m/z (fragment, intensity), API-ES negative mode]: 1041.3 ($M+OAc^-$, 100). HRMS (ESI-TOF): calcd. for $C_{59}H_{70}NO_{13}$ $[M+NH_4]^+$ 1000.4842; found 1000.4865.

### *4-O-Methyl-α,α-D-trehalose (21-a)*

General procedure C was applied. As the product was poorly soluble in MeOH, the celite layer was additionally washed with water and the filtrate was lyophilised. The resulting solid was washed with MeOH to afford the title compound **21-a** as a white solid (46.7 mg, 0.131 mmol, 80%).

Rf 0.30 in $CH_3CN/H_2O$ 8/2, 0.35 in $CH_2Cl_2$/MeOH 6/4. $^1$H NMR (400 MHz, $D_2O$): $\delta$ 5.14 (d, $J=3.8$ Hz, 1H), 5.14 (d, $J=3.8$ Hz, 1H), 3.89 (app t, $J=9.6$ Hz, 1H), 3.85–3.68 (m, 7H), 3.62 (dd, $J=9.8/3.9$ Hz, 1H), 3.60 (dd, $J=9.8/3.9$ Hz, 1H), 3.54 (s, 3H), 3.40 (app t, $J=9.4$ Hz, 1H), 3.22 (app t, $J=9.4$ Hz, 1H) ppm. $^{13}$C NMR (100 MHz, $D_2O$): $\delta$ 93.2 (CH), 93.1 (CH), 79.4 (CH), 72.5 (CH), 72.3 (CH), 72.1 (CH), 71.1 (CH), 71.0 (CH), 71.0 (CH), 69.7 (CH), 60.5 ($CH_2$), 60.2 ($CH_2$), 60.1 ($CH_3$) ppm. IR (HATR): 3488 (w), 3478 (w), 3400 (br, m), 3300 (w), 3160 (w), 3012 (w), 2976 (w), 2938 (w), 2920 (w), 2874 (w), 1654 (w), 1594 (w), 1456 (m), 1414 (w), 1380 (w), 1354 (w), 1304 (w), 1288 (w), 1250 (w), 1206 (w), 1190 (w), 1148 (m), 1132 (m), 1116 (m), 1098 (m), 1072 (m), 1044 (m), 1022 (m), 980 (s), 964 (s), 938 (m), 904 (m), 868 (w), 854 (w), 828 (w), 804 (w), 770 (w), 726 (w), 710 (w), 698 (w), 654 (w) $cm^{-1}$. ESMS [m/z (fragment, intensity), API-ES positive mode]: 374.1 ($M+NH_4^+$, 100), 339.1 ($M-H_2O+H^+$, 40), 177.1 ($M$-glucose$+H^+$, 85). HRMS (ESI-TOF): calcd. for $C_{13}H_{24}NaO_{11}^+$ $[M+Na]^+$ 379.1211; found 379.1225.

### *4-O-Ethyl-α,α-D-trehalose (21-b)*

General procedure C was applied. Purification via column chromatography (gradient elution: $CH_2Cl_2$/MeOH 8/2 to 7/3) afforded the title compound **21-b** as a white solid (59.9 mg, 0.162 mmol, 90%).

Rf 0.27 in $CH_3CN/H_2O$ 8/2, 0.22 in $CH_2Cl_2$/MeOH 7/3. $^1$H NMR (400 MHz, $CD_3OD$): $\delta$ 5.10 (d, $J=3.8$ Hz, 1H), 5.08 (d, $J=3.7$ Hz, 1H), 3.95–3.72 (m, 7H), 3.71–3.60 (m, 3H), 3.51–3.42 (m, 2H), 3.33–3.27 (m, 1H), 3.21 (dd, $J=9.7/9.2$ Hz, 1H), 1.17 (t, $J=7.0$ Hz, 3H) ppm. $^{13}$C NMR (100 MHz, $CD_3OD$): $\delta$ 95.1 (CH), 95.0 (CH), 79.6 (CH), 74.6 (CH), 74.5 (CH), 73.8 (CH), 73.3 (CH), 73.2 (CH), 72.9 (CH), 71.9 (CH), 69.1 ($CH_2$), 62.6 ($CH_2$), 62.1 ($CH_2$), 16.0 ($CH_3$) ppm. IR (HATR): 3308 (br, m), 2970 (w), 2929 (w), 1440 (w), 1413 (w), 1374 (w), 1332 (w), 1248 (w), 1204 (w), 1145 (m), 1103 (m), 1030 (m), 984 (s), 941 (m),

877 (w), 843 (w), 799 (w), 706 (w) $cm^{-1}$. $[\alpha]_D^{25}$ +164° (c 0.310, MeOH). ESMS [m/z (fragment, intensity), API-ES positive mode]: 388.1 ($M+Na^+$, 100). HRMS (ESI-TOF): calcd. for $C_{14}H_{26}NaO_{11}^+$ $[M+Na]^+$ 393.1367; found 393.1365.

### *4-O-n-Propyl-α,α-D-trehalose (21-c)*

General procedure C was applied. Purification via column chromatography (gradient elution: $CH_2Cl_2$/MeOH 8/2 to 7/3) afforded the title compound **21-c** as a white solid (57.3 mg, 0.149 mmol, 90%).

Rf 0.30 in $CH_3CN/H_2O$ 8/2, 0.30 in $CH_2Cl_2$/MeOH 7/3. $^1H$ NMR (400 MHz, $CD_3OD$): $\delta$ 5.10 (d, $J=3.9$ Hz, 1H), 5.09 (d, $J=3.9$ Hz, 1H), 3.92–3.72 (m, 7H), 3.71–3.62 (m, 2H), 3.58–3.43 (m, 3H), 3.36–3.28 (m, 1H), 3.20 (dd, $J=9.8/9.2$ Hz, 1H), 1.67–1.48 (m, 2H), 0.92 (t, $J=7.4$ Hz, 3H) ppm. $^{13}C$ NMR (100 MHz, $CD_3OD$): $\delta$ 95.0 (CH), 79.6 (CH), 75.5 ($CH_2$), 74.6 (CH), 74.5 (CH), 73.7 (CH), 73.4 (CH), 73.2 (CH), 72.9 (CH), 71.8 (CH), 62.6 ($CH_2$), 62.1 ($CH_2$), 24.5 ($CH_2$), 10.9 ($CH_3$) ppm. IR (HATR): 3326 (br, m), 2934 (w), 2880 (w), 1455 (w), 1416 (w), 1363 (w), 1257 (w), 1204 (w), 1145 (m), 1103 (m), 1072 (m), 1030 (m), 987 (s), 843 (w), 802 (w), 706 (w), 617 (w) $cm^{-1}$. $[\alpha]_D^{25}$ +183° (c 0.557, MeOH). ESMS [m/z (fragment, intensity), API-ES positive mode]: 402.2 ($M+NH_4^+$, 100). HRMS (ESI-TOF): calcd. for $C_{15}H_{28}NaO_{11}^+$ $[M+Na]^+$ 407.1524; found 407.1520.

### *4-O-n-Butyl-α,α-D-trehalose (21-d)*

General procedure C was applied. Purification via column chromatography (gradient elution: $CH_2Cl_2$/MeOH 9/1 to 6/4) afforded the title compound **21-d** as a white solid (75 mg, 0.188 mmol, 100%).

Rf 0.40 in $CH_3CN/H_2O$ 8/2, 0.14 in $CH_2Cl_2$/MeOH 8/2. $^1H$ NMR (300 MHz, $CD_3OD$): $\delta$ 5.09 (d, $J=4.1$ Hz, 1H), 5.08 (d, $J=4.0$ Hz, 1H), 3.94–3.50 (m, 10H), 3.50–3.40 (m, 2H), 3.35–3.25 (m, 1H), 3.19 (app t, $J=9.9$ Hz, 1H), 1.64–1.26 (m, 4H), 0.92 (t, $J=7.3$ Hz, 3H) ppm. $^{13}C$ NMR (75 MHz, $CD_3OD$): $\delta$ 95.1 (CH), 95.0 (CH), 79.7 (CH), 74.7 (CH), 74.5 (CH), 73.8 (CH), 73.6 ($CH_2$), 73.4 (CH), 73.3 (CH), 72.9 (CH), 71.9 (CH), 62.6 ($CH_2$), 62.2 ($CH_2$), 33.6 ($CH_2$), 20.3 ($CH_2$), 14.3 ($CH_3$) ppm. IR (HATR): 3338 (m, br), 2934 (w), 2872 (w), 1455 (w, br), 1361 (w, br), 1258 (w), 1236 (w), 1209 (w), 1145 (m), 1104 (m), 1074 (m), 1040 (m), 990 (s), 939 (w), 909 (w), 842 (w), 801 (w) $cm^{-1}$. $[\alpha]_D^{22}$ +182° (c 0.10, MeOH). ESMS [m/z (fragment, intensity), API-ES positive mode]: 416.2 ($M+NH_4^+$, 100). HRMS (ESI-TOF): calcd. for $C_{16}H_{30}NaO_{11}$ $[M+Na]^+$ 421.1680; found 421.1677.

### *4-O-Isobutyl-α,α-D-trehalose (21-e)*

General procedure C was applied. Purification via column chromatography (gradient elution: $CH_2Cl_2$/MeOH 85/15 to 7/3) afforded the title compound **21-e** as a white solid (77 mg, 0.193 mmol, 100%).

Rf 0.16 in $CH_2Cl_2$/MeOH 8/2. $^1H$ NMR (400 MHz, $CD_3OD$): $\delta$ 5.12–5.07 (m, 2H), 3.93–3.73 (m, 6H), 3.73–3.63 (m, 3H), 3.51–3.43 (m, 2H), 3.35–3.28 (m, 2H), 3.19 (dd, $J=9.9/9.0$ Hz, 1H), 1.83 (app nonaplet, $J=6.6$ Hz, 1H), 0.91 (d, $J=7.0$ Hz, 3H), 0.89 (d, $J=7.0$ Hz, 3H) ppm. $^{13}C$ NMR (100 MHz, $CD_3OD$): $\delta$ 95.0 (CH), 94.9 (CH), 80.7 (CH2), 79.6 (CH), 74.7 (CH), 74.5 (CH), 73.7 (CH), 73.4 (CH), 73.2 (CH), 72.9 (CH), 71.8 (CH), 62.6 ($CH_2$), 62.2 ($CH_2$), 30.2 (CH), 19.9 ($CH_3$), 19.7 ($CH_3$) ppm. IR (HATR): 3326 (br, m), 2951 (w), 2925 (m), 2873 (w), 1458 (w), 1414 (w), 1380 (w), 1365 (w), 1336 (w), 1259 (w), 1204 (w), 1145 (m), 1104 (m), 1031 (m), 983 (s), 951 (m), 841 (w), 802 (w), 732 (w), 706 (w), 613 (w) $cm^{-1}$. $[\alpha]_D^{25}$ +151° (c 0.77, MeOH). ESMS [m/z (fragment, intensity), API-ES positive mode]: 416.2 ($M+NH_4^+$, 100). HRMS (ESI-TOF): calcd. for $C_{16}H_{30}NaO_{11}^+$ $[M+Na]^+$ 421.1680; found 421.1676.

### *4-O-Isopentyl-α,α-D-trehalose (21-f)*

General procedure C was applied. Purification via column chromatography (gradient elution: $CH_2Cl_2$/MeOH 8/2 to 7/3) afforded the title compound **21-f** as a white solid (27.2 mg, 0.066 mmol, 39%).

Rf 0.46 in $CH_3CN/H_2O$ 8/2, 0.36 in $CH_2Cl_2$/MeOH 7/3. $^1H$ NMR (400 MHz, $CD_3OD$): $\delta$ 5.10 (d, $J=3.8$ Hz, 1H), 5.08 (d, $J=3.8$ Hz, 1H), 3.95–3.71 (m, 7H), 3.71–3.57 (m, 3H), 3.51–3.42 (m, 2H), 3.36–3.27 (m, 1H), 3.19 (dd, $J=9.8/9.1$ Hz, 1H), 1.70 (app nonaplet, $J=6.7$ Hz, 1H), 1.56–1.35 (m, 2H), 0.91 (d, $J=6.7$ Hz, 3H), 0.90 (d, $J=6.7$ Hz, 3H) ppm. $^{13}C$ NMR (100 MHz, $CD_3OD$): $\delta$ 95.1 (CH), 95.0 (CH), 79.6 (CH), 74.7 (CH), 74.5 (CH), 73.8 (CH), 73.4 (CH), 73.2 (CH), 72.9 (CH), 72.2 ($CH_2$), 71.9 (CH), 62.6 ($CH_2$), 62.2 ($CH_2$), 40.4 ($CH_2$), 26.0 (CH), 23.2 ($CH_3$), 22.9 ($CH_3$) ppm. IR (HATR): 3327 (br, m), 2953 (w), 2927 (w), 2875 (w), 1461 (w), 1419 (w), 1385 (w), 1366 (w), 1335 (w), 1240 (w), 1204 (w), 1145 (w), 1103 (m), 1069 (m), 1030 (m), 986 (s), 843 (w), 800 (w), 706 (w), 614 (w) $cm^{-1}$. $[\alpha]_D^{25}$ +177° (c 0.09, MeOH). ESMS [m/z (fragment, intensity), API-ES positive mode]: 430.2 ($M+NH_4^+$, 100). HRMS (ESI-TOF): calcd. for $C_{17}H_{32}NaO_{11}^+$ $[M+Na]^+$ 435.1837; found 435.1841.

### *4-O-n-Decyl-α,α-D-trehalose (21-g)*

General procedure C was applied. Purification via column chromatography (gradient elution: $CH_2Cl_2$/MeOH 9/1 to 7/3) afforded the title compound **21-g** as a colourless glass (98 mg, 0.203 mmol, 98%).

Rf 0.23 in $CH_2Cl_2$/MeOH 8/2. $^1H$ NMR (400 MHz, $CD_3OD$): $\delta$ 5.10 (d, $J=3.8$ Hz, 1H), 5.09 (d, $J=3.8$ Hz, 1H), 3.92–3.72 (m, 7H), 3.71–3.52 (m, 3H), 3.51–3.43 (m, 2H), 3.33–3.28 (m, 1H), 3.19 (dd, $J=9.8/9.0$ Hz, 1H), 1.65–1.47 (m, 2H), 1.43–1.20 (m, 14H), 0.89 (t, $J=6.9$ Hz, 3H) ppm. $^{13}C$ NMR (100 MHz, $CD_3OD$): $\delta$ 95.0 (CH), 94.9 (CH), 79.6 (CH), 74.6 (CH), 74.5 (CH), 73.9 ($CH_2$), 73.8 (CH), 73.4 (CH), 73.2 (CH), 72.9 (CH), 71.9 (CH), 62.6 ($CH_2$), 62.2 ($CH_2$), 33.0 ($CH_2$), 31.4 ($CH_2$), 30.8 ($CH_2$), 30.7 ($CH_2$), 30.7 ($CH_2$), 30.4 ($CH_2$), 27.2 ($CH_2$), 23.7 ($CH_2$), 14.4 ($CH_3$) ppm. IR (HATR): 3320 (m, br), 2922 (m), 2854 (w), 1650 (w), 1455 (w), 1370 (w), 1337 (w), 1145 (m), 1101 (m), 1076 (m), 1030 (s), 988 (s), 940 (w), 912 (w), 840 (w), 803 (w), 720 (w), 620 (w) $cm^{-1}$. $[\alpha]_D^{25}$ +142° (c 0.750, MeOH). ESMS [m/z (fragment, intensity), API-ES positive mode]: 500.3 ($M+NH_4^+$, 100). HRMS (ESI-TOF): calcd. for $C_{22}H_{42}NaO_{11}^+$ $[M+Na]^+$ 505.2619; found 505.2620.

### *Lentztrehalose A (2)*

General procedure C was applied. Purification via column chromatography (gradient elution: $CH_2Cl_2$/MeOH 8/2 to 6/4) afforded the title compound **2** as a white solid (68 mg, 0.153 mmol, 100%). Literature data is available [24,30].

Rf 0.17 in $CH_3CN/H_2O$ 8/2, 0.32 in $CH_2Cl_2$/MeOH 6/4. $^1H$ NMR (300 MHz, $CDCl_3$, two diastereoisomers): $\delta$ 5.10 (app d, $J=4.0$ Hz, 1H), 5.08 (app d, $J=3.8$ Hz, 1H), 4.05–3.60 (m, 10H), 3.57–3.41 (m, 3H), 3.35–3.22 (m, 2H), 1.21–1.13 (m, 6H) ppm. $^{13}C$ NMR (75 MHz, $CDCl_3$, only major diastereoisomer visible): $\delta$ 95.1 (CH), 95.0 (CH), 80.4 (CH), 78.2 (CH), 74.7 ($CH_2$), 74.5 (CH), 74.4 (CH), 73.8 (CH), 73.4 (CH), 73.2 (CH), 72.8 (CH), 72.8 (C), 71.9 (CH), 62.6 ($CH_2$), 62.2 ($CH_2$), 26.5 ($CH_3$), 25.4 ($CH_3$) ppm. IR (HATR): 3309 (m, br), 2923 (w), 1362 (w, br), 1229 (w), 1145 (m), 1074 (m), 1033 (m), 985 (s), 934 (m), 855 (w), 843 (w), 800 (w) $cm^{-1}$. $[\alpha]_D^{22}$ +228° (c 0.10, MeOH). ESMS [m/z (fragment, intensity), API-ES positive mode]: 462.2 ($M+NH_4^+$, 100). HRMS (ESI-TOF): calcd. for $C_{17}H_{32}NaO_{13}$ $[M+Na]^+$ 467.1735; found 467.1734.

### *2,3,6,2′,3′-Penta-O-benzyl-4′,6′-O-benzylidene-4-O-(N-n-propylcarbamoyl)-α,α-D-trehalose (22-a)*

General procedure B was applied (use of *n*-propyl isocyanate). Purification via column chromatography (gradient elution: toluene/acetone 99/1 to 95/5) afforded the title compound **22-a** as a colourless oil (372 mg, 0.352 mmol, 68%).

Rf 0.34 in hexane/EtOAc 7/3, 0.19 in toluene/acetone 95/5. $^{1}$H NMR (400 MHz, $CDCl_3$): $\delta$ 7.36–7.09 (m, 35H), 5.26 (d, $J$ = 3.5 Hz, 1H),5.21 (d, $J$ = 3.5 Hz, 1H), 4.98 (d, $J$ = 11.0 Hz, 1H), 4.90–4.35 (m, 15H), 3.41 (d, $J$ = 1.7 Hz, 1H), 3.45–3.37 (m, 2H),0.35 (dd, $J$ = 10.6/5.5 Hz, 1H), 3.08 (q, $J$ = 6.5 Hz, 2H), 1.49–1.37 (m, 2H), 0.86 (t, $J$ = 7.3 Hz, 3H) ppm. $^{13}$C NMR (100 MHz, $CDCl_3$): $\delta$ 155.3 (C), 138.9 (C), 138.7 (C), 138.4 (C), 138.2 (C), 138.1, 138.0 (C), 137.8 (C), 128.3 (CH), 128.2 (CH), 128.1 (CH), 128.0 (CH), 127.9 (CH), 127.8 (CH), 127.7 (CH), 127.7 (CH), 127.6 (CH), 127.5 (CH), 127.5 (CH), 127.4 (CH), 127.4 (CH), 93.6 (CH), 93.5 (CH), 81.8 (CH), 79.2 (CH), 78.9 (CH), 77.7 (CH), 75.5 ($CH_2$), 75.0 ($CH_2$), 74.9 ($CH_2$), 73.6 ($CH_2$), 73.5 ($CH_2$), 73.0 ($CH_2$), 72.4 ($CH_2$), 71.3 (CH), 70.6 (CH), 69.8 (CH), 69.1 ($CH_2$), 68.1 ($CH_2$), 42.8 ($CH_2$), 23.1 ($CH_2$), 11.2 ($CH_3$) ppm. IR (HATR): 3419 (w), 3331 (w), 3060 (w), 3030 (w), 2926 (w), 2870 (w), 1723 (m), 1514 (w), 1496 (m), 1453 (m), 1361 (m), 1332 (w), 1312 (w), 1263 (m), 1231 (m), 1212 (m), 1140 (m), 1097 (s), 1067 (s), 1025 (m), 993 (s), 917 (w), 848 (w), 801 (w), 733 (s), 695 (s) cm$^{-1}$. ESMS [m/z (fragment, intensity), API-ES positive mode]: 1075.4 ($M + NH_4^+$, 42), 518.2 (4-O-n-propylcarbamoyl-2-O,3-O,6-O-tribenzylglucosyl cation, 100). HRMS (ESI-TOF): calcd. for $C_{65}H_{71}NNaO_{12}^+$ $[M + Na]^+$ 1080.4869; found 1080.4847.

### *2,3,6,2′,3′-Penta-O-benzyl-4′,6′-O-benzylidene-4-O-(N-isopropylcarbamoyl)-α,α-D-trehalose (22-b)*

General procedure B was applied (use of isopropyl isocyanate). Purification via column chromatography (gradient elution: hexane/EtOAc 9/1 to 75/25) afforded the title compound **22-b** as a colourless glass (447 mg, 0.422 mmol, 82%).

Rf 0.31 in hexane/EtOAc 7/3. $^{1}$H NMR (400 MHz, $CDCl_3$): $\delta$ 7.38–7.19 (m, 33H), 7.16–7.11 (m, 2H), 5.29 (d, $J$ = 3.3 Hz, 1H), 5.23 (d, $J$ = 3.5 Hz, 1H), 5.00 (d, $J$ = 11.0 Hz, 1 H), 4.87 (dd, $J$ = 11.0/2.4 Hz, 2H), 4.83 (d, $J$ = 10.9 Hz, 1H),) , 4.73 (s, 3H), 4.72–4.61 (m, 4H), 4.56 (d, $J$ = 12.1 Hz, 1H), 4.53–4.36 (m, 2H), 4.50 (d, $J$ = 2.1 Hz, 1H), 4.47 (s, 1H), 4.22–4.14 (m, 2H), 4.05 (t, $J$ = 9.5 Hz, 1H), 3.96 (t, $J$ = 9.5 Hz, 1H), 3.8 (q, $J$ = 6.0 Hz, 1H), 3.69 (t, $J$ = 9.5 Hz, 1H), 3.63 (dt, $J$ = 9.7/3.3 Hz, 2H), 3.55 (dd, $J$ = 10.8/3.2 Hz, 1H), 3.50–3.40 (m, 2H), 3.36 (dd, $J$ = 10.7/5.6 Hz, 1H) 11.12 (s, 3H)) 1.09 (s, 3H) ppm. $^{13}$C NMR (100 MHz, $CDCl_3$): $\delta$ 154.4 (C), 138.9 (C), 138.7 (C), 138.4 (C), 138.2 (C), 139.1 (C), 138.0 (C), 137.8 (C), 128.3 (CH), 128.3 (CH), 128.2 (CH), 128.2 (CH), 128.2 (CH), 128.0 (CH),127.9 (CH), 127.8 (CH), 127.8 (CH), 127.7 (CH), 127.6 (CH), 127.6 (CH), 127.5 (CH), 127.5 (CH), 127.4 (CH), 127.4 (CH), 127.4 (CH), 93.6 (CH), 93.5 (CH), 81.8 (CH), 79.2 (CH), 79.1 (CH), 79.0 (CH), 77.7 (CH), 75.5 ($CH_2$), 75.0 ($CH_2$), 74.9 ($CH_2$), 73.7 ($CH_2$), 73.5 ($CH_2$), 73.0 ($CH_2$), 72.4 ($CH_2$), 71.1 (CH), 70.6 (CH), 69.8 (CH), 69.1 ($CH_2$), 68.1 ($CH_2$), 43.1 (CH), 23.0 ($CH_3$), 22.9 ($CH_3$) ppm. IR (HATR): 3410 (w), 3327 (w), 3059 (w), 3030 (w), 2972 (w), 2923 (w), 2868 (w), 1723 (m), 1496 (m), 1453 (m), 1387 (w), 1362 (m), 1322 (w), 1269 (w), 1230 (m), 1156 (m), 1135 (m), 1097 (m), 1068 (s), 1026 (m), 995 (s), 952 (m), 852 (w), 797 (w), 733 (s), 695 (s), 641 (m) cm$^{-1}$. ESMS [m/z (fragment, intensity), API-ES positive mode]: 1075.4 ($M + NH_4^+$, 42), 518.2 (4-O-isopropylcarbamoyl-2-O,3-O,6-O-tribenzylglucosyl cation, 100). HRMS (ESI-TOF): calcd. for $C_{65}H_{71}NNaO_{12}^+$ $[M + Na]^+$ 1080.4869; found 1080.4847.

### *2,3,6,2′,3′-Penta-O-benzyl-4′,6′-O-benzylidene-4-O-(N-cyclohexylcarbamoyl)-α,α-D-trehalose (22-c)*

General procedure B was applied (use of cyclohexyl isocyanate). Purification via column chromatography (gradient elution: hexane/EtOAc 9/1 to 75/25) afforded the title compound **22-c** as a colourless oil (499 mg, 88%).

Rf 0.31 in hexane/EtOAc 7/3. $^{1}$H NMR (400 MHz, $CDCl_3$): $\delta$ 7.37–7.17 (m, 33H), 7.16–7.10 (m, 2H), 5.29 (d, $J$ = 3.5 Hz, 1H), 5.23 (d, $J$ = 3.5 Hz, 1H), 4.99 (d, $J$ = 10.9 Hz, 1H), 4.91–4.83 (m, 2H), 4.81 (d, $J$ = 10.7 Hz, 1H), 4.77–4.67 (m, 3H), 4.65 (m, 2H), 4.62 (m, 1H), 4.57 (s, 1H), 4.54 (s, 1H), 4.53–3.35 (m, 4H), 4.21–4.14 (m, 2H), 4.04 (t, $J$ = 9.3 Hz, 1H), 3.96 (t, $J$ = 9.5 Hz, 1H), 3.68 (t, $J$ = 9.6 Hz, 1H), 3.62 (dt, $J$ = 9.7/3.7 Hz, 2H), 3.55 (dd, $J$ = 10.7/3.2 Hz, 1H), 3.42 (dt, $J$ = 10.6/2.3 Hz, 3H), 3.36 (dd, $J$ = 10.7/5.6 Hz, 1H), 1.95–1.80 (m, 2H), 1.75–1.60 (m, 3H), 1.42–1.26 (m, 2H), 1.22– 1.00 (m, 3H) ppm. $^{13}$C NMR (100 MHz, $CDCl_3$): $\delta$ 154.4 (C), 138.9 (C), 138.7 (C), 138.4 (C), 138.2 (C), 138.1 (C), 138.0 (C), 137.8 (C), 128.3 (CH), 128.3 (CH), 128.2 (CH), 128.2 (CH), 128.2 (CH), 128.0 (CH), 127.9 (CH), 127.9 (CH), 127.8 (CH), 127.7 (CH), 127.7 (CH), 127.6 (CH), 127.5 (CH), 127.5 (CH), 127.4 (CH), 127.4 (CH), 93.6 (CH), 93.5 (CH), 81.1 (CH), 79.2 (CH), 79.1 (CH), 78.9 (CH), 77.7 (CH), 75.5 ($CH_2$), 75.0 ($CH_2$), 74.9 ($CH_2$), 73.7 ($CH_2$), 73.5 ($CH_2$), 73.0 ($CH_2$), 72.4 ($CH_2$), 71.2 (CH), 70.6 (CH), 69.8 (CH), 69.1 ($CH_2$), 68.1 ($CH_2$), 49.8 (CH), 33.4 ($CH_2$), 33.3 ($CH_2$), 25.5 ($CH_2$), 24.7 ($CH_2$) ppm. IR (HATR): 3410 (w), 3060 (w), 3032 (w), 2928 (w), 2854 (w), 1723 (m), 1496 (m), 1453 (m), 1361 (w), 1315 (w), 1271 (w), 1252 (w), 1212 (m), 1155 (m), 1140 (m), 1097 (s), 1059 (s), 1027 (s), 994 (s), 892 (w), 799 (w), 733 (s), 695 (s) cm$^{-1}$. ESMS [m/z (fragment, intensity), API-ES positive mode]: 1098.4 ($M + H^+$, 10), 558.2 (4-O-cyclohexylcarbamoyl-2-O,3-O,6-O-tribenzylglucosyl cation, 100). HRMS (ESI-TOF): calcd. for $C_{68}H_{75}NNaO_{12}^+$ $[M + Na]^+$ 1120.5181; found 1120.5143.

### *2,3,6,2′,3′-Penta-O-benzyl-4′,6′-O-benzylidene-4-O-(N-phenylcarbamoyl)-α,α-D-trehalose (22-d)*

General procedure B was applied (use of phenyl isocyanate). Purification via column chromatography (gradient elution: toluene/acetone 99/1 to 95/5) afforded the partially purified title compound **22-d** as a colourless oil which was used as such in the next step.

Rf 0.38 in hexane/EtOAc 7/3.

### *2,3,6,2′,3′-Penta-O-benzyl-4′,6′-O-benzylidene-4-O-(N-benzylcarbamoyl)-α,α-D-trehalose (22-e)*

General procedure B was applied (use of benzyl isocyanate). Purification via column chromatography (gradient elution: toluene/acetone 100/0 to 97/3) afforded the title compound **22-e** as a colourless oil (283 mg, 0.256 mmol, 50%).

Rf 0.33 in hexane/EtOAc 7/3. $^{1}$H NMR (400 MHz, $CDCl_3$): $\delta$ 7.36–7.10 (m, 40H), 5.28 (d, $J$ = 3.5 Hz, 1H), 5.23 (d, $J$ = 3.4 Hz, 1H), 5.02–4.91 (m, 2H), 4.86 (d, $J$ = 10.4 Hz, 2H), 4.81–4.75 (m, 2H), 4.75–4.60 (m, 5H), 4.56 (d, $J$ = 12.1 Hz, 1H), 4.48 (q, $J$ = 12.8 Hz, 1H) 4.47 (m, 2H), 4.41 (d, $J$ = 12.1 Hz, 1H), 4.32 (m, 2H), 4.21–4.14 (m, 2H), 4.03 (t, $J$ = 9.3 Hz, 1H), 3.96 (t, $J$ = 9.3 Hz, 1H), 3.69 (t, $J$ = 9.7 Hz, 1H), 3.63 (dd, $J$ = 9.7/3.5, 1H), 3.61 (dd, $J$ = 8.3/3.4 Hz, 1H), 3.55 (dd, $J$ = 10.7/3.3 Hz, 1H), 3.47–3.40 (m, 2H), 3.36 (dd, $J$ = 10.4/5.0 Hz, 1H) ppm. $^{13}$C NMR (100 MHz, $CDCl_3$): $\delta$ 155.3 (C), 138.9 (C), 138.7 (C), 138.4 (C),138.3 (C), 138.2 (C), 138.1 (C), 138.0 (C), 137.8 (C), 128.7 (CH), 128.3 (CH), 128.2 (CH), 128.2 (CH), 128.0 (CH),127.9 (CH), 127.8 (CH), 127.7 (CH), 127.7 (CH), 127.6 (CH), 127.6 (CH), 127.5 (CH), 127.5 (CH), 127.5 (CH), 127.4 (CH), 127.4 (CH), 93.6 (CH), 93.5 (CH), 81.8 (CH), 79.2 (CH), 79.0 (CH), 78.9 (CH),

77.7 (CH), 75.5 ($CH_2$), 75.0 ($CH_2$), 74.9 ($CH_2$), 73.7 ($CH_2$), 73.5 ($CH_2$), 73.0 ($CH_2$), 72.5 ($CH_2$), 71.6 (CH), 70.6 (CH), 68.7 (CH), 69.0 ($CH_2$), 68.1 ($CH_2$), 45.2 ($CH_2$) ppm. IR (HATR): 3410 (w), 3332 (w), 3059 (w), 3031 (w), 2920 (w), 2868 (w), 1724 (m), 1510 (w), 1496 (w), 1453 (w), 1362 (w), 1247 (w), 1208 (w), 1154 (m), 1138 (m), 1097 (m), 1068 (m), 1026 (m), 995 (m), 850 (w), 797 (w), 734 (w), 695 (s), 645 (w) $cm^{-1}$. ESMS [m/z (fragment, intensity), API-ES positive mode]: 1123.4 ($M + NH_4^+$, 95), 566.2 (4-O-benzylcarbamoyl-2-O,3-O,6-O-tribenzylglucosyl cation, 100). HRMS (ESI-TOF): calcd. for $C_{69}H_{71}NNaO_{12}^+$ $[M + Na]^+$ 1128.4868; found 1128.4828.

### *2,3,6,2′,3′-Penta-O-benzyl-4′,6′-O-benzylidene-4-O-[N-(2-phenethyl)-carbamoyl]-α,α-D-trehalose (22-f)*

General procedure B was applied (use of phenethyl isocyanate). Purification via column chromatography (gradient elution: hexane/EtOAc 9/1 to 75/25) afforded the title compound **22-f** as a white foam (130 mg, 0.116 mmol, 57%).

Rf 0.28 in hexane/EtOAc. $^1H$ NMR (400 MHz, $CDCl_3$): $\delta$ 7.35–7.05 (m, 40H), 5.25 (d, $J = 3.3$ Hz, 1H), 5.20 (d, $J = 3.4$ Hz, 1H), 5.00–4.35 (m, 16H), 4.20–4.10 (d, $J = 9.9$ Hz, 2H), 4.01 (t, $J = 7.8$ Hz, 1H), 3.91 (t, $J = 9.4$ Hz, 1H), 3.67 (t, $J = 9.5$ Hz, 1H), 3.62–3.56 (m, 2H), 3.52 (dd, $J = 10.7/3.4$ Hz, 1H), 3.43–3.36 (m, 3H), 3.35–3.30 (m, 1H), 2.79–2.63 (m, 2H) ppm. $^{13}C$ NMR (100 MHz, $CDCl_3$): $\delta$ 155.2 (C), 138.9 (C), 138.7 (C), 138.6 (C), 138.4 (C), 138.2 (C), 138.0 (C), 138.0 (C), 137.8 (C), 128.7 (CH), 128.6 (CH), 128.3 (CH), 128.2 (CH), 128.2 (CH), 128.2 (CH), 128.0 (CH), 127.9 (CH), 127.8 (CH), 127.7 (CH), 127.7 (CH), 127.6 (CH), 127.6 (CH), 127.5 (CH), 127.5 (CH), 127.4 (CH), 127.4 (CH), 127.4 (CH), 126.5 (CH), 93.6 (CH), 93.5 (CH), 81.7 (CH), 79.1 (CH), 78.9 (CH), 77.6 (CH), 75.5 ($CH_2$), 75.0 ($CH_2$), 74.9 ($CH_2$), 73.6 ($CH_2$), 73.5 ($CH_2$), 73.0 ($CH_2$), 72.4 ($CH_2$), 69.0 ($CH_2$), 68.1 ($CH_2$), 42.1 ($CH_2$), 36.0 ($CH_2$) ppm. IR (HATR): 3414 (w), 3336 (w), 3064 (w), 3028 (w), 2922 (w), 2868 (w), 1726 (m), 1496 (m), 1453 (m), 1394 (w), 1362 (m), 1331 (w), 1241 (m), 1208 (w), 1154 (m), 1140 (m), 1098 (s), 1066 (s), 1026 (s), 995 (s), 920 (w), 852 (w), 802 (w), 733 (s), 695 (s), 646 (w) $cm^{-1}$. ESMS [m/z (fragment, intensity), API-ES positive mode]: 1137.4 ($M + NH_4^+$, 28), 580.2 (4-O-phenethylcarbamoyl-2-O,3-O,6-O-tribenzylglucosyl cation, 100). HRMS (ESI-TOF): calcd. for $C_{70}H_{73}NNaO_{12}^+$ $[M + Na]^+$ 1142.5025; found 1142.4978.

### *2,3,6,2′,3′-Penta-O-benzyl-4′,6′-O-benzylidene-4-O-(N-n-octylcarbamoyl)-α,α-D-trehalose (22-g)*

General procedure B was applied (use of *n*-octyl isocyanate). Purification via column chromatography (gradient elution: hexane/EtOAc 95/5 to 75/25) afforded the partially purified title compound **22-g** as a colourless oil which was used as such in the next step.

Rf 0.45 in hexane/EtOAc 7/3.

### *4-O-(N-n-Propylcarbamoyl)-α,α-D-trehalose (23-a)*

General procedure C was applied. Purification via column chromatography (gradient elution: $CH_2Cl_2$/MeOH 10/0 to 7/3) afforded the title compound **23-a** as a white foam (134 mg, 0.314 mmol, 89%).

Rf 0.43 in $CH_3CN/H_2O$ 8/2. $^1H$ NMR (400 MHz, $CD_3OD$): $\delta$ 5.14 (d, $J = 3.8$ Hz, 1H), 5.11 (d, $J = 3.8$ Hz, 1H), 4.53 (t, $J = 9.6$ Hz, 1H), 3.98–3.90 (m, 2H), 3.85–3.74 (m, 3H), 3.67 (dd, $J = 12.0/5.5$ Hz, 1H), 3.62–3.50 (m, 3H), 3.48 (dd, $J = 9.8/3.8$ Hz, 1H), 3.32 (m, 1H), 3.07 (dt, $J = 7.0/1.5$ Hz, 2H), 1.56–1.45 (m, 2H), 0.91 (t, $J = 7.5$ Hz, 3H) ppm. $^{13}C$ NMR (100 MHz, $CD_3OD$): $\delta$ 158.8 (C), 95.1 (CH), 94.8 (CH), 74.5 (CH), 73.8 (CH),73.5 (CH), 73.4 (CH),73.1 (CH), 72.4 (CH), 72.1 (CH), 71.9 (CH), 62.6 ($CH_2$), 62.4 ($CH_2$), 43.7 ($CH_2$), 24.0 ($CH_2$), 11.5 ($CH_3$) ppm. IR (HATR): 3336 (m, br), 2938 (w), 1716 (m), 1522 (m), 1442 (w), 1361 (w), 1317 (w), 1267 (m), 1230 (m), 1144 (m), 1104 (m), 1086 (m), 1047 (m), 1025 (s), 991 (s), 961 (m), 922 (w), 897 (w), 837 (w), 810 (w), 766 (w), 720 (w) $cm^{-1}$. $[\alpha]_D^{22}$ +162° (c 0.10, MeOH). ESMS [m/z (fragment, intensity), API-ES positive mode]: 428.1 ($M + H^+$, 100).

### *4-O-(N-Isopropylcarbamoyl)-α,α-D-trehalose (23-b)*

General procedure C was applied. Purification via column chromatography (gradient elution: $CH_2Cl_2$/MeOH 9/1 to 6/4) afforded the title compound **23-b** as a white foam (129 mg, 0.302 mmol, 76%).

Rf 0.59 in $CH_3CN/H_2O$ 8/2, 0.12 in $CH_2Cl_2$/MeOH 8/2. $^1H$ NMR (400 MHz, $CD_3OD$): $\delta$ 5.14 (d, $J = 3.8$ Hz, 1H), 5.11 (d, $J = 3.8$ Hz, 1H), 4.53 (dd, $J = 10.4/9.5$ Hz, 1H), 3.97–3.89 (m, 2H), 3.85–3.78 (m, 1H), 3.77 (m, 1H), 3.76–3.62 (m, 2H), 3.60–3.53 (m, 2H), 3.48 (dd, $J = 9.8/3.8$ Hz, 1H), 3.31 (d, $J = 9.5$ Hz, 1H), 11.14 (s, 3H), 1.14 (s, 3H) ppm. $^{13}C$ NMR (100 MHz, $CD_3OD$): $\delta$ 157.9 (C), 95.0 (CH), 94.8 (CH), 74.5 (CH), 73.8 (CH), 73.4 (CH), 73.1 (CH), 72.4 (CH), 72.1 (CH), 71.9 (CH), 62.6 ($CH_2$), 62.4 ($CH_2$), 44.2 (CH), 22.8 (CH) ppm. IR (HATR): 3301 (m, br), 2936 (w), 1694 (m), 1540 (w), 1457 (w), 1325 (w), 1256 (m), 1138 (m), 1108 (m), 1074 (m), 1031 (s), 992 (s), 946 (m), 914 (w), 841 (w), 806 (w) $cm^{-1}$. $[\alpha]_D^{22}$ +190° (c 0.10, MeOH). ESMS [m/z (fragment, intensity), API-ES positive mode]: 428.2 ($M + H^+$, 100).

### *4-O-(N-Cyclohexylcarbamoyl)-α,α-D-trehalose (23-c)*

General procedure C was applied. Purification via column chromatography (gradient elution: $CH_2Cl_2$/MeOH 9/1 to 6/4) afforded the title compound **23-c** as a white foam (116 mg, 0.248 mmol, 58%).

Rf 0.54 in $CH_3CN/H_2O$ 8/2, 0.59 in $CH_2Cl_2$/MeOH 6/4. $^1H$ NMR (300 MHz, $D_2O$): $\delta$ 5.20 (d, $J = 4.5$ Hz, 1H), 5.16 (d, $J = 3.8$ Hz, 1H), 4.56 (t, $J = 9.6$ Hz, 1H), 3.99 (t, $J = 9.8$ Hz, 1H), 3.86–3.55 (m, 9H), 3.42 (t, $J = 9.2$ Hz, 1H), 3.4 (s, 1H), 1.86–1.76 (m, 2H), 1.73–1.64 (m, 2H), 1.60–1.50 (m, 1H), 1.35–1.05 (m, 5H) ppm. $^{13}C$ NMR (75 MHz, $D_2O$): $\delta$ 157.6 (C), 94.0 (CH), 93.8 (CH), 73.2 (CH), 72.9 (CH), 72.1 (CH), 71.7 (CH), 71.5 (CH), 71.3 (CH), 71.1 (CH), 70.3 (CH), 61.2 ($CH_2$), 61.0 ($CH_2$), 50.8 (CH), 33.0 ($CH_2$), 25.6 ($CH_2$), 25.0 ($CH_2$) ppm. IR (HATR): 3244 (m, br), 2943 (m), 2857 (w), 1678 (s), 1465 (w), 1447 (w), 1409 (m), 1374 (w), 1347 (w), 1329 (w), 1265 (w), 1133 (m), 1102 (m), 1076 (m), 1046 (s), 1037 (s), 1018 (s), 993 (s), 953 (m), 929 (w), 890 (w), 868 (w), 805 (w), 789 (w), 720 (w), 703 (w) $cm^{-1}$. $[\alpha]_D^{22}$ +171° (c 0.10, MeOH). ESMS [m/z (fragment, intensity), API-ES positive mode]: 468.2 ($M + H^+$, 55), 288.1 (M-glucose + $H^+$, 100).

### *4-O-(N-Phenylcarbamoyl)-α,α-D-trehalose (23-d)*

General procedure C was applied. Purification via column chromatography (gradient elution: $CH_2Cl_2$/MeOH 10/0 to 7/3) afforded the title compound **23-d** as a white foam (55 mg, 0.119 mmol, 16% over two steps).

Rf 0.46 in $CH_3CN/H_2O$ 8/2. $^1H$ NMR (400 MHz, $D_2O$): $\delta$ 4.41–7.35 (m, 4H), 7.20–7.14 (m, 1H), 5.22 (d, $J = 3.9$ Hz, 1H), 5.18 (d, $J = 3.8$ Hz, 1H), 4.72 (d, $J = 9.9$ Hz, 1H), 4.08 (t, $J = 9.8$ Hz, 1H), 4.06–3.97 (m, 1H), 3.87–3.79 (m, 3H), 3.78–3.70 (m, 3H), 3.68– 3.60 (m, 2H), 3.43 (t, $J = 9.5$ Hz, 1H) ppm. $^{13}C$ NMR (100 MHz, $D_2O$): $\delta$ ): 155.7 (C), 137.9 (C), 130.0 (CH), 125.1 (CH), 120.8 (CH), 94.1 (CH), 93.9 (CH), 73.2 (CH), 72.9 (CH), 72.4 (CH), 71.7 (CH), 71.5 (CH), 71.3 (CH), 70.9 (CH), 70.3 (CH), 61.2 ($CH_2$), 60.9 ($CH_2$) ppm. IR (HATR): 3279 (m, br), 2932 (w), 1702 (m), 1601 (m), 1542 (m), 1501 (w),

1445 (m), 1318 (m), 1231 (m), 1143 (w), 1108 (m), 1086 (m), 1027 (s), 992 (s), 939 (m), 914 (w), 838 (w), 805 (w), 755 (w), 723 (w), 690 (w) $cm^{-1}$. ESMS [m/z (fragment, intensity), API-ES positive mode]: 282.1 (M-glucose + $H^+$, 100).

### *4-O-(N-Benzylcarbamoyl)-α,α-D-trehalose (23-e)*

General procedure C was applied. Purification via column chromatography (gradient elution: $CH_2Cl_2$/MeOH 10/0 to 7/3) afforded the title compound **23-e** as a white foam (99 mg, 0.208 mmol, 87%).

Rf 0.44 in $CH_3CN/H_2O$ 8/2. $^1H$ NMR (400 MHz, $CD_3OD$): $\delta$ 7.32–7.27 (m, 4H), 7.25–7.19 (m, 1H), 5.14 (d, $J$ = 3.8 Hz, 1H), 5.11 (d, $J$ = 3.7 Hz, 1H), 4.58 (t, $J$ = 9.8 Hz, 1H),4.30 (s, 2H), 4.00–3.91 (m, 2H), 3.84–3.74 (m, 3H), 3.70–3.45 (m, 5H), 3.32 (m, 1H) ppm. $^{13}C$ NMR (100 MHz, $CD_3OD$): $\delta$ 158.9 (C), 140.5 (C), 129.5 (CH), 128.3 (CH), 128.2 (CH), 95.1 (CH), 94.8 (CH), 74.5 (CH), 73.8 (CH), 73.8 (CH), 73.4 (CH), 73.1 (CH), 72.1 (CH), 71.9 (CH), 62.6 ($CH_2$), 62.4 ($CH_2$), 45.6 ($CH_2$) ppm. IR (HATR): 3311 (m, br), 2931 (w), 1698 (m), 1539 (w), 1454 (w), 1339 (w), 1254 (m), 1207 (w), 1145 (m), 1104 (w), 1080 (m), 1028 (m), 989 (s), 944 (m), 841 (w), 802 (w), 747 (w), 720 (w), 698 (m) $cm^{-1}$. $[\alpha]_D^{22}$ +142° (c 0.10, MeOH). ESMS [m/z (fragment, intensity), API-ES positive mode]: 476.2 (M + $H^+$, 100).

### *4-O-[N-(2-Phenethyl)carbamoyl]-α,α-D-trehalose (23-f)*

General procedure C was applied. Purification via column chromatography (gradient elution: $CH_2Cl_2$/MeOH 9/1 to 7/3) afforded the title compound **23-f** as a white foam (112 mg, 0.229 mmol, 75%).

Rf 0.47 in $CH_3CN/H_2O$ 8/2. $^1H$ NMR (400 MHz, $CD_3OD$): $\delta$ 7.30–7.14 (m, 5H), 5.14 (d, $J$ = 3.8 Hz, 1H), 5.10 (d, $J$ = 3.8 Hz, 1H), 4.57 (s, 1H), 4.58 (t, $J$ = 9.6 Hz, 1H), 3.96–3.90 (m, 2H), 3.85–3.75 (m, 3H), 3.67 (dd, $J$ = 12.0/6.5 Hz, 1H), 3.56 (dd, $J$ = 9.9/3.8 Hz, 2H), 3.35–3.45 (m, 2H), 3.40–3.27 (m, 2H), 2.85–2.72 (m, 2H) ppm. $^{13}C$ NMR (100 MHz, $CD_3OD$): $\delta$ 158.7 (C), 140.5 (C), 129.9 (CH), 129.5 (CH), 127.4 (CH), 95.1 (CH), 94.8 (CH), 74.5 (CH), 73.9 (CH), 73.6 (CH), 73.4 (CH), 73.1 (CH), 72.4 (CH), 72.0 (CH), 71.9 (CH), 62.6 ($CH_2$), 62.4 ($CH_2$), 43.5 ($CH_2$), 37.0 ($CH_2$) ppm. IR (HATR): 3320 (m, br), 2931 (w), 1699 (m), 1540 (m), 1452 (w), 1339 (w), 1251 (m), 1202 (w), 1148 (m), 1106 (w), 1080 (m), 1030 (m), 989 (s), 846 (w), 806 (w), 748 (w), 698 (m) $cm^{-1}$. $[\alpha]_D^{22}$ +132° (c 0.10, MeOH). ESMS [m/z (fragment, intensity), API-ES positive mode]: 490.2 (M + $H^+$, 20), 310.0 (M-glucose + $H^+$, 100).

### *4-O-(N-n-Octylcarbamoyl)-α,α-D-trehalose (23-g)*

General procedure C was applied. Purification via column chromatography (gradient elution: $CH_2Cl_2$/MeOH 10/0 to 7/3) afforded the title compound **23-g** as a white foam (154 mg, 0.310 mmol, 74%).

Rf 0.27 in $CH_3CN/H_2O$ 8/2. $^1H$ NMR (400 MHz, $CD_3OD$): $\delta$ 5.14 (d, $J$ = 3.9 Hz, 1H), 5.10 (d, $J$ = 3.7 Hz, 1H), 4.53 (t, $J$ = 9.8 Hz, 1H), 3.98 (m, 2H), 3.84–3.74 (m, 2H), 3.66 (dd, $J$ = 12.0/5.5 Hz, 1H), 3.62–3.50 (m, 3H), 3.47 (dd, $J$ = 9.6/3.7 Hz, 1H), 3.31 (m, 1H), 3.10 (t, $J$ = 7.0 Hz, 2H), 3.09 (t, $J$ = 7.2 Hz, 2H), 1–55-1.45 (m, 2H), 1.35–1.25 (m, 10H), 0.89 (t, $J$ = 6.7 Hz, 3H) ppm. $^{13}C$ NMR (100 MHz, $CD_3OD$): $\delta$ 158.8 (C), 95.1 (CH), 94.8 (CH), 74.5 (CH), 73.8 (CH), 73.5 (CH), 73.4 (CH), 73.1 (CH), 72.4 (CH), 72.1 (CH), 71.9 (CH), 62.6 ($CH_2$), 62.4 ($CH_2$), 41.9 ($CH_2$), 33.0 ($CH_2$), 30.9 ($CH_2$), 30.4 ($CH_2$), 30.4 ($CH_2$), 27.9 ($CH_2$), 23.7 ($CH_2$), 14.4 (CH) ppm. IR (HATR): 3320 (m, br), 2928 (m), 2854 (w), 1697 (m), 1534 (m), 1456 (w), 1376 (w), 1257 (m), 1148 (m), 1104 (m), 1080 (m), 1027 (m), 992 (s), 944 (m), 846 (w), 804 (w) $cm^{-1}$. $[\alpha]_D^{22}$ +157° (c 0.12, MeOH). ESMS [m/z (fragment, intensity), API-ES negative mode]: 556.2 (M + $OAc^-$, 100).

### *2,3,4,2′,3′,4′-Hexa-O-benzyl-6,6′-di-O-methyl-α,α-D-trehalose (24)*

General procedure A was applied (use of iodomethane). Purification via column chromatography (eluent: hexane/EtOAc 7/3) afforded the title compound **24** as a colourless glass (482 mg, 0.529 mmol, 99%).

Rf 0.18 in hexane/EtOAc 7/3. $^1H$ NMR (300 MHz, $CDCl_3$): $\delta$ 7.40–7.22 (m, 30H), 5.19 (d, $J$ = 3.6 Hz, 2H), 4.99 (d, $J$ = 10.9 Hz, 2H), 4.87 (d, $J$ = 11.1 Hz, 2H), 4.85 (d, $J$ = 10.9 Hz, 2H), 4.70 (d, $J$ = 12.4 Hz, 2H, A-part of AB-system), 4.66 (d, $J$ = 12.2 Hz, 2H, B-part of AB-system), 4.13 (app dt, $J$ = 10.0/2.1 Hz, 2H), 4.02 (app t, $J$ = 9.3 Hz, 2H), 3.64 (app t, $J$ = 9.6 Hz, 2H), 3.56 (dd, $J$ = 9.7/3.7 Hz, 2H), 3.42 (dd, $J$ = 10.6/2.8 Hz, 2H), 3.32–3.24 (m, 2H), 3.26 (s, 6H) ppm. $^{13}C$ NMR (100 MHz, $CDCl_3$): $\delta$ 138.9 (C), 138.4 (C), 138.2 (C), 128.4 (CH), 128.3 (CH), 128.0 (CH), 127.8 (CH), 127.7 (CH), 127.5 (CH), 127.5 (CH), 94.7 (CH), 81.7 (CH), 79.3 (CH), 77.6 (CH), 75.5 ($CH_2$), 75.1 ($CH_2$), 72.7 ($CH_2$), 70.6 ($CH_2$), 70.5 (CH), 59.1 ($CH_3$) ppm. IR (HATR): 3027 (w), 2918 (w), 2882 (w), 1727 (w), 1496 (w), 1453 (m), 1359 (m), 1326 (w), 1267 (w), 1198 (m), 1149 (m), 1135 (m), 1090 (s), 1067 (s), 1027 (m), 992 (s), 915 (m), 848 (w), 802 (w), 734 (m), 695 (s), 642 (w) $cm^{-1}$. ESMS [m/z (fragment, intensity), API-ES positive mode]: 928.4 (M + $NH_4^+$, 100). HRMS (ESI-TOF): calcd. for $C_{56}H_{66}NO_{11}^+$ $[M + NH_4]^+$ 928.4630; found 928.4626.

### *6,6′-Di-O-methyl-α,α-D-trehalose (25)*

General procedure C was applied. Purification via column chromatography (gradient elution: $CH_2Cl_2$/MeOH 9/1 to 7/3) afforded the title compound **25** as a white solid (164 mg, 0.443 mmol, 92%).

Rf 0.31 in $CH_3CN/H_2O$ 8/2, 0.29 in $CH_2Cl_2$/MeOH 7/3. $^1H$ NMR (400 MHz, $CD_3OD$): $\delta$ 5.05 (d, $J$ = 3.7 Hz, 2H), 3.94 (ddd, $J$ = 10.0/4.7/2.6 Hz, 2H), 3.76 (app t, $J$ = 9.3 Hz, 2H), 3.66–3.53 (m, 4H), 3.45 (dd, $J$ = 9.8/3.8 Hz, 2H), 3.37 (s, 6H), 3.34–3.27 (m, 2H) ppm. $^{13}C$ NMR (100 MHz, $CD_3OD$): $\delta$ 95.3 (CH), 74.5 (CH), 73.1 (CH), 73.0 ($CH_2$), 72.5 (CH), 72.0 (CH), 59.5 ($CH_3$) ppm. IR (HATR): 3300 (m, br), 2929 (w), 1454 (w), 1379 (w), 1334 (w), 1275 (w), 1241 (w), 1195 (w), 1148 (m), 1108 (m), 1079 (m), 1038 (m), 1015 (m), 986 (s), 944 (m), 911 (m), 856 (w), 808 (w), 688 (w) $cm^{-1}$. $[\alpha]_D^{22}$ +190° (c 0.10, MeOH). ESMS [m/z (fragment, intensity), API-ES positive mode]: 388.2 (M + $NH_4^+$, 100). HRMS (ESI-TOF): calcd. for $C_{14}H_{26}NaO_{11}^+$ $[M + Na]^+$ 393.1367; found 393.1360.

### *2,3,4,2′,3′,4′-Hexa-O-benzyl-6,6′-di-O-[N-(2-phenethyl)carbamoyl]-α,α-D-trehalose (26)*

General procedure B was applied (use of phenethyl isocyanate). Purification via column chromatography (gradient elution: hexane/EtOAc 85/15 to 6/4) afforded the partially purified title compound **26** as a colourless glass which is used as such in the next step.

Rf 0.11 in hexane/EtOAc 7/3.

### *6,6′-Di-O-[N-(2-phenethyl)carbamoyl]-α,α-D-trehalose (27)*

General procedure C was applied. Purification via column chromatography (gradient elution: $CH_2Cl_2$/MeOH 9/1 to 6/4) afforded the title compound **27** as a white solid (178 mg, 0.280 mmol, 65% over 2 steps).

Rf 0.52 in $CH_3CN/H_2O$ 8/2, 0.34 in $CH_2Cl_2$/MeOH 8/2. $^1H$ NMR (400 MHz, $CD_3OD$): $\delta$ 7.30–7.10 (m, 10H), 5.13–4.99 (m, 2H), 4.37–4.14 (m, 4H), 4.09–3.92 (m, 2H), 3.77 (t, $J$ = 9.3 Hz, 2H), 3.44 (br dd, $J$ = 9.6/3.4 Hz, 2H), 3.37–3.23 (m, 6H), 2.76 (t, $J$ = 7.4 Hz, 4H)

ppm. $^{13}$C NMR (100 MHz, $CD_3OD$): $\delta$ 159.1 (C), 140.5 (C), 129.8 (CH), 129.5 (CH), 127.3 (CH), 95.3 (CH), 74.5 (CH), 73.2 (CH), 71.9 (CH), 71.8 (CH), 64.9 ($CH_2$), 43.5 ($CH_2$), 37.1 ($CH_2$) ppm. IR (HATR): 3342 (m, br), 2934 (w), 1697 (s), 1520 (m), 1496 (w), 1454 (w), 1347 (w), 1327 (w), 1240 (m), 1204 (w), 1148 (m), 1099 (m), 1074 (m), 1040 (m), 1015 (m), 984 (s), 942 (m), 909 (w), 853 (w), 808 (w), 772 (w), 749 (w), 698 (m) $cm^{-1}$. $[\alpha]_D^{22}$ +110° (c 0.10, MeOH). ESMS [m/z (fragment, intensity), API-ES negative mode]: 637.2 ($M+H^+$, 100). HRMS (ESI-TOF): calcd. for $C_{30}H_{40}N_2NaO_{13}$ $[M+Na]^+$ 659.2423; found 629.2414.

### *2,3,6,2′,3′,6′-Hexa-O-benzyl-4,4′-di-O-methyl-α,α-D-trehalose (28)*

General procedure A was applied (use of iodomethane). Purification via column chromatography (eluent: hexane/EtOAc 8/2) afforded the title compound **28** as a colourless glass (302 mg, 0.331 mmol, 98%).

Rf 0.21 in hexane/EtOAc 8/2. $^1$H NMR (400 MHz, $CDCl_3$): $\delta$ 7.43–7.22 (m, 30H), 5.19 (d, $J = 3.5$ Hz, 2H), 4.97 (d, $J = 10.9$ Hz, 2H), 4.86 (d, $J = 10.9$ Hz, 2H), 4.69 (d, $J = 12.1$ Hz, 2H, A-part of AB-system), 4.67 (d, $J = 12.2$ Hz, 2H, B-part of AB-system), 4.58 (d, $J = 12.1$ Hz, 2H), 4.43 (d, $J = 12.1$ Hz, 2H), 4.09 (app dt, $J = 10.1/2.3$ Hz, 2H), 3.94 (app t, $J = 9.3$ Hz, 2H), 3.57–3.36 (m, 8H), 3.47 (s, 6H) ppm. $^{13}$C NMR (100 MHz, $CDCl_3$): $\delta$ 138.9 (C), 138.2 (C), 138.0 (C), 128.3 (CH), 128.3 (CH), 128.3 (CH), 127.9 (CH), 127.8 (CH), 127.6 (CH), 127.5 (CH), 127.4 (CH), 94.6 (CH), 81.7 (CH), 79.3 (CH), 79.2 (CH), 75.5 ($CH_2$), 73.4 ($CH_2$), 72.7 ($CH_2$), 70.6 (CH), 68.2 ($CH_2$), 60.6 ($CH_3$) ppm. IR (HATR): 3064 (w), 3028 (w), 2930 (w), 2859 (w), 1496 (w), 1453 (m), 1364 (m), 1331 (w), 1207 (w), 1155 (m), 1135 (m), 1090 (s), 1024 (m), 993 (s), 913 (m), 854 (w), 797 (w), 732 (s), 695 (s), 636 (w) $cm^{-1}$. ESMS [m/z (fragment, intensity), API-ES positive mode]: 928.4 ($M+NH_4^+$, 100). HRMS (ESI-TOF): calcd. for $C_{56}H_{66}NO_{11}^+$ $[M+NH_4]^+$ 928.4630; found 928.4639.

### *4,4′-Di-O-methyl-α,α-D-trehalose (29)*

General procedure C was applied. Purification via column chromatography (gradient elution: $CH_2Cl_2$/MeOH 9/1 to 7/3) afforded the title compound **29** as a white solid (99 mg, 0.267 mmol, 86%).

Rf 0.28 in $CH_3CN/H_2O$ 8/2, 0.31 in $CH_2Cl_2$/MeOH 7/3. $^1$H NMR (400 MHz, $CD_3OD$): $\delta$ 5.06 (d, $J = 3.8$ Hz, 2H), 3.85 (app t, $J = 9.4$ Hz, 2H), 3.79 (ddd, $J = 10.0/4.3/2.0$ Hz, 2H), 3.74 (dd, $J = 11.8/2.1$ Hz, 2H), 3.65 (dd, $J = 11.9/4.5$ Hz, 2H), 3.55 (s, 6H), 3.45 (dd, $J = 9.8/3.8$ Hz, 2H), 3.11 (dd, $J = 9.7/9.2$ Hz, 2H) ppm. $^{13}$C NMR (100 MHz, $CD_3OD$): $\delta$ 95.0 (CH), 81.1 (CH), 74.5 (CH), 73.3 (CH), 72.8 (CH), 62.1 ($CH_2$), 60.8 ($CH_3$) ppm. IR (HATR): 3380 (m, br), 2932 (w), 1660 (w), 1472 (w), 1442 (w), 1420 (w), 1389 (w), 1339 (w), 1317 (w), 1244 (w), 1229 (w), 1209 (w), 1192 (w), 1156 (m), 1135 (m), 1101 (m), 1063 (s), 1029 (m), 992 (s), 963 (s), 928 (m), 854 (m), 804 (w), 702 (w), 655 (w) $cm^{-1}$. $[\alpha]_D^{22}$ +220° (c 0.10, MeOH). ESMS [m/z (fragment, intensity), API-ES positive mode]: 393.1 ($M+Na^+$, 41), 388.2 ($M+NH_4^+$, 100). HRMS (ESI-TOF): calcd. for $C_{14}H_{26}NaO_{11}^+$ $[M+Na]^+$ 393.1367; found 393.1361.

### *2,3,2′,3′,4′,6′-Hexa-O-benzyl-4,6′-di-O-methyl-α,α-D-trehalose (30)*

General procedure A was applied (use of iodomethane). Purification via column chromatography (gradient elution: hexane/EtOAc 85/15 to 75/25) afforded the title compound **30** as a colourless oil (131 mg, 0.144 mmol, 85%).

Rf 0.12 in hexane/EtOAc 8/2. $^1$H NMR (400 MHz, $CDCl_3$): $\delta$ 7.40–7.18 (m, 28H), 7.16–7.08 (m, 2H), 5.20 (d, $J = 3.5$ Hz, 1H), 5.17 (d, $J = 3.7$ Hz, 1H), 4.99 (d, $J = 11.0$ Hz, 1H), 4.95 (d, $J = 11.0$ Hz, 1H), 4.85 (app d, $J = 10.9$ Hz, 2H), 4.80 (d, $J = 10.7$ Hz, 1H), 4.71–4.60 (m, 4H), 4.54 (d, $J = 12.1$ Hz, 1H), 4.45 (d, $J = 10.8$ Hz, 1H), 4.36 (d, $J = 12.2$ Hz, 1H), 4.16 (dt, $J = 9.9/2.2$ Hz, 1H), 4.05 (dt, $J = 10.1/2.4$ Hz, 1H), 4.02 (app t, $J = 9.5$ Hz, 1H), 3.91 (app t, $J = 9.3$ Hz, 1H), 3.68 (app br t, $J = 9.6$ Hz, 1H), 3.59 (dd, $J = 9.6/3.5$ Hz, 1H), 3.54–3.28 (m, 6H), 3.53 (s, 3H), 3.32 (s, 3H) ppm. $^{13}$C NMR (100 MHz, $CDCl_3$): $\delta$ 139.0 (C), 138.9 (C), 138.4 (C), 138.3 (C), 138.2 (C), 137.8 (C), 128.4 (CH), 128.3 (CH), 128.3 (CH), 128.3 (CH), 128.0 (CH), 128.0 (CH), 127.9 (CH), 127.8 (CH), 127.7 (CH), 127.6 (CH), 127.5 (CH), 127.4 (CH), 127.4 (CH), 94.7 (CH), 94.7 (CH), 81.8 (CH), 81.5 (CH), 79.4 (CH), 79.3 (CH), 79.1 (CH), 77.6 (CH), 75.6 ($CH_2$), 75.4 ($CH_2$), 75.1 ($CH_2$), 73.5 ($CH_2$), 72.7 ($CH_2$), 70.7 ($CH_2$), 70.6 (CH), 70.5 (CH), 68.1 ($CH_2$), 60.5 ($CH_3$), 59.1 ($CH_3$) ppm. IR (HATR): 3059 (w), 3028 (w), 2915 (w), 2868 (w), 1496 (w), 1453 (m), 1363 (m), 1328 (w), 1265 (w), 1199 (w), 1153 (m), 1140 (m), 1087 (s), 1069 (s), 1026 (m), 992 (s), 920 (m), 853 (w), 799 (w), 733 (s), 695 (s), 638 (w) $cm^{-1}$. ESMS [m/z (fragment, intensity), API-ES positive mode]: 928.4 ($M+NH_4^+$, 100). HRMS (ESI-TOF): calcd. for $C_{56}H_{66}NO_{11}^+$ $[M+NH_4]^+$ 928.4630; found 928.4645.

### *4,6-Di-O-methyl-α,α-D-trehalose (31)*

General procedure C was applied. Purification via column chromatography (gradient elution: $CH_2Cl_2$/MeOH 9/1 to 7/3) afforded the title compound **31** as a white glass (42.8 mg, 0.116 mmol, 87%).

Rf 0.29 in $CH_3CN/H_2O$ 8/2, 0.16 in $CH_2Cl_2$/MeOH 8/2. $^1$H NMR (400 MHz, $CD_3OD$): $\delta$ 5.07 (d, $J = 3.8$ Hz, 1H), 5.05 (d, $J = 3.8$ Hz, 1H), 3.91 (ddd, $J = 10.0/4.2/2.1$ Hz, 1H), 3.85 (app t, $J = 9.4$ Hz, 1H), 3.82–3.72 (m, 3H), 3.66 (dd, $J = 12.0/5.5$ Hz, 1H), 3.62–3.52 (m, 2H), 3.53 (s, 3H), 3.47 (dd, $J = 9.8/3.8$ Hz, 1H), 3.45 (dd, 9.8/3.7 Hz, 1H), 3.38 (s, 3H), 3.35–3.27 (m, 1H), 3.09 (dd, $J = 10.0/9.0$ Hz, 1H) ppm. $^{13}$C NMR (100 MHz, $CD_3OD$): $\delta$ 95.2 (CH), 95.0 (CH), 81.3 (CH), 74.5 (CH), 74.4 (CH), 73.8 (CH), 73.3 (CH), 73.2 (CH), 72.6 ($CH_2$), 71.8 (CH), 71.5 (CH), 62.6 ($CH_2$), 60.8 ($CH_3$), 59.4 ($CH_3$) ppm. IR (HATR): 3338 (m, br), 2922 (w), 1373 (m, br), 1192 (w), 1069 (m), 1032 (m), 989 (s), 968 (m), 936 (m), 924 (m), 912 (m), 802 (m), 681 (w) $cm^{-1}$. $[\alpha]_D^{22}$ +203° (c 0.10, MeOH). ESMS [m/z (fragment, intensity), API-ES positive mode]: 388.2 ($M+NH_4^+$, 100). HRMS (ESI-TOF): calcd. for $C_{14}H_{26}NaO_{11}$ $[M+Na]^+$ 393.1367; found 393.1359.

### *2,3,4,2′,3′,4′-Hexa-O-benzyl-6-O-methyl-α,α-D-trehalose (32)*

To a cooled (0 °C) solution of **16-a** (420 mg, 0.469 mmol, 1 eq) in toluene (5 ml), DIBAL-H (supplied as 1.0 M solution in toluene, 2.35 ml, 2.35 mmol, 5 eq) was added dropwise. After 15 min, the cooling bath was removed and the reaction mixture was stirred for 48 h. The resulting solution was cooled to 0 °C after which MeOH (1.3 ml) and 10% aqueous KOH (0.4 ml) were added. The mixture was transferred to a separation funnel using $H_2O$ (35 ml) and dichloromethane (35 ml). The organic layer was separated and the aqueous layer was extracted with dichloromethane (3 × 35 ml). The combined organic layers were washed with brine (50 ml) and concentrated under reduced pressure. The residue was purified via column chromatography (gradient elution: hexane/EtOAc 7/3 to 6/4), giving the title compound **32** as a colourless glass in 76% yield (277 mg, 0.3088 mmol) based on recovered starting material (55 mg).

Rf 0.12 in hexane/EtOAc 6/4. $^1$H NMR (400 MHz, $CDCl_3$): $\delta$ 7.40–7.23 (m, 30H), 5.18 (d, $J = 3.5$ Hz, 1H), 5.15 (d, $J = 3.7$ Hz, 1H), 5.00 (d, $J = 10.9$ Hz, 1H), 4.99 (d, $J = 10.9$ Hz, 1H), 4.92–4.83 (m, 4H), 4.76–4.62 (m, 5H), 4.58 (d, $J = 11.0$ Hz, 1H), 4.18–3.98 (m, 4H), 3.69–3.49 (m, 6H), 3.42 (br dd, $J = 10.6/3.1$ Hz, 1H), 3.31 (br dd, $J = 10.6/2.0$ Hz, 1H), 3.27 (s, 3H), 1.46 (br t, $J = 6.0$ Hz, 1H) ppm. $^{13}$C NMR (100 MHz, $CDCl_3$): $\delta$ 138.9 (C), 138.8 (C), 138.4 (C), 138.2 (C), 138.2 (C), 138.1 (C), 128.5 (CH), 128.4 (CH), 128.4 (CH), 128.4 (CH),

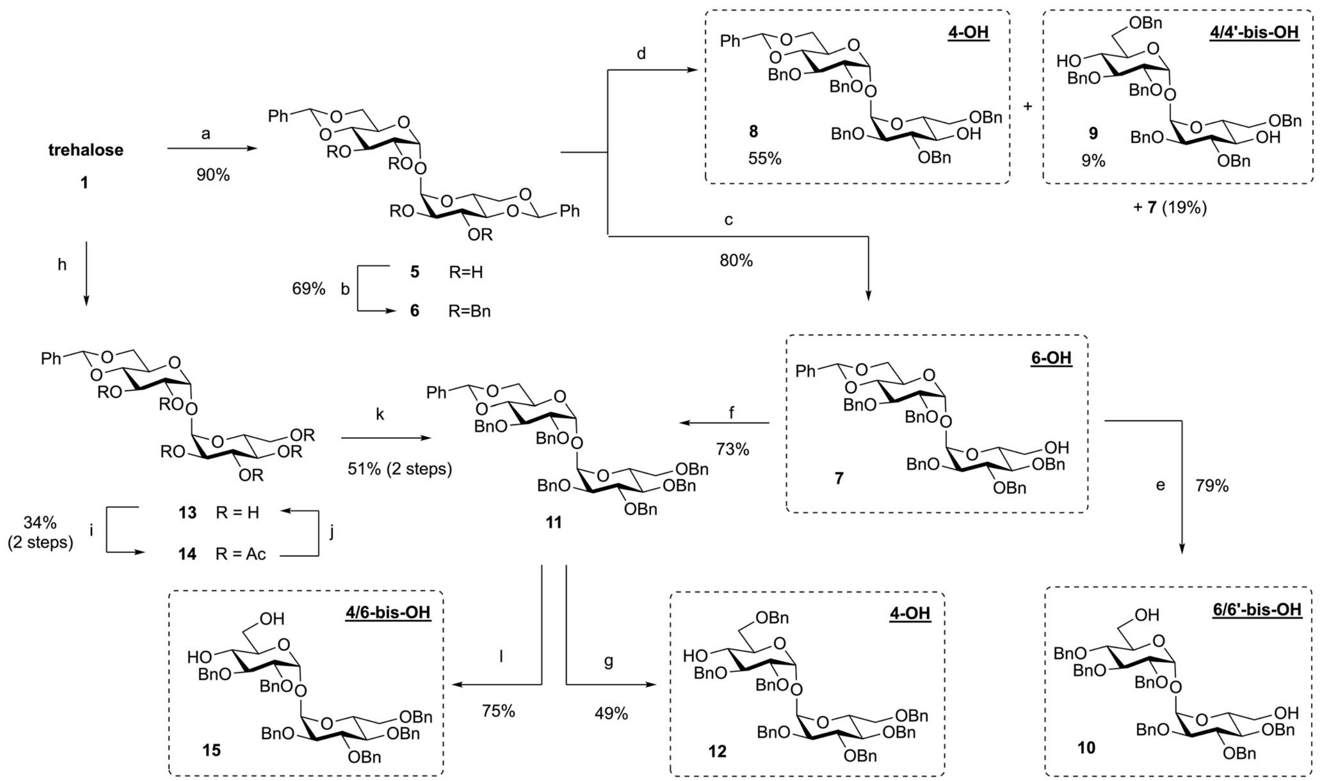

Scheme 1. Synthesis of strategic intermediates. Reagents and conditions: (a) 2.3 eq benzaldehyde dimethyl acetal, 0.05 eq camphorsulfonic acid, DMF, 95 °C, 30 min; (b) 10 eq NaH, 8 eq BnBr, 0.2 eq TBAI, DMF, 0 °C-rt, 18 h; (c) 5 eq DIBAL-H, toluene, −18 °C-rt, 2 h; (d) 5 eq DIBAL-H, $CH_2Cl_2$, 0 °C-rt, 90 min; (e) 5 eq DIBAL-H, toluene, 48 h; (f) 2.5 eq NaH, 4 eq BnBr, DMF, 16 h; (g) 12 eq $Me_3N$-$BH_3$, 12 eq $AlCl_3$, THF, molecular sieves (4 Å), 16 h; (h) 1 eq benzaldehyde dimethyl acetal, 0.05 eq camphorsulfonic acid, DMF, 90 °C, 15 min; (i) $Ac_2O$/pyridine 1/1, 24 h; (j) 12 eq NaOMe, MeOH, 1 h; (k) 15 eq NaH, 12 eq BnBr, 0.2 eq TBAI, 0 °C-rt, 16 h; (l) TFA/$CH_2Cl_2$/MeOH 1/3/9, 120 h.

128.3 (CH), 128.1 (CH), 128.0 (CH), 127.9 (CH), 127.8 (CH), 127.7 (CH), 127.6 (CH), 127.6 (CH), 127.6 (CH), 127.5 (CH), 127.4 (CH), 94.4 (CH), 94.2 (CH), 81.7 (CH), 81.6 (CH), 79.5 (CH), 79.3 (CH), 77.6 (CH), 75.6 ($CH_2$), 75.5 ($CH_2$), 75.1 ($CH_2$), 75.1 ($CH_2$), 72.9 ($CH_2$), 72.8 ($CH_2$), 71.2 (CH), 70.7 ($CH_2$), 70.6 (CH), 61.5 ($CH_2$), 59.1 ($CH_3$) ppm. IR (HATR): 3479 (w, br), 3059 (w), 3029 (w), 2920 (w), 2873 (w), 1496 (w), 1453 (m), 1396 (w), 1359 (m), 1328 (w), 1208 (w), 1153 (m), 1133 (m), 1088 (s), 1068 (s), 1027 (m), 990 (s), 920 (m), 847 (w), 798 (w), 732 (s), 695 (s), 641 (w) $cm^{-1}$. ESMS [m/z (fragment, intensity), API-ES positive mode]: 914.4 ($M+NH_4^+$, 100). HRMS (ESI-TOF): calcd. for $C_{55}H_{64}NO_{11}$ $[M+NH_4]^+$ 914.4474; found 914.4493.

### *2,3,4,2′,3′,4′-Hexa-O-benzyl-6-O-methyl-6′-O-[N-(2-phenethyl)carbamoyl]-α,α-D-trehalose (33)*

General procedure B was applied (use of phenethyl isocyanate). Purification via column chromatography (gradient elution: hexane/EtOAc 8/2 to 6/4) afforded the title compound **33** as a colourless glass (280 mg, 0.268 mmol, 98%).

Rf 0.13 in hexane/EtOAc 7/3. $^1H$ NMR (400 MHz, $CDCl_3$): $\delta$ 7.40–7.10 (m, 35H), 5.19 (d, $J=3.4$ Hz, 1H), 5.15 (d, $J=3.4$ Hz, 1H), 5.01 (d, $J=11.2$ Hz, 1H), 4.98 (d, $J=11.6$ Hz, 1H), 4.92–4.77 (m, 4H), 4.73–4.49 (m, 7H), 4.31–3.95 (m, 6H), 3.68–3.25 (m, 8H), 3.27 (s, 3H), 2.75 (t, $J=7.0$ Hz, 2H) ppm. $^{13}C$ NMR (100 MHz, $CDCl_3$): $\delta$ 155.9 (C), 138.9 (C), 138.7 (C), 138.6 (C), 138.4 (C), 138.1 (C), 137.9 (C), 128.7 (CH), 128.6 (CH), 128.4 (CH), 128.4 (CH), 128.4 (CH), 128.4 (CH), 128.3 (CH), 128.0 (CH), 127.9 (CH), 127.8 (CH), 127.7 (CH), 127.7 (CH), 127.6 (CH), 127.5 (CH), 127.4 (CH), 126.5 (CH), 94.7 (CH), 94.3 (CH), 81.7 (CH), 81.6 (CH), 79.3 (CH), 79.1 (CH), 75.6 ($CH_2$), 75.5 ($CH_2$), 75.1 ($CH_2$), 72.9 ($CH_2$), 72.7 ($CH_2$), 70.6 ($CH_2$), 70.6 (CH), 69.5 (CH), 63.1 ($CH_2$), 59.1 ($CH_3$), 42.1 ($CH_2$), 36.0 ($CH_2$) ppm. IR (HATR): 3410 (w), 3341 (w), 3086 (w), 3059 (w), 3026 (w), 2920 (w), 2877 (w), 1722 (m), 1514 (w), 1496 (m), 1454 (m), 1401 (w), 1359 (w), 1328 (w), 1242 (m), 1208 (w), 1153 (m), 1086 (m), 1068 (s), 1027 (m), 991 (s), 911 (w), 850 (w), 797 (w), 733 (s), 695 (s), 640 (w) $cm^{-1}$. ESMS [m/z (fragment, intensity), API-ES positive mode]: 1061.4 ($M+NH_4^+$, 100). HRMS (ESI-TOF): calcd. for $C_{64}H_{73}N_2O_{12}$ $[M+NH_4]^+$ 1061.5158; found 1061.5179.

### *6-O-Methyl-6′-O-[N-(2-Phenethyl)carbamoyl]-α,α-D-trehalose (34)*

General procedure C was applied. Purification via column chromatography (gradient elution: $CH_2Cl_2$/MeOH 9/1 to 7/3) afforded the title compound **34** as a white glass (96 mg, 0.191 mmol, 83%).

Rf 0.49 in $CH_3CN/H_2O$ 8/2, 0.17 in $CH_2Cl_2$/MeOH 8/2. $^1H$ NMR (400 MHz, $CD_3OD$): $\delta$ 7.32–7.13 (m, 5H), 5.14–4.99 (m, 2H), 4.36–4.14 (m, 2H), 4.08–3.88 (m, 2H), 3.83–3.71 (m, 2H), 3.66–3.52 (m, 2H), 3.51–3.40 (m, 2H), 3.37 (s, 3H), 3.35–3.26 (m, 4H), 2.77 (t, $J=7.4$ Hz, 2H) ppm. $^{13}C$ NMR (100 MHz, $CD_3OD$): $\delta$ 159.1 (C), 140.5 (C), 129.8 (CH), 129.5 (CH), 127.3 (CH), 95.3 (CH), 74.5 (CH), 74.5 (CH), 73.1 (CH), 73.1 ($CH_2$), 72.5 (CH), 72.0 (CH), 71.8 (CH), 64.9 ($CH_2$), 59.5 ($CH_3$), 43.5 ($CH_2$), 37.1 ($CH_2$) ppm. IR (HATR): 3308 (m, br), 2926 (w), 1697 (m), 1526 (w), 1496 (w), 1455 (w), 1334 (w), 1252 (m), 1197 (w), 1146 (m), 1101 (m), 1074 (m), 1037 (m), 1018 (m), 987 (s), 943 (m), 909 (w), 856 (w), 807 (w), 772 (w), 750 (w), 700 (w) $cm^{-1}$. $[\alpha]_D^{22}$ $+132°$ (c 0.10, MeOH). ESMS [m/z (fragment, intensity), API-ES positive mode]: 504.2 ($M+H^+$, 100). HRMS (ESI-TOF): calcd. for $C_{22}H_{33}NNaO_{12}$ $[M+Na]^+$ 526.1895; found 526.1879.

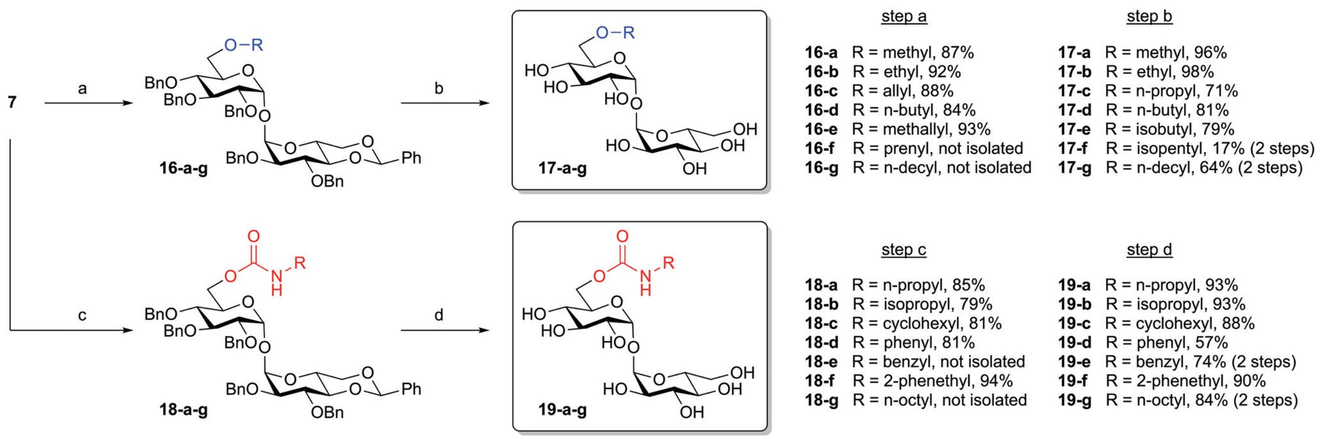

Scheme 2. Synthesis of 6-*O*-substituted trehalose derivatives. Reagents and conditions: (a) 2.5 eq NaH, 4 eq R-Br or R-I, DMF, 16 h; (b) 0.05 eq Pd/C, EtOAc/MeOH 1/1, 16 h; (c) 3 eq R-N = C = O, 0.2 eq DMAP, $CH_2Cl_2$, 24–120 h.

### *Enzyme activity assays*

Trehalose and each derivative (20 mM) were incubated with 0.01 mg $ml^{-1}$ trehalase from porcine kidney (Sigma Aldrich) or *Mycobacterium smegmatis* (NZYtech) in 100 mM of sodium phosphate buffer (pH 7) at 37 °C for 24 h. Mixtures with *Mycobacterium smegmatis* trehalase also contained 6 mM of $MgSO_4$, as the enzyme requires $Mg^{2+}$ for activity[32]. Samples were taken at regular time intervals and diluted with ultrapure water for analysis with high performance anion-exchange chromatography with pulsed amperometric detection (HPAEC-PAD, Dionex ICS-3000).

In order to evaluate their relative inhibition percentage, each trehalose derivative (20 mM) was incubated with 0.01 mg $ml^{-1}$ of above-mentioned trehalases with 3 or 20 mM of trehalose for porcine kidney and *Mycobacterium smegmatis* trehalases, respectively, in 100 mM of sodium phosphate buffer (pH 7) at 37 °C for 14 min. Again, 6 mM of $MgSO_4$ was added to the reaction mixture with *Mycobacterium smegmatis* trehalase. Samples were taken every 2 min and diluted with ultrapure water for analysis with HPAEC-PAD. The residual activity of the enzyme towards trehalose in each mixture was determined by comparing the measured activity to the activity determined in the absence of trehalose derivative.

For selected compounds **2**, **17-a**, **19-d**, **19-e**, **19-f**, **21-a**, **23-f**, and **31**, $IC_{50}$ values were determined by incubating different concentrations of the trehalose derivative (0.01–50 mM) with the selected trehalases under the same conditions as mentioned above. Samples were taken every 2 min and diluted for analysis with HPAEC-PAD. The residual activity of the enzyme towards trehalose in each mixture was determined compared to a sample without trehalose derivative. Plots and curve fits were obtained via Sigmaplot 13. All reactions were performed in triplicate, values are represented as the mean and standard deviations were in the range of ≤15% of the reported value.

### *Molecular modelling*

All manipulations were performed with the molecular modelling program YASARA and the YASARA/WHATIF twinset and figures were created with PyMOL 2.0. The ligand free and inhibitor bound crystal structures of trehalase from *Enterobacter cloacae* (GH37 family) (PDB code 5Z6H and 5Z66, respectively)[33] were used as template for docking. Trehalose derivative structures were created with YASARA Structure and subsequently minimised with the AMBER03 force field. The grid box for docking had a dimension of 25 × 25 × 25 Å and comprised the entire catalytic cavity. Docking was performed with AutoDock VINA using default parameters and ligands were allowed to rotate freely during the simulation. The conformer with an appropriate glucose in both the −1 and +1 subsite was selected as the binding mode for further analysis.

## Results

### *Chemistry*

### *Synthesis of strategic intermediates*

Generally, synthesis of all final analogues was achieved from a central set of partially protected strategic intermediates **7**–**10**, **12** and **15** which could be obtained from trehalose **1** (Scheme 1). After protection of both 4- and 6-hydroxyl groups in trehalose as benzylidene acetal, the remaining OH functional groups were converted to the corresponding benzyl ethers, leading to **6**[27]. In the following crucial desymmetrisation step, regioselective reductive opening of one of the acetal moieties to either the 4- or 6-mono-alcohol can be achieved using diisobutylaluminium hydride (DIBAL-H) in an appropriate solvent, according to existing literature[27]. Based on reported conditions, selective opening towards strategic intermediate **7** containing a free 6-OH group was achieved through reaction in toluene. The alternative 4-OH isomer **8** could be obtained via DIBAL-H reduction in dichloromethane: in a typical experiment, starting material still remained at moment of quenching (10% recovered), while in addition to the desired compound **8** (55%), regio-isomer **7** (19%) and product **9** (9%, resulting from overreduction) were also isolated through tedious chromatographic separation. Compound **9** was found to be useful as an additional strategic intermediate to access 4/4′-bis-substituted derivatives; the corresponding 6/6′-bis-OH intermediate **10** was obtained after a second DIBAL-H treatment of **7** in toluene. To avoid the above-mentioned practical issues concerning synthesis of **8**, we decided to additionally synthesise alternative strategic intermediate **12**, also allowing the subsequent synthesis of 4-substituted derivatives. This was achieved via sequential *O*-benzylation of **7** and opening of the resulting mono-benzylidene acetal **11** using $Me_3N\text{-}BH_3/AlCl_3$[34]. Under the latter conditions, the desired 4-OH isomer **12** could be isolated in 49% yield, while the fraction of corresponding 6-OH regio-isomer amounted to 38% (compound not shown). Alternatively, intermediate **11** could be obtained via the mono-benzylidene acetal-protected **13**. Although this was expected to involve a shorter reaction route from trehalose, an extra acetylation/deacetylation sequence via **14** was found to be necessary to facilitate intermediate purification, leading to

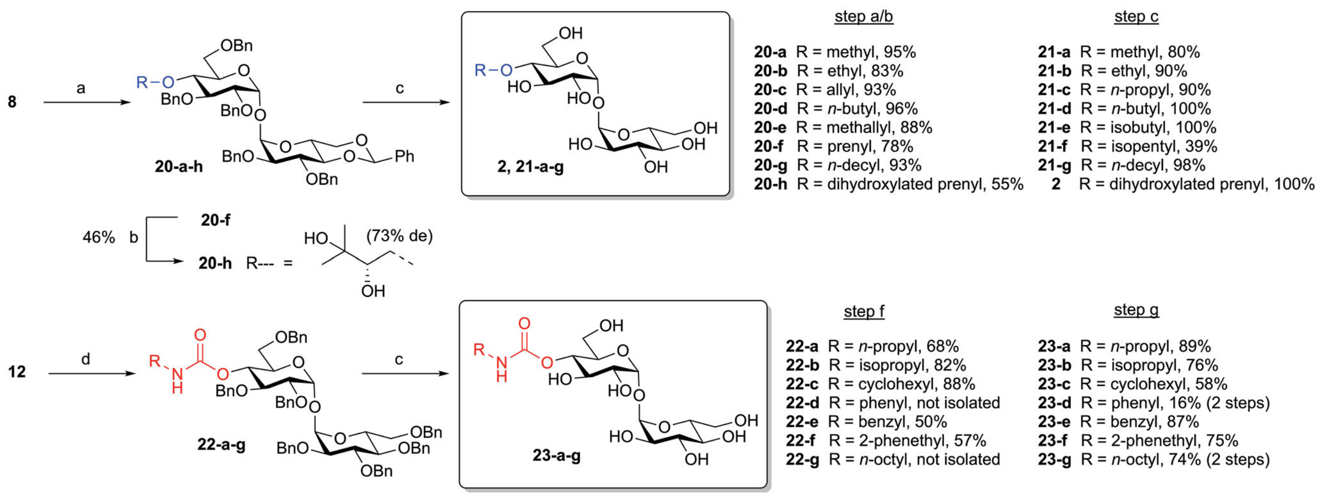

**Scheme 3.** Synthesis of 4-*O*-substituted trehalose derivatives. Reagents and conditions: (a) 2.5 eq NaH, 4 eq R-Br or R-I, DMF, 16 h; (b) AD-mix α, 2.5 eq $MeSO_2NH_2$, 0.25 eq $(DHQ)_2PHAL$, tBuOH/$H_2O$:acetone 1/1/0.9, 240 h; (c) 0.05 eq Pd/C, EtOAc/MeOH 1/1, 16 h; (d) 3 eq R-N = C = O, 0.2 eq DMAP, $CH_2Cl_2$, 24–120 h.

an equal number of reaction steps but significantly lower overall yield. Finally, acid hydrolysis of the acetal moiety in **11** was used to obtain 4/6-bis-OH strategic intermediate **15**. With these strategic intermediates in hand, different sets of 4- or 6-monosubstituted, and 4/4′-, 6/6′- and 4/6-double substituted analogues were accessible, via derivatisation of the remaining unprotected alcohol moieties.

### Derivatisation of strategic intermediates to final compounds

First, using strategic intermediate **7**, two series of 6-*O*-monosubstituted derivatives were prepared (Scheme 2). On one hand, *O*-alkylation using alk(en)yl halogenides with increasing chain length under standard conditions delivered intermediates **16-a–g**, which were overall deprotected using catalytic hydrogenolysis to obtain the intended 6-*O*-alkylated trehalose derivatives **17-a–g**. On the other hand, a series of 6-*O*-carbamoyl derivatives **19-a–g** was prepared via treatment of **7** with a set of isocyanates in presence of DMAP, followed by hydrogenolysis.

Next, two analogous series of 4-*O*-substituted trehalose derivatives were envisaged (Scheme 3). Indeed, alkylation of **8** using the same set of alk(en)yl halogenides mentioned above gave the expected ethers **20-a–g** which were deprotected to the final derivatives **21-a–g**. Additionally, lentztrehalose A **2** was synthesised following reported conditions, via Sharpless asymmetric dihydroxylation of 4-*O*-prenylated intermediate **20-f** and subsequent hydrogenolysis of **20-h**[30]. From $^1$H-NMR analysis, a diastereomeric excess of 73% **20-h** could be determined (Figure S1 in Supplementary Information). Both diastereomers could not be separated chromatographically and the resulting lentztrehalose A **2** was therefore used as such for trehalase evaluation. In analogy to the 6-*O*-series **19-a–g**, we also prepared the corresponding 4-*O*-carbamates **23-a-g** in two steps from strategic intermediate **12**.

Finally, a selection of double-substituted analogues was prepared (Scheme 4). To this end, the appropriate bis-OH strategic intermediates **10**, **9** and **15** were reacted with MeI and subsequently deprotected to obtain the 6/6′-, 4/4′- and 4/6-bis-*O*-methyl derivatives **25**, **29** and **31**, respectively. Additionally, 6/6′-bis-phenethylcarbamoyl trehalose **27** was obtained from **10**. Lastly, mixed derivative **34** was obtained via methylation of intermediate **16-a**, followed by regioselective acetal opening using DIBAL-H in toluene, conversion to the carbamate **33** and final debenzylation.

## Biological evaluation

### Resistance to trehalase hydrolytic activity

The hydrolytic resistance of each trehalose derivative was evaluated by incubating 20 mM of substrate with 0.01 mg $ml^{-1}$ of enzyme at pH 7 and 37 °C for 24 h. No degradation was observed for the majority of derivatives, whereas trehalose was already broken down completely after just 12 h (data not shown). The only exceptions showing significant hydrolysis were derivatives **17-a** (7 and 8% relative hydrolysis by the trehalase from porcine kidney and *M. smegmatis*, respectively), **17-b** (1 and 6%, respectively) and **17-c** (1% and 1%, respectively), which all contain a small alkyl group (i.e. methyl, ethyl and *n*-propyl, respectively) on the 6-*O*-position.

### Inhibitory activity towards trehalase

The inhibitory activity of the compounds was evaluated at a concentration of 20 mM, using trehalose as substrate at a concentration equal to the respective Michaelis–Menten constant (3 mM and 20 mM for porcine kidney and *M. smegmatis* trehalase, respectively). Despite their resistance to hydrolysis, several of these compounds were found to still bind quite tightly to these trehalases, causing enzyme inhibition (Table 1).

Regarding porcine kidney trehalase, the most interesting inhibitors are grouped within the series of 6-*O*-substituted derivatives, as their counterparts with 4-*O*-substitutions are all poor inhibitors (≤35% inhibition at 20 mM). Only two derivatives, namely **17-a** and **19-f**, exhibited complete inhibition. However, these compounds bear very different substituents in both size and chemical properties, i.e. methyl ether and phenethyl carbamate, respectively. Compared to **17-a**, extension of the alkyl ether substituent resulted in a clear drop in inhibition. Similar to compound **19-f**, the other phenyl group-containing carbamates, **19-d** and **19-e**, exhibited significant inhibitory effects (>80%). Compounds in the carbamate series devoid of a phenyl moiety, i.e. **19-a**, **19-b**, **19-c** and **19-g**, showed a clearly lowered inhibition, suggesting that the aromatic group forms a crucial interaction with the enzyme. Interestingly, compared to **17-a** and **19-f**, the additional introduction of either of their substituents on the 6′-*O*-position, i.e. leading to **25**, **27** and **34**, led to a lower rather than a higher affinity. Likewise, the introduction of an additional methyl substituent on the 4-*O*-position in **31** led to a similar lowered inhibition outcome.

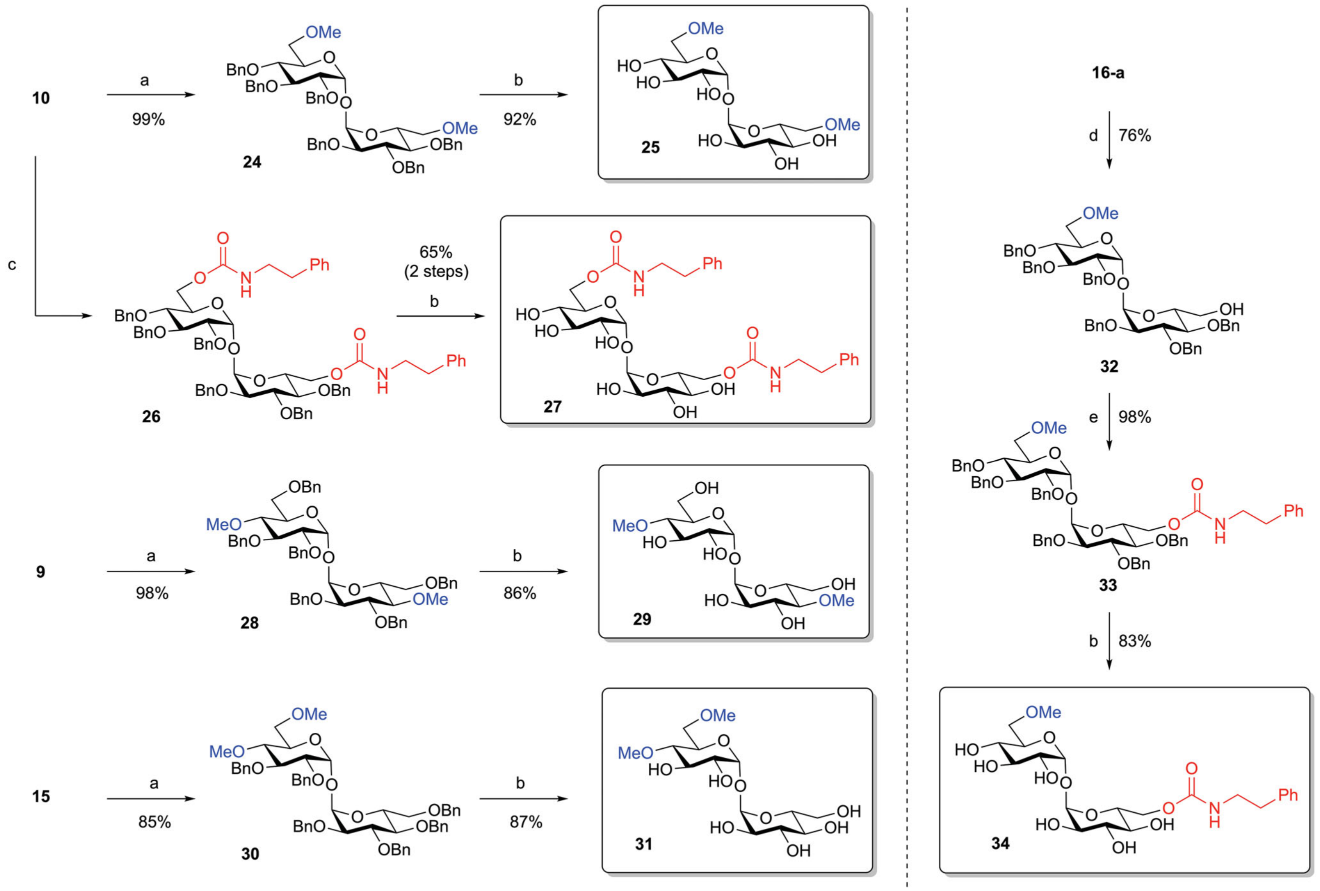

**Scheme 4.** Synthesis of double-substituted trehalose derivatives. Reagents and conditions: (a) 5 eq NaH, DMF, 0 °C, 15 min; then: 8 eq MeI, DMF, 0 °C-rt, 16 h; (b) 0.05 eq Pd/C, EtOAc/MeOH 1/1, 16 h; (c) 6 eq 2-phenethyl isocyanate, 0.4 eq DMAP, $CH_2Cl_2$, 48 h; (d) 5 eq DIBAL-H, toluene, 48 h; (e) 3 eq 2-phenethyl isocyanate, 0.2 eq DMAP, $CH_2Cl_2$, 72 h.

**Table 1.** Inhibition (%) of porcine kidney trehalase (3 mM trehalose) and *M. smegmatis trehalase* (20 mM trehalose) in the presence of 4- (left) and 6-*O*-substituted (right) derivatives of trehalose (20 mM).

| | | 4-position | | | 6-position | |
|---|---|---|---|---|---|---|
| Substituent | Cpd | Porcine kidney (%)[a] | *M. smegmatis* (%)[a] | Cpd | Porcine kidney (%)[a] | *M. smegmatis* (%)[a] |
| -O(CH$_2$)$_2$OHCH(CH$_3$)$_2$OH | **2** | 1 | 100 (93[b]) | – | – | – |
| -OCH$_3$ | **21-a** | 20 | 90 | **17-a** | 100 (4[b]) | 100 (76[b]) |
| -OCH$_2$CH$_3$ | **21-b** | 7 | 82 | **17-b** | 45 | 94 |
| -O(CH$_2$)$_2$CH$_3$ | **21-c** | 3 | 88 | **17-c** | 47 | 82 |
| -O(CH$_2$)$_3$CH$_3$ | **21-d** | 35 | 31 | **17-d** | 36 | 84 |
| -OCH$_2$CH(CH$_3$)$_2$ | **21-e** | 0 | 40 | **17-e** | 24 | 95 |
| -O(CH$_2$)$_2$CH(CH$_3$)$_2$ | **21-f** | 0 | 10 | **17-f** | 7 | 96 |
| -O(CH$_2$)$_9$CH$_3$ | **21-g** | 0 | 0 | **17-g** | 28 | 0 |
| -OC(=O)NH(CH$_2$)$_2$CH$_3$ | **23-a** | 0 | 24 | **19-a** | 23 | 83 |
| -OC(=O)NHCH$_2$(CH$_3$)$_2$ | **23-b** | 0 | 44 | **19-b** | 17 | 67 |
| -OC(=O)NHCy | **23-c** | 0 | 100 (38[b]) | **19-c** | 5 | 78 |
| -OC(=O)NHPh | **23-d** | 32 | 100 (51[b]) | **19-d** | 83 | 100 (44[b]) |
| -OC(=O)NHCH$_2$Ph | **23-e** | 18 | 87 | **19-e** | 90 | 100 (47[b]) |
| -OC(=O)NH(CH$_2$)$_2$Ph | **23-f** | 8 | 93 | **19-f** | 100 (12[b]) | 100 (74[b]) |
| -OC(=O)NH(CH$_2$)$_7$CH$_3$ | **23-g** | 0 | 0 | **19-g** | 26 | 0 |
| 4,4′-bis-OCH$_3$ | **29** | 10 | 100 (43[b]) | – | – | – |
| 6,6′-bis-OCH$_3$ | – | – | – | **25** | 47 | 91 |
| 4,6-bis-OCH$_3$ | **31** | 16 | 100 (88[b]) | **31** | 16 | 100 (88[b]) |
| 6,6′-bis-OOCNH(CH$_2$)$_2$Ph | – | – | – | **27** | 0 | 100 (42[b]) |
| 6-OCH$_3$,6′-OC(=O)NH(CH$_2$)$_2$Ph | – | – | – | **34** | 7 | 98 |

[a]Mean from three different assays, analysis with HPAEC-PAD (errors were in the range of ≤15% of the reported value).
[b]% Inhibition at inhibitor concentration of 1 mM.

Towards *M. smegmatis* trehalase, contrarily, members of both series of 4- and 6-*O*-substituted compounds generally demonstrated interesting inhibitory properties at a concentration of 20 mM. Whereas high inhibition is observed for 6-*O*-alkyl derivatives (in contrast to the somewhat lower activity of the corresponding 4-*O*-alkyl analogues), carbamate derivatives bearing a cyclohexyl, phenyl, benzyl or phenethyl group on either the 4- or 6-*O*-position (**19-c-f**, **23-c-f**) all cause a high to complete inhibition. Furthermore, all 4,4′-, 6,6′- and 4,6-bis-substituted derivatives show an inhibition percentage that exceeds 90%. Compounds

**Table 2.** $IC_{50}$ values of selected trehalose derivatives for porcine kidney and *M. smegmatis* trehalase.

| | $IC_{50}$ (mM) | |
|---|---|---|
| Compound | Porcine kidney trehalase | *M. smegmatis* trehalase |
| 2 | ≫20[a] | 0.67 |
| 17-a | 8.61 | 0.71 |
| 19-d | >20[a] | 1.11 |
| 19-e | >20[a] | 1.03 |
| 19-f | 9.91 | 0.74 |
| 21-a | >20[a] | 5.27 |
| 23-f | >20[a] | 5.11 |
| 31 | >20[a] | 0.35 |

[a]Value not determined, based on observed weak inhibition at 20 mM (see Table 1).

with longer (*n*-octyl/*n*-decyl) hydrophobic chains at the 4- or 6-*O*-positions (**17-g**, **19-g**, **21-g** and **23-g**), resulted in a lack of inhibitory effect towards *M. smegmatis* trehalase.

To further rank the derivatives that caused complete inhibition, the abovementioned experiments were repeated with a lower inhibitor concentration, i.e. 1 mM instead of 20 mM (Table 1). Additionally, eight trehalose derivatives were also subjected to half maximal inhibitory concentration ($IC_{50}$) experiments (Table 2). This confirmed that compounds **17-a** and **19-f** are the strongest inhibitors for porcine kidney trehalase, while derivatives **2**, **17-a**, **19-f** and **31** displayed sub-mM $IC_{50}$ values against *M. smegmatis* trehalase. Interestingly, the $IC_{50}$ of the strongest inhibitors of porcine kidney trehalase is about 10 mM whereas substantially lower values (down to 0.35 mM) are found for the *M. smegmatis* trehalase.

## *Molecular docking studies*

To clarify the displayed activities of the new derivatives, molecular docking studies were carried out. Unfortunately, no structural elucidation was possible for the enzyme from *M. smegmatis* as no crystal structure of a trehalase from family GH15 has yet been determined. However, several crystal structures of trehalases from family GH37 are described in literature, namely from *Escherichia coli*, *Saccharomyces cerevisiae* and *Enterobacter cloacae*[33,35–37]. Interestingly, the latter has been crystallised in two different forms, i.e. with and without the inhibitor validoxylamine A **36**, which resulted in structures that can be described as 'closed' and 'open', respectively. Indeed, the so-called lid loop ($G^{506}$-$G^{519}$) and side loop ($Y^{147}$-$Y^{159}$) were found to undergo a significant conformational change upon ligand binding (Figure 2)[33]. Docking simulations were performed with these structures as representative scenario for family GH37 trehalases, such as the one from porcine kidney. The bacterial enzyme shares about 30% sequence identity and 40% similarity with porcine and human trehalase, but all active site residues within a distance of 4 Å from validoxylamine A **36** are fully conserved (Supplementary Figure S2). It was hypothesised that a compound sensitive to hydrolysis should find a productive docking pose in both the open and closed conformation, whereas a hydrolysis-resistant inhibitor should only be accommodated by the former.

After removal of the cocrystallised validoxylamine A **36**, trehalose **1** was docked in the active site pocket of the closed structure and compared with the reference compound **36** (Figure 2, bottom). A very good overlay was observed after superposition, with most of the crucial interactions being conserved. A similar result was obtained after docking of trehalose **1** and validoxylamine A **36** in the open structure (Figure 2, top). Subsequently, the binding of various trehalose derivatives was simulated (Figure 3; Table 3).

Compound **21-a** (4-*O*-methyltrehalose) was picked as a 4-*O*-substituted representative in order to find an explanation for their low inhibitory activity and high resistance to hydrolysis. In the open form, two docking poses were found, i.e. with the methyl substituent positioned in either subsite −1 or +1. However, neither of these fits match with the one of trehalose, and very little interactions are formed between enzyme and ligand (Figure 3). There obviously is little space around the 4-hydroxy position of trehalose in both subsite −1 and +1 (Figure 2), explaining why the bulkier compounds cannot take on the same pose as trehalose. Furthermore, no productive docking result could be obtained in the closed structure.

In contrast to their 4-*O*-substituted counterparts, 6-*O*-substituted derivatives show a far more interesting inhibitory profile. A distinction can be made between the ether and carbamate derivatives. For example, the 6-methoxy derivative **17-a** triggered minor hydrolytic activity as well as an attractive inhibitory effect. Docking simulations confirmed our hypothesis that the derivative could find a productive conformation in both the open and closed structure (Figure 3). Interestingly, the substitution is accommodated in subsite −1 rather than +1, although the latter seems to offer more space (Figure 2). In contrast, substitutions containing four or more carbons are preferentially positioned in subsite +1, although their inhibitory potential is considerably lower. These findings suggest that a greater affinity can be achieved when the substituent is sufficiently small to be placed in subsite −1.

A trend could be noticed within the 6-*O*-carbamate derivatives, as the inhibitory potential among these compounds clearly is highest when a phenyl group is present. For instance, complete inhibition of porcine kidney trehalase without hydrolysis was observed with compound **19-f**, containing a *N*-phenethylcarbamoyl group. This trehalose derivative was able to find a fit in the open active site (Figure 3), while its positioning in the closed structure was predicted to have a negative binding energy (i.e. repulsion, Table 3). A strong stacking interaction is established between the compound's aromatic group and $F^{154}$, and again, the substituent was found to be accommodated in subsite −1. Docking simulations were also performed with both other phenyl-containing carbamates **19-d** and **19-e** and cyclohexyl carbamate **19-c** (Figure 3). The positioning of the benzyl substituent of **19-e** resembles that of **19-f**. Remarkably, the phenyl carbamate moiety of **19-d** is preferentially placed in the +1 subsite instead, although the interaction with $F^{154}$ still is retained. Just as with the alkylated derivatives, the positioning of the substituent in subsite +1 appears to lower the compound's inhibitory potential. In contrast, the cyclohexyl carbamate **19-c** exhibited a different behaviour: its preferred docking poses do not match the one of trehalose, highlighting the importance of the aromatic group.

## Discussion

In our work, we have evaluated a series of 4- and/or 6-*O*-substituted trehalose derivatives towards trehalase degradation. Generally, all compounds were resistant to hydrolysis for both tested trehalases, with the exception of 6-*O*-alkylated derivatives with short carbon chain length ($C_1$-$C_3$). For porcine kidney trehalase, as representative of GH37 trehalases, this trend was confirmed by docking studies: the hydrolysable 6-*O*-substituted derivatives could find a productive fit in the closed (i.e. hydrolytically active) enzyme structure, whereas that was not the case for analogues containing larger substituents at this position or for any of the 4-substituted derivatives. This is consistent with the

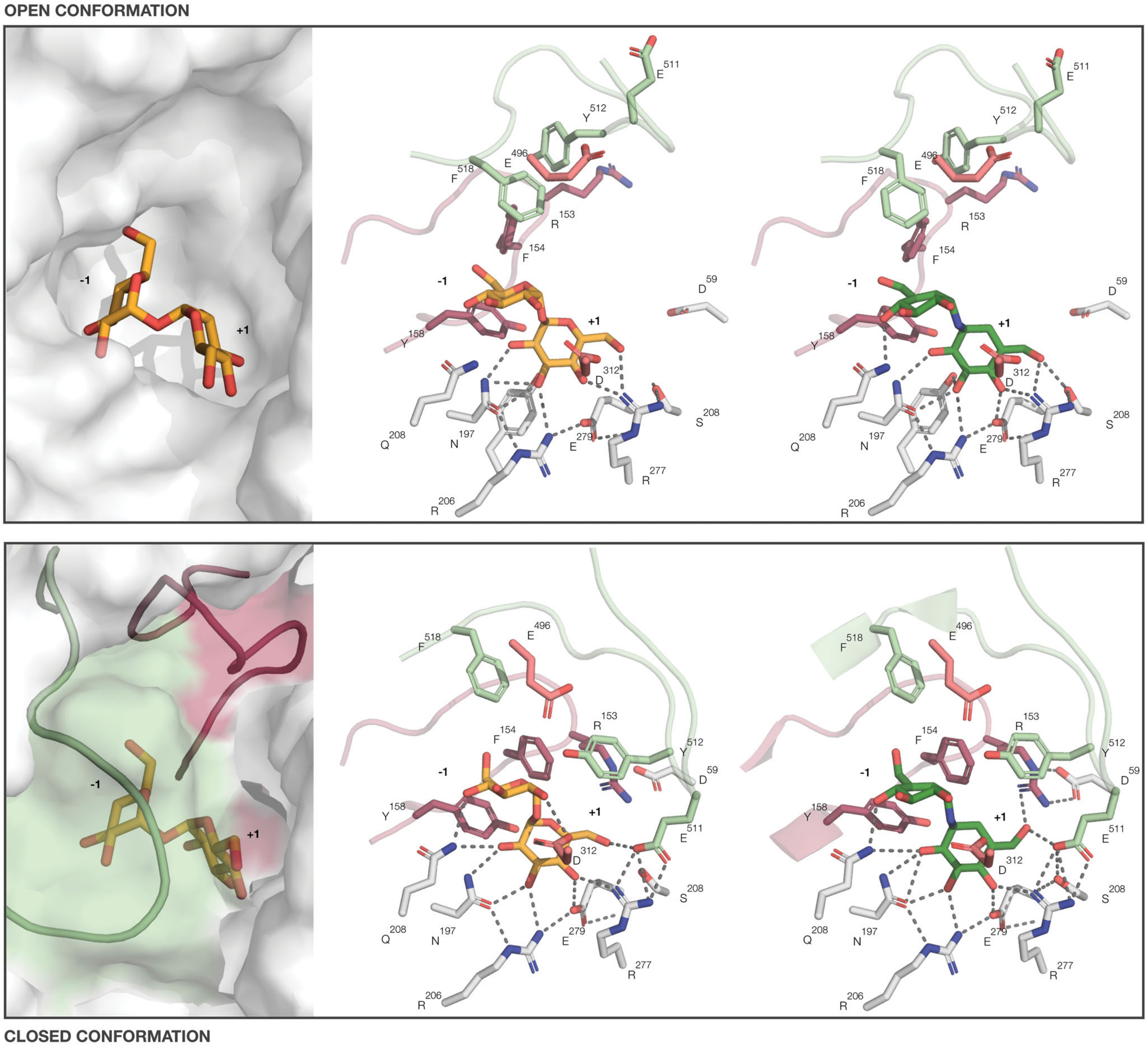

**Figure 2.** Docking of trehalose **1** (left, orange) and validoxylamine **36** (right, green) in the active site pocket of the open enzyme conformation (upper) and the closed conformation (lower) with surface view of active site pocket (catalytic residues $D^{312}$ and $E^{496}$, salmon; side loop $Y^{147}$-$Y^{159}$, raspberry; lid loop $G^{506}$-$G^{519}$, green).

findings of Asano et al., who pointed out that the enzyme is most tolerant towards substitutions at the 6-*O*-position[38].

To evaluate whether the non-hydrolysable trehalose derivatives are still able to bind to the enzyme, inhibition experiments were performed with the trehalases from porcine kidney (family GH37) and *M. smegmatis* (family GH15).

From a pharmaceutical perspective, inhibitors of intestinal glycosidases, including trehalase (GH37 family), can be interesting and may be orally administered in the treatment of diabetes (type II) to regulate the absorption of carbohydrates[39]. A distinction can be made between transition state analogues and substrate analogues[40]. The former are inhibitors that optimally bind the transition state of trehalase, by mimicking the glucosyl-oxocarbenium ion which is developing during enzymatic action[35,40]. Examples are validamycin A **35**, validoxylamine A[41] **36** and trehazolin[35] **37**, which have $IC_{50}$ values in the nanomolar range for porcine kidney trehalase (Figure 4). In contrast, substrate analogues like mannotrehalose[42,43] **38**, 5-thiotrehalose[42] **39** and 3,3′-diketotrehalose[44] **40** are mildly potent inhibitors with $IC_{50}$ values in the range of 0.1–1 mM (Figure 4). In comparison, as substrate analogue inhibitors, the presented 6-substituted trehaloses **17-a** and **19-f** have $IC_{50}$ values around 9 mM. The observed inhibition could be explained via docking experiments: binding of **17-a** and **19-f** was shown to be possible in the open state of the enzyme via placement of the side chain in the −1 subsite.

Trehalases could also be attractive drug targets in the ongoing battle against pathogenic microorganisms due to the essentiality of trehalose and its metabolic derivatives for their survival[23]. Trehalose is a building block of the cell wall in mycobacteria and corynebacteria, as it is a basic component of their glycolipids[6,10]. For example, the cell wall of the pathogen *Mycobacterium tuberculosis* contains trehalose-6,6′-dimycolate, a toxic lipid that has been identified as the main virulence factor of tuberculosis and is responsible for the low permeability of the cell wall, leading to drug resistance[10,45]. Interestingly, Shleeva and co-workers investigated the importance of trehalase activity on the resuscitation of dormant mycobacterial cells[46]. Validamycin A **35** (Figure 4), a trehalase inhibitor that mimics the transition-state, had a negative

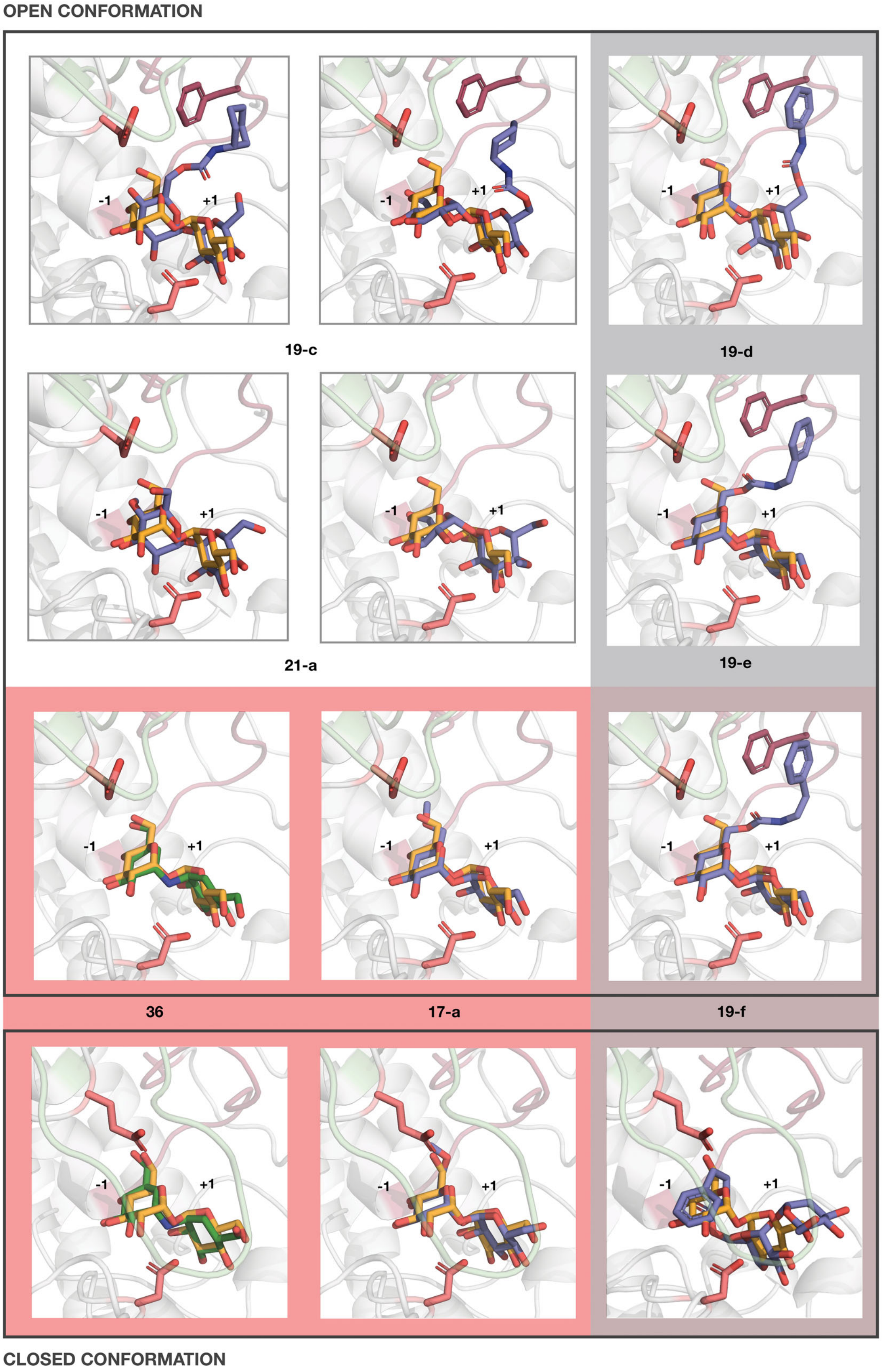

Figure 3. Docking of trehalose derivatives (blue) and validoxylamine **36** (green) in the active site pocket of the open (upper) and closed (bottom) enzyme conformation. An overlay of trehalose (orange) was added for reference. Salmon rectangle: compounds docked in both open and closed conformations, grey rectangle: compounds with aromatic group (catalytic residues $D^{312}$ and $E^{496}$, salmon; side loop $Y^{147}$-$Y^{159}$, raspberry; lid loop $G^{506}$-$G^{519}$, green).

effect on the resuscitation of dormant *Mycobacterium smegmatis* cells, hereby highlighting the importance of trehalose breakdown for cell revival[46]. Based on our obtained results, trehalose derivatives **2**, **17-a**, **19-d**, **19-e**, **19-f**, **23-a**, **23-c**, **23-d**, **27**, **29** and **31**, exhibiting complete inhibition of *M. smegmatis* trehalase at an inhibitor concentration of 20 mM, are suggested to be mildly

**Table 3.** Binding energy (kcal $mol^{-1}$) and the possible presence of a productive conformation determined by simulated molecular docking experiments versus experimentally observed hydrolysis and inhibition.

| Cpd | Binding energy open | Productive conformation? | Inhibition?[a] | Binding energy closed | Productive conformation? | Hydrolysis? |
|---|---|---|---|---|---|---|
| **1** | 6.88 ± 0.79 | YES | – | 3.36 ± 3.61 | YES | YES |
| **2** | 7.39 ± 0.47 | NO | NO | −1.17 ± 2.63 | NO | NO |
| **17-a** | 7.21 ± 0.75 | YES | YES | 0.81 ± 6.99 | YES | YES |
| **19-c** | 8.33 ± 0.48 | NO | NO | −2.06 ± 1.24 | NO | NO |
| **19-d** | 9.02 ± 0.55 | YES | YES | −7.14 ± 2.48 | NO | NO |
| **19-e** | 8.85 ± 0.51 | YES | YES | −5.53 ± 1.06 | NO | NO |
| **19-f** | 8.80 ± 0.54 | YES | YES | −2.42 ± 1.19 | NO | NO |
| **21-a** | 6.76 ± 0.68 | NO | NO | −0.17 ± 4.64 | NO | NO |
| **36** | 7.30 ± 0.71 | YES | YES | 2.84 ± 4.59 | YES | NO[b] |

[a]>80% inhibition percentage (see Table 1).
[b]Non-hydrolysable compound (no glycosidic linkage).

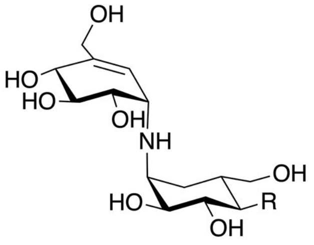

**35**

validamycin A: R = β-D-glucopyranosyl
$IC_{50,PT}$ = 4.2 $10^{-4}$ mM
$IC_{50,MT}$ = 1.8 $10^{-3}$ mM

**36**
validoxylamine A: R = H
$IC_{50,PT}$ = 1.4 $10^{-5}$ mM
$IC_{50,MT}$ = 1.1 $10^{-5}$ mM

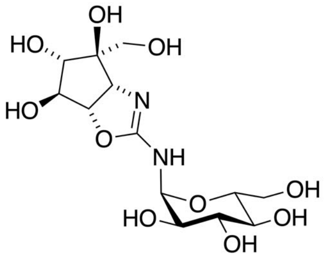

**37**

trehazolin
$IC_{50,PT}$ = 1.9 $10^{-5}$ mM
$IC_{50,MT}$ = no inhibition

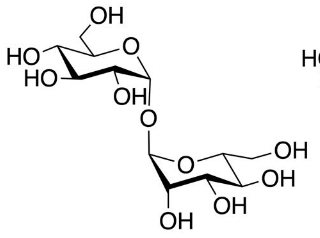

**38**

mannotrehalose
$IC_{50,PT}$ = 1.1 mM

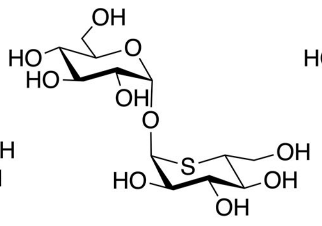

**39**

5-thiotrehalose
$IC_{50,PT}$ = 0.1 mM

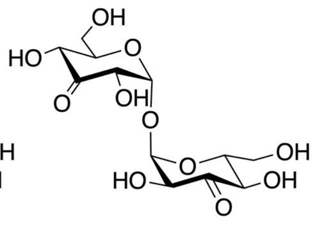

**40**

3,3'-diketotrehalose
$IC_{50,PT}$ = 0.8 mM

**Figure 4.** Structure of trehalose derivatives that are hydrolysis resistant and inhibitors of trehalases ($IC_{50,PT}$ and $IC_{50,MT}$; half maximal inhibitory concentration of porcine kidney and *M. smegmatis* trehalase, respectively).

potent inhibitors with $IC_{50}$ values down to 0.35 mM for **31**. To the best of our knowledge, this is the first report of substrate analogues that inhibit the activity of *M. smegmatis* trehalase.

Finally, clear differences can be noticed between the two studied trehalases concerning interaction with our series of trehalose derivatives; generally, *M. smegmatis* trehalase is more sensitive to inhibitory effects in comparison to porcine kidney trehalase, reflecting their classification in different families (GH15 vs. GH37, respectively). Carrol and co-workers already pointed this out, as it was found that the transition-state mimic trehazolin[35,47], a known inhibitor of porcine kidney trehalase, had no inhibitory effect towards *M. smegmatis* trehalase[32] (Figure 4). In our study, this effect is particularly noticeable in the performance of 4-*O*-substituted derivatives; several compounds (**2**, **21-a**, **23-c**, **23-d**, **23-f**, **29**, **31**) show an interesting inhibition of *M. smegmatis* trehalase (>90% inhibition at 20 mM concentration) but lack inhibitory effect towards porcine kidney trehalase. Notably, the trehalase hydrolysis-resistant lentztrehalose A **2** inhibited the activity of *M. smegmatis* trehalase completely, whereas it only had a poor effect on porcine kidney trehalase activity (1% inhibition) at the same concentration, the latter confirming previously reported data[25]. This binding selectivity is also reflected in the $IC_{50}$ values of these 4-*O*-substituted compounds, which are in the lower- to sub-mM range (0.35–5.27 mM) for *M. smegmatis* trehalase, but could not be determined for porcine kidney trehalase (>20 mM). Due to this selective inhibition profile and the hydrolytic stability, these 4-

substituted trehalose derivatives could be investigated as potential selective anti-pathogenic agents that exert a limited inhibitory effect in the human and/or mammalian gastro-intestinal tract without being easily degraded by intestinal trehalases.

## Conclusions

In this study, a series of trehalose derivatives was successfully synthesised and the effects of their substitution pattern was explored towards trehalase susceptibility. All compounds were shown to be fully or strongly resistant to enzymatic hydrolysis, and may thus have a higher bioavailability than trehalose due to their increased resistance against trehalase activity in the human gastro-intestinal tract, which could be promising in eventual drug applications. Amongst all tested analogues, only trehalose derivatives bearing short alkoxyl chains on the 6-*O*-position were marginally hydrolysed. A number of the synthesised compounds were found to have an interesting substantial and selective inhibitory effect against *M. smegmatis* trehalase. Studies on the implication of these trehalose derivatives on the growth of pathogens like *M. tuberculosis* are ongoing.

Docking simulations in a trehalase originating from the GH37 family confirmed our experimental findings on porcine kidney trehalase. None of the 4-*O*-substituted derivatives could find a productive binding conformation in the open enzymatic state which supports their low inhibitory effect; they are resistant against hydrolysis as the closed formation cannot be reached. However, compounds with substituents at the 6-*O*-position showed a greater binding affinity in the open form, explaining their inhibition potential, while a successful fit in the closed form could only be achieved by the hydrolysable derivatives containing small 6-*O*-alkyl groups.

In conclusion, an exploration of 34 trehalose derivatives was performed by investigating a variety of substituents on the 4- and 6-position. Our work reveals their interaction with two relevant trehalases, i.e. one from family GH37 and one from GH15, through *in vitro* and *in silico* experiments. In this way, preliminary steps have been taken to unlock their potential use as therapeutic agents.

## Disclosure statement

No potential conflict of interest was reported by the author(s).

## Funding

This work was supported by Fonds Wetenschappelijk Onderzoek [12ZD821N,S003617N]

## ORCID

Shari Dhaene http://orcid.org/0000-0003-0887-7651
Johan Van der Eycken http://orcid.org/0000-0002-1623-6750
Koen Beerens http://orcid.org/0000-0001-6608-0443
Jorick Franceus http://orcid.org/0000-0003-4377-5933
Tom Desmet http://orcid.org/0000-0002-5788-3022
Jurgen Caroen http://orcid.org/0000-0003-0102-2458
